# The central nervous system adjusts muscle synergy structure and tightly controls rollator-supported transitions between sitting and standing

**DOI:** 10.1186/s12984-025-01622-y

**Published:** 2025-04-25

**Authors:** Michael Herzog, Frieder C. Krafft, Janis Fiedler, Denise J. Berger, Lizeth H. Sloot, Andrea d’Avella, Thorsten Stein

**Affiliations:** 1 BioMotion Center, Institute of Sports and Sports Science, Karlsruhe Institute of Technology (KIT), Engler-Bunte Ring 15, 76131 Karlsruhe, Germany; 2 HEiKA – Heidelberg Karlsruhe Strategic Partnership, Karlsruhe Institute of Technology (KIT), Heidelberg University, Heidelberg, Germany; 3 Institute of Computer Engineering, Heidelberg University, Heidelberg, Germany; 4 Center of Prevention, Diagnostic and Performance, Center of Orthopaedics Hohenlohe, Künzelsau, Germany; 5 Institute of Sports and Sports Science, Karlsruhe Institute of Technology, Karlsruhe, Germany; 6 Laboratory of Neuromotor Physiology, IRCCS Fondazione Santa Lucia, Rome, Italy; 7 Department of Systems Medicine and Centre of Space Bio-Medicine, University of Rome Tor Vergata, Rome, Italy; 8 Translational and Clinical Research Institute, Newcastle University, Newcastle upon Tyne, UK; 9 Department of Biology, University of Rome Tor Vergata, Rome, Italy

**Keywords:** Assistive device, Rollator, Walker, Sit-to-stand, Stand-to-sit, Muscle synergies, Movement primitives

## Abstract

**Background:**

Older individuals are at risk of falling. Assistive devices like rollators help to reduce that risk, especially by compensating for decreased leg muscle strength and balance problems. Paradoxically, rollators have been found to be a fall risk as well as being difficult to use. To investigate the causes, this study examines how different levels of rollator support (no assistance, light touch, and full support) and balance demands (standard lab floor, balance pads) affect movement coordination during standing up and sitting down movements.

**Methods:**

Twenty young participants stood up and sat down while full-body kinematics and muscle activity (30 channels) were recorded. Participants stood up and sat down using different movement strategies (e.g., forward leaning, hybrid, and vertical rise standing up movement strategies). For each movement strategy, spatial and temporal muscle synergies were extracted from the muscle activity patterns. Temporal muscle synergies provided a more compact, low-dimensional representation than spatial muscle synergies, so they were subsequently clustered with k-means++. The activation duration of the temporal muscle synergies was assessed with full-width at half-maximum at the main peak. Multivariate linear mixed models were used to investigate if the muscle weightings associated with the temporal muscle synergies differed across the support conditions.

**Results:**

The timings of the temporal muscle synergy activations, but not the shape, differed across the movement strategies for both types of movement. Across all tasks, temporal muscle synergies showed a narrower width of activation around the time of seat-off and seat-on than at the movement start and end. No support-specific temporal muscle synergies were found, but lower limb muscle weightings decreased while upper-limb muscle weightings increased with increased support.

**Conclusion:**

The narrow shape of the temporal synergy activation profiles suggests that the central nervous system controls the movements tightly, especially around seat-off and seat-on and in challenging conditions with increased balance demands. Furthermore, rollator support increases the weightings of upper body and decreases the weightings of lower limb muscles, especially around seat-off and seat-on. Future studies may further investigate how the loss of tight movement control may cause falls in older individuals.

**Supplementary Information:**

The online version contains supplementary material available at 10.1186/s12984-025-01622-y.

## Background

Worldwide, at least one in four individuals over 65 falls every year [1, 2]. Falls and associated injuries often lead to insecurities and restricted mobility, thereby making daily activities challenging and reducing older people’s independence [1, 3]. Assistive devices, including canes, crutches, walkers, or rollators, are intended to reduce falls by providing stability and facilitating daily activity independence [4]. Rollators, i.e., four-wheeled walkers, are often prescribed to patients who require an assistive device to compensate for muscular weaknesses. By lessening lower limb loading and enhancing balance, they can help alleviate pain and injuries [4–7]*.* Paradoxically, studies have shown that rollators are associated with falls [4, 8]. Also, Mann et al. [7] reported that 57% of the problems with using walkers relate to a “difficult and/or dangerous” use. Although rollators are used widely, the reasons for the difficulties and the increased fall risk have remained unclear due to the lack of thorough biomechanical studies [9].

Rollators are prescribed primarily to help with walking but are also used to stand up and sit down, especially when other assistance, like a handrail or armrest, is missing [10, 11]. Standing up and sitting down are crucial movements to live an independent life but are demanding due to the dynamic balance requirement during the transitions between sitting and standing [11–14]. Lower limb muscular strength and balance are two main predictors of successful sit-to-stand movements [15]. However, these decline with age. In particular, knee extensor strength has been found to decline annually by approximately 2–4% after age 50 [16], and a meta-analysis [17] found lower limb weakness to be a statistically significant risk factor for falls. A rollator may help by providing load transfer from the lower limbs to the upper body, reducing the strength demand for the hip and knee extension musculature [4, 18]. Furthermore, the additional contact points through the rollator handles might provide extra positional information next to the feet and enlarge the base of support (BoS), potentially improving balance [4, 19]. However, the evidence is not clear. While a related study found that rollator support increases movement stability during standing up and sitting down movements in young adults [20], a recent observational study in a long-term care setting found that 44.4% of the falls while using a rollator occurred while transitioning between sitting and standing [21]. A possible explanation for the increased fall risk in rollator use could be that they interfere with the movement strategies of different tasks. Literature shows that individuals use different movement strategies, e.g., with or without upper body momentum, to stand up and sit down without a rollator [13, 15, 22, 23], but with no clear answer as to which movement strategy is the safest [24]. Furthermore, based on kinematic analyses, we have previously found that young individuals change their kinematic movement strategy when provided with rollator support, especially when the balance is challenged [25]. These changes in movement strategies indicate that rollators affect the underlying movement coordination patterns, which could be key to understanding human-rollator interactions and safe use.

With approximately 700 muscles and 300 mechanical degrees of freedom, the musculoskeletal system allows countless movement possibilities [26, 27]. To reduce the inherent complexity, the central nervous system (CNS) may employ a modular control architecture [26, 28, 29], for which synergies have been proposed as a possible representation [27, 30, 31]. Regardless of whether synergies are identified in spinal reflexes [32], as muscle synergies [33–35], or as kinematic synergies [36, 37], they may generate movement by activating a few functional groups rather than specifying each single element independently [38]. Muscle synergies are often used as a compact low-dimensional representation of a set of recorded muscle activity [27]. Through flexible recruitment and combinations of synergies, the CNS can generate an extensive movement repertoire [33]. According to a hierarchical organization, movement is generated through a combination of a preexisting, trial-independent part and a flexible, trial-dependent part [39]. The trial-independent part is reused across movements and presumably stored in subcortical areas of the CNS; whereas the trial-dependent part, which accounts for variations across trials, is presumably under cortical control [33, 40]. Different models of muscle synergies have been proposed and differ in terms of which part of the decomposition is trial-dependent and which is trial-independent [41–43]. In spatial muscle synergies, also called time-invariant or synchronous synergies, the trial-independent part is made up of vectors. These vectors, the weightings, represent the activations of multiple muscles, relative in magnitude to each other. These fixed vectors are combined with trial-dependent activation profiles (or time-varying coefficients), representing the amount and timing of the muscle weightings. In contrast, temporal muscle synergies (or temporal components, basic patterns), consist of muscle activation profiles, invariant over muscles and conditions, and trial-dependent muscle weightings. Hence, the relative weightings of muscle activations vary across trials. Another difference between these two models is the compactness of the muscle activity representation. In some studies, temporal muscle synergies provided a more compact representation than spatial synergies [42, 44, 45], but this is not always the case [45, 46]. Regardless of the model, muscle synergies are robust against the highly variable and stochastic nature of EMG patterns. Since they therefore reveal the neural organization underlying behavior and functional outcomes of muscular activation [47–49], they have been used in various settings to investigate questions regarding movement coordination [50], as well as in sit-to-stand movements.

Three to four spatial muscle synergies typically explain 87–94% of the variance in unassisted sit-to-stand movements across various age groups [51–56]. Usually, each synergy represents one biomechanical function, such as momentum transfer and postural stabilization. While muscle synergy structure seems robust in unassisted standing up, even with visual or vestibular disturbances, the activation timing changes with these disturbances [57]. Furthermore, synergy activation timings have been found to differ across movement strategies [56]. Consequently, analyzing the temporal structure is crucial. In contrast to unassisted standing up, assistive devices seem to impact not only the activation timing in standing up, but also the number of synergies increased when participants stood up while being pushed up by the chair [58], and studies with Nordic walking sticks [59] or exoskeletons [56, 57, 60, 61] show that assistive tools alter movement coordination; such that, for example, synergies specific to the device emerge. Consequently, when studying rollator-assisted standing up, it is essential to consider upper body involvement to account for the involvement of the arms. This is particularly relevant as the aging process leads to muscle weakening [59, 62], and individuals thus often need to push on armrests to master transitioning between sitting and standing. Consequently, it remains an open question how rollator usage influences full-body movement coordination in standing up movements, especially considering the different movement strategies and upper body involvement.

Like in standing up, assistive devices may alter sitting down coordination. Only a few biomechanical and movement coordination studies have analyzed sitting down [52, 61, 63], let alone with a rollator. This is surprising as sitting down is also a complex movement and not simply the opposite of standing up [15, 64]. For example, the gluteus maximus works concentrically when standing up but eccentrically when sitting down [61]. Also, upper body muscles may act differently when sitting down using a rollator, such that the arm extensor muscles presumably work eccentrically rather than concentrically. Accordingly, this implies that rollator usage may influence stand-to-sit movement coordination in a different manner from sit-to-stand.

To improve our understanding of the understudied human-rollator interactions and safe use in transitions between sitting and standing, it is necessary: (1) to develop a protocol and a methodology for assessing coordination during these understudied movements and (2) to establish baseline values in healthy cohorts. Therefore, we apply muscle synergy analysis and examine the movement coordination underlying different movement strategies for standing up and sitting down. We first examine which muscle synergy model is most appropriate, as it is not a priori clear which model best represents the sit-to-stand and stand-to-sit movements [41, 44, 45]. Then, we explore how different rollator support conditions (no assistance, light touch with haptic cues through the rollator handles, and full support with supposed lower limb load reduction) affect muscle synergies. As thorough studies on movement coordination underlying rollator-assisted movement are scarce [9], and the rollator-prone population is heterogeneous regarding their underlying deficits, this study investigates young participants as a baseline measure, with potentially limited loss of generalizability, even though Hanawa et al. [53] found that the synergies in standing up remain similar regardless of the participant’s age.

Still, to make standing up and sitting down more challenging, and as proprioceptive signals from the leg muscles are the primary source for postural control [65], we placed balance pads underneath their feet to evaluate the effect of rollator support while experiencing increased postural instability. Postural instability is common in many neurodegenerative diseases and movement disorders [66] and often leads to the prescription of rollators to improve postural stability [4, 9].

We hypothesized that (1) temporal muscle synergies represent sit-to-stand and stand-to-sit EMG patterns with a different compactness than spatial muscle synergies. Furthermore, we hypothesize that (2) the muscle synergy activation differs across movement strategies and that (3) rollator support influences the weightings between upper body and lower limb muscles for both standing up and sitting down.

## Materials & methods

Our previously published articles on this dataset describe our analysis of the kinematic and kinetic data [20, 25]. Here, we introduce all the steps regarding the EMG analysis.

### Participants

**Fig. 1:**
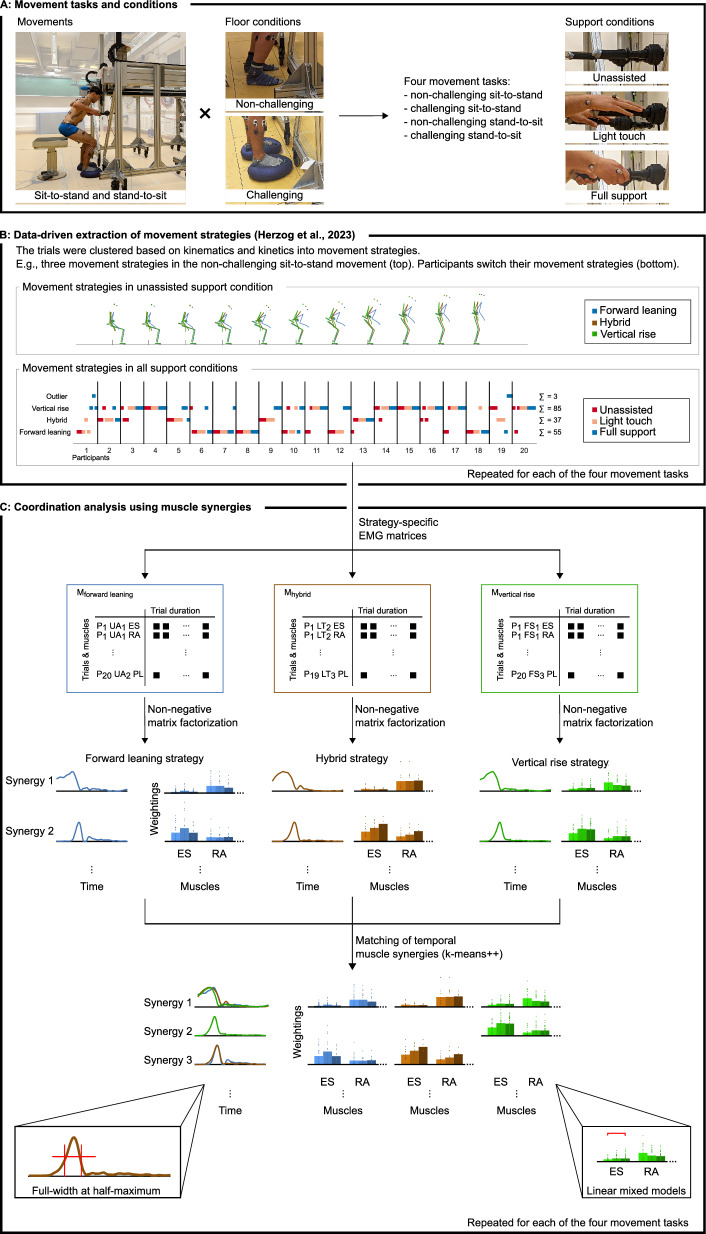
Experimental setup and data analysis. **A** The participant stands up from an instrumented chair with the custom-made robot rollator simulator. Full-body passive markers for motion tracking and EMG electrodes were placed on the body. Two movements were studied: sit-to-stand and stand-to-sit. Two floor conditions were used (middle): non-challenging (lab floor) and challenging (balance pads). Three different support conditions were used (right): unassisted (handles not used), light touch (palm on the handles), and full support (power grip). The figure is adapted from [25]. **B** Participants used different movement strategies and switched between them, as exemplarily shown for the non-challenging sit-to-stand task. The bottom plot shows the distribution of the trials among strategies. One dot represents one trial. The row indicates to which movement strategy it belongs. The column shows to which participant it belongs. The support conditions are color-coded as indicated by the legend. The labels on the right y-axis show how many trials were associated with the strategy written on the left y-axis. The figure is adapted from [25]. **C** EMG data from the trials of the same movement strategy were arranged into a matrix (for example, M_vertical rise_). Temporal muscle synergies were extracted from each matrix with NMF [70–72], resulting in trial-independent activation profiles and trial-dependent muscle weightings. The activation profiles were matched across the movement strategies and ordered chronologically using k-means++ clustering, ensuring a correlation coefficient above 0.9. Bottom: The duration of each activation profile was assessed as full-width at half-maximum. Linear mixed models were used to investigate how rollator support affects the muscle weightings. *P*_1_ participant 1, *UA*_1_ first unassisted trial, *LT*_1_ first light touch trial, *FS*_1_ first full support trial, *ES* M. erector spinae, *RA* M. rectus abdominis, *NMF* non-negative matrix factorization [70, 71]

Twenty young and healthy volunteers (10 women, 10 men; 25.5 ± 3.8 years, 1.71 ± 0.08 m height, 67.6 ± 10.9 kg mass) gave written informed consent and participated in the study. The participant shown in Fig. 1 gave informed consent to publish the image. The Ethics Committee of the Medical Department of Heidelberg University (S-105/2021) approved the study, which was then performed according to the Declaration of Helsinki.

### Experimental protocol

The participants sat still and, after hearing “stand up” by the experimenter, they stood up at their own pace. Then, the experimenter said, “stand still”. After standing still for at least two seconds, the experimenter said, “sit down,” and the participants sat down at their own pace. The seat height was set to the height of the participant’s lateral epicondyle of the femur, and a custom-built robot rollator simulator was used to provide rollator support (Fig. 1). Following recommendations in the health care literature [67–69], the handle height was set at the participant’s standing wrist height. According to the support condition, they did not use the rollator handles at all (unassisted, UA), only with a light touch of the hand, i.e., by placing the hand with a palm grip onto the handle to receive a haptic cue (light touch, LT), or with a power grip (full support, FS). These support conditions were combined with two floor conditions: the standard lab floor and a more “challenging ground”, which was created by positioning a circular rubber balance pad with a compliant surface (Dynair^®^ Ballkissen^®^, diameter 33 cm, height 8 cm, TOGU GmbH, Prien-Bachham, Germany) under each foot. Participants familiarized themselves with the task by performing two repetitions in each condition combination (support: unassisted, light touch, full support; floor: non-challenging, challenging). No further instructions on the movement execution were given, allowing the participants to stand up and sit down as naturally as possible. All participants performed three valid, non-consecutive repetitions in each condition combination, resulting in a total of 18 trials per participant. The order of the support and floor conditions was randomized across participants.

### Data collection

Full-body 3D kinematics were obtained using the IOR full-body marker model [73, 74] and ten cameras (150 Hz; Type 5 + , Qualisys, Gothenburg, Sweden). Ground reaction forces (GRF; 1,000 Hz; Bertec Corp., Columbus, OH, USA) and forces on the seating surface (142 Hz; Phidgets Inc., Calgary, AB, Canada) were measured.

Thirty surface EMG electrodes (two systems, 1,500 and 4,000 Hz; Noraxon USA, Scottsdale, AZ, USA) captured full-body muscle activity of the following muscles bilaterally: pectoralis major (Pec), latissimus dorsi (Lat), trapezius (Tra), deltoideus (Del), biceps brachii (Bic), triceps brachii (Tri), gluteus medius (GM), tensor fasciae latae (TF), rectus femoris (RF), vastus medialis (VM), biceps femoris (BF), tibialis anterior (TA), peroneus longus (PL), and gastrocnemius (GA). Additionally, erector spinae (ES) and rectus abdominis (RA) activities were recorded. The participants’ skin was prepared by shaving, abrasion, and cleansing with alcohol to ensure good electrode–skin contact before the Ag/AgCl electrodes were attached according to SENIAM guidelines [75] and [76].

### Data processing

To reconstruct the 3D coordinates of the markers, raw kinematic data were processed offline with Qualisys Track Manager (v2018.1). Subsequently, force and kinematic data were filtered with a 4th-order zero-lag low-pass Butterworth filter at 10 Hz. Using Visual3D (v6, C-Motion Inc., Germantown, MD, USA), full-body kinematics and the center of mass (CoM) were then calculated. Further data analyses were done in Matlab (R2023b, Natick, MA, USA). Raw EMG data were bandpass (20–500 Hz) and notch (50 Hz) filtered with a 4th-order zero-lag Butterworth filter [53]. ECG artifacts apparent in the trunk muscle recordings were removed with a template-matching procedure [77]. For robustness, we created a muscle- and participant-specific template of ECG artifacts using the data from the sit-to-stand and stand-to-sit recordings and an additional 9 min of still-standing recordings. Subsequently, filtered EMG data were full-wave rectified and smoothed with a 4th-order zero-lag low-pass Butterworth filter at 10 Hz [53]. Movement start, seat-off/on, and movement end were identified using a k-means++ algorithm on the GRF and CoM data [78]. As muscles need to be active before a visible movement starts, data from 200 ms before the detected start of the movement were included [79, 80]. Afterward, data were segmented and time-normalized to 101 time points (100%) using a spline interpolation. Finally, EMG data were amplitude-normalized per muscle and participant to their maximum activity across the 18 trials [71]. Of the 720 trials, 24 were not included in the analyses as some recordings of a few muscles were corrupt (Supplementary Table 1, Additional file 1).

### Movement strategies

Our previous investigation found that participants switched movement strategies when introduced to a rollator [25]. In particular, three movement strategies were identified for the sit-to-stand non-challenging task (“forward leaning”, “hybrid”, and “vertical rise”) and two for the challenging task (“exaggerated forward leaning” and “forward leaning”). Likewise, three and two movement strategies were identified respectively in the stand-to-sit tasks (“vertical lowering”, “hybrid”, and “backward lowering”; and “exaggerated forward leaning” and “forward leaning”). The naming of these strategies was inferred by visual inspection of their movement progression, the different hip, knee, and ankle sagittal angle courses, and the relative movements between the CoM and the heel. The grouping of trials into the movement strategies was used in the current analysis to extract muscle synergies specific to the movement strategies. Supplementary Fig. 1, Additional file 1 shows the distribution of trials among strategies.

In short, the forward leaning strategy in the sit-to-stand movement showed more hip flexion and less overlapping anterior and vertical CoM movement than the vertical rise strategy. The hybrid strategy showed kinematic and kinetic time courses sometimes more aligned with one than the other strategy. In the challenging condition, the exaggerated forward leaning strategy was characterized by a wide upper body forward lean and an earlier movement of the CoM over the BoS than the forward leaning strategy. In the stand-to-sit movement, the backward lowering strategy revealed smaller hip, knee, and ankle angles than the other two, and the vertical lowering strategy showed a vertical orientation of the trunk. In the challenging condition, like with the sit-to-stand movement, the exaggerated forward leaning strategy was characterized by a wide upper body forward lean and showed less overlapping vertical and posterior CoM movement than the forward leaning strategy.

### Data analysis

#### Muscle synergy analysis

We extracted muscle synergies with respect to our previous findings that participants switched their movement strategies when introduced to a rollator ([25]; Fig. 1). As stated in the introduction, the two models (spatial and temporal muscle synergies) describe different aspects of movement coordination [43–46, 81]. Temporal muscle synergies allow us to follow the underlying assumption that the CNS uses a fixed temporal sequence (trial-independent activation profiles) for the different movement strategies and that muscle weightings vary (trial-dependent muscle activation vectors) across the support conditions. Spatial and temporal muscle synergies are commonly extracted using non-negative matrix factorization (NMF) but with differently arranged EMG input matrices [43–46, 81].

For the extraction of temporal muscle synergies, the EMG signals (30 channels) of all trials (tr is the number of trials) belonging to the same strategy (strat) were horizontally concatenated into a data matrix ${\mathbf{M}}_{\mathbf{s}\mathbf{t}\mathbf{r}\mathbf{a}\mathbf{t}}\in {\mathbb{R}}_{\ge 0}^{101 \times 30\cdot \text{tr}}$. NMF decomposed **M**_**strat**_ into a set of N_strat_ trial-independent activation profiles ${\text{C}}_{\text{strat},\text{ n}}\in {\mathbb{R}}_{\ge 0}^{101 \times 1}$, and trial-dependent muscle weightings ${\mathbf{W}}_{\mathbf{s}\mathbf{t}\mathbf{r}\mathbf{a}\mathbf{t},~\mathbf{n}}^{\mathbf{s}}\in {\mathbb{R}}_{\ge 0}^{1 \times 30}$ [70–72]. Thus, EMG data from a single trial (s is the trial index) ${\mathbf{M}}_{\mathbf{s}\mathbf{t}\mathbf{r}\mathbf{a}\mathbf{t}}^{\mathbf{s}}\left(\text{t}\right)$ were decomposed with:

$${\mathbf{M}}_{\mathbf{s}\mathbf{t}\mathbf{r}\mathbf{a}\mathbf{t}}^{\mathbf{s}}\left(\text{t}\right)\approx {\sum }_{\text{n}\in {\text{N}}_{\text{strat}}}{\text{C}}_{\text{strat},\text{ n}}\left(\text{t}\right) \cdot {\mathbf{W}}_{\mathbf{s}\mathbf{t}\mathbf{r}\mathbf{a}\mathbf{t},~\mathbf{n}}^{\mathbf{s}}$$

To investigate hypothesis 1 that temporal muscle synergies represent sit-to-stand and stand-to-sit EMG patterns with a different compactness than spatial muscle synergies, we also extracted spatial muscle synergies. Therefore, the EMG signals (30 channels) of all trials belonging to the same strategy (strat) were horizontally concatenated into a data matrix ${\mathbf{M}}_{\mathbf{s}\mathbf{t}\mathbf{r}\mathbf{a}\mathbf{t},~\mathbf{s}\mathbf{p}\mathbf{a}\mathbf{t}\mathbf{i}\mathbf{a}\mathbf{l}}\in {\mathbb{R}}_{\ge 0}^{30 \times 101\cdot \text{tr}}$. NMF decomposed **M**_**strat, spatial**_ into a set of N_strat, spatial_ trial-independent muscle weightings ${\mathbf{W}}_{\mathbf{s}\mathbf{t}\mathbf{r}\mathbf{a}\mathbf{t},~\mathbf{n},~\mathbf{s}\mathbf{p}\mathbf{a}\mathbf{t}\mathbf{i}\mathbf{a}\mathbf{l}}\in {\mathbb{R}}_{\ge 0}^{30 \times 1}$, and trial-dependent activation profiles ${\text{C}}_{\text{strat},\text{ n},\text{ spatial}}^{\text{s}}\in {\mathbb{R}}_{\ge 0}^{1 \times 101}$ [70–72]. Thus, EMG data from a single trial (s is the trial index) ${\mathbf{M}}_{\mathbf{s}\mathbf{t}\mathbf{r}\mathbf{a}\mathbf{t},~\mathbf{s}\mathbf{p}\mathbf{a}\mathbf{t}\mathbf{i}\mathbf{a}\mathbf{l}}^{\mathbf{s}}\left(\text{t}\right)$ were decomposed with:

$${\mathbf{M}}_{\mathbf{s}\mathbf{t}\mathbf{r}\mathbf{a}\mathbf{t},~\mathbf{s}\mathbf{p}\mathbf{a}\mathbf{t}\mathbf{i}\mathbf{a}\mathbf{l}}^{\mathbf{s}}\left(\text{t}\right)\approx {\sum }_{\text{n}\in {\text{N}}_{\text{strat},\text{ spatial}}}{\mathbf{W}}_{\mathbf{s}\mathbf{t}\mathbf{r}\mathbf{a}\mathbf{t},~\mathbf{n},~\mathbf{s}\mathbf{p}\mathbf{a}\mathbf{t}\mathbf{i}\mathbf{a}\mathbf{l}}\cdot {\text{C}}_{\text{strat},\text{ n},\text{ spatial}}^{\text{s}}(\text{t})$$

NMF’s iterative decomposition was limited to 3,000 iterations and started 50 times to avoid convergence to a local minimum [52, 82].

A fivefold cross-validation procedure was used to increase the confidence that the extracted muscle synergies were robust and generalizable rather than due to characteristics of single trials. In line with the literature, a training/test split of 80:20 was employed [33, 83]. Muscle synergies were extracted from a random 80% portion of the trials. Then, the trial-independent parts were fixed and fitted to the remaining 20% of the trials. Finally, the reconstruction quality R^2^_CV_ of the fits to the test sets was used to identify the number of synergies. R^2^ is a multivariate measure allowing assessment of the reconstruction quality: R^2^ = 1 − SSE/SST, with SSE being the sum of the squared residuals and SST the sum of the squared residuals from the mean vector [84]. The numbers of synergies N_strat_ and N_strat, spatial_ were chosen at the R^2^-knee point, after which the R^2^ curve remained approximately straight [84]. Therefore, a series of linear regressions were fitted to the R^2^ curve, starting with the interval [N_1_, N_30_] and iteratively removing the smallest N from the interval. Then, the regressions’ mean squared residual errors (MSE) were calculated, and N_strat_ and N_strat, spatial_ were selected for the first number N with an MSE smaller than 10^–4^. The number of synergies to extract must be chosen carefully to obtain a good low-dimensional representation of the data with minimum noise [85, 86], and numerous criteria have been proposed [87]. To avoid the results being specific to the choice of N_strat_, rather than reflecting physiological patterns, an additional criterion was used to compare the results. Therefore, N_strat_^*^ was chosen to be the minimum number fulfilling both a global (${R}_{{N}_{strat}}^{2}\ge 0.9$) and a local criterion (${R}_{n}^{2}-{R}_{n-1}^{2}<0.05; n=1...{N}_{strat}$).

To investigate hypothesis 1 that temporal muscle synergies represent sit-to-stand and stand-to-sit EMG patterns with a different compactness than spatial muscle synergies, we compared the compactness of the spatial and temporal muscle synergy extractions using two metrics. Firstly, we compared the dimensionality reduction (R^2^-knee criterion) between temporal and spatial extractions (N_strat_ and N_strat, spatial_). Secondly, the number of trial-dependent parameters (temporal: synergies ∙ muscles, spatial: time samples ∙ synergies) and the number of trial-independent parameters (temporal: time samples ∙ synergies, spatial: synergies ∙ muscles) were also compared between the temporal and spatial extraction, with the number of synergies selected at the respective R^2^-knee point [42].

This justified the choice of temporal muscle synergies beyond the assumption that the CNS uses a fixed temporal sequence (trial-independent activation profiles) for the different movement strategies and that muscle weightings vary (trial-dependent muscle activation vectors) across the support conditions (degree of handle support). All steps regarding muscle synergy analyses were done separately for the two movements and floor conditions.

#### Matching of similar synergies across the movement strategies

To investigate hypothesis 2 that muscle synergy activation differs across movement strategies, the activation profiles of the different movement strategies were matched with k-means++ clustering (Matlab kmeans, with the ‘plus’ option, 50 restarts with random initial cluster centroid positions, maximum 1,000 iterations; Fig. 1; [88]). Suppose two movement strategies show n temporal synergies each. If the two movement strategies do not differ in terms of their activation profiles, k-means++ groups them into n clusters, each with two activation profiles, one from the first and one from the second movement strategy. A difference between the movement strategies is identified if there are more than n clusters. In this case, there are clusters with only one synergy. These clusters are specific to one of the two movement strategies. Accordingly, the activation profiles within the same cluster are characterized by a similar shape of activation and timing.

The number of clusters was increased from one until the minimum number for which (1) a correlation coefficient [88] of at least 0.9 per match of all activations profiles within each cluster with their centroid was ensured and (2) only one activation profile per strategy was included within each cluster. The clustering was repeated ten times to confirm the robustness of the cluster assignments [89].

#### Statistics

##### Timing and duration of temporal synergies

To investigate hypothesis 2 that muscle synergy activation differs across movement strategies beyond their similarity (see 3.6.2), we assessed the duration of activation by measuring the full-width at half-maximum of the main peak (FWHM; Matlab findpeaks; [88]). Therefore, the time difference between the two points at half-height on either side of the main peak was calculated. As sit-to-stand and stand-to-sit are sequential movements, we cannot assume that the boundary synergies (synergies with the peaks close to the movement start and end) are symmetrical. In the case of boundary synergies, we measured the difference between the movement start and the point of half-height of the descending synergy or the difference between the point of half-height and the movement end.

##### Linear mixed model to assess differences in the muscle weightings

To investigate hypothesis 3, i.e., to identify the changes in the muscle weightings between the support conditions (unassisted, light touch, full support), a linear mixed model (LMM) was used (Matlab fitlme). The LMM considers that repeated measures of a single participant are likely correlated [90, 91], allowing us to manage the distribution of participants’ trials over several movement strategies. To account for the simultaneous changes in multiple muscle weightings (trial-dependent muscle activation vectors) with the support condition, a multivariate LMM approach was used [92].

Single trials (level 1) were nested into participants (level 2). The support conditions were included as dummy variables using reference coding (LT or FS set to 1, or both to 0 for the unassisted condition). The first multivariate LMM was calculated with UA as the reference group, and the second multivariate LMM with LT as the reference group, allowing investigation of all pairwise comparisons within the support conditions. Each vector of muscle weightings belongs to one temporal synergy, i.e., the reference frames in which the muscle weightings lie are different across the temporal synergies, so one multivariate LMM was used for each vector. For example, for the non-challenging sit-to-stand task using the forward leaning strategy, seven synergies led to 14 multivariate LMM tests within this task (seven synergies tested once with UA and once with LT as reference group). Sex was added as a within-participant control variable at level 1 but was not included in the final model as it did not improve the model based on the change in the -2 log-likelihood or the Akaike's information criterion (Matlab’s linearmixedmodel.compare function; [93, 94]). The residual plots were inspected to assess normality, linearity, and homoscedasticity as prerequisites for LMM, and no gross violations were found [95, 96].

The multivariate LMM regression formula was:

Level 1﻿:

$${\text{Weightings}}_{\text{tp}}={\upbeta }_{0\text{p}}+{\upbeta }_{1\text{p}}{\text{LT}}_{\text{tp}}+{\upbeta }_{2\text{p}}{\text{FS}}_{\text{tp}}+{\upepsilon }_{\text{tp}}$$

Level 2:

$$\begin{aligned}{\upbeta }_{0\text{p}}&={\upgamma }_{00}+{\text{u}}_{0\text{p }}\hfill \\{\upbeta }_{1\text{p}}&={\upgamma }_{10}\hfill \\{\upbeta }_{2\text{p}}&={\upgamma }_{20}\end{aligned}$$

${\text{Weightings}}_{\text{tp}}$ represents the mean muscle weighting across the left and right limb of each bilaterally assessed muscle and the single weightings of M. erector spinae and M. rectus abdominis of a given synergy on the t^th^ trial for the p^th^ participant. The ${\upbeta }_{0\text{p}}$ represents the intercept, ${\upbeta }_{1\text{p}}$ and ${\upbeta }_{2\text{p}}$ the fixed effects for the support conditions, and ${\upepsilon }_{\text{tp}}$ represents the trial- and participant-specific residual. The variable ${\text{u}}_{0\text{p}}$ is a support-specific random component of ${\upbeta }_{0\text{p}}$ and the $\upgamma$ fixed effect parameters. Accordingly, the following formula was used for the function specification of the fitlme function: ‘Weightings ~ LT + FS + (1 | Participant)’. To account for the multivariate nature, muscle weightings were vertically concatenated into ${\text{Weightings}}_{\text{tp}}$. For the second multivariate LMM test, ‘LT’ was exchanged with ’UA’ to have LT as the reference group. The t-statistic on the ß coefficients was used for hypothesis testing with the significance set a priori at a two-sided α = 0.05 [94]. To reduce the probability of type I errors, the level of significance was adjusted according to the number of tests within one movement strategy (e.g., for the seven synergies in the non-challenging, sit-to-stand forward leaning strategy, the level of significance was adjusted for 14 tests with Bonferroni correction).

If the multivariate LMM showed significance regarding the support groups [92], **MscName** (a categorical variable representing the muscle names, using effect coding, **MscName** indicates the vector of dummy variables) was used in the second step as a dummy variable of the LMM analyses using effect coding. Therefore, the interaction between the muscles and the support condition was added to the LMM. This revealed the muscles in which the support condition affects their weighting with respect to the group mean of the reference muscle (M. Rectus abdominis) and support group (effect coding). Consequently, the following model was used:

Level 1:

$${\text{Weightings}}_{\text{tp}}={\upbeta }_{0\text{p}}+{\upbeta }_{1\text{p}}{\text{LT}}_{\text{tp}}+{\upbeta }_{2\text{p}}{\text{FS}}_{\text{tp}}+{{\varvec{\upbeta}}}_{3\mathbf{p}}^{\mathbf{T}}{\mathbf{M}\mathbf{s}\mathbf{c}\mathbf{N}\mathbf{a}\mathbf{m}\mathbf{e}}_{\mathbf{p}}+ {{\varvec{\upbeta}}}_{4\mathbf{p}}^{\mathbf{T}}{\text{LT}}_{\text{tp}}{\mathbf{M}\mathbf{s}\mathbf{c}\mathbf{N}\mathbf{a}\mathbf{m}\mathbf{e}}_{\mathbf{p}}+{{\varvec{\upbeta}}}_{5\mathbf{p}}^{\mathbf{T}}{\text{FS}}_{\text{tp}}{\mathbf{M}\mathbf{s}\mathbf{c}\mathbf{N}\mathbf{a}\mathbf{m}\mathbf{e}}_{\mathbf{p}}+ {\upepsilon }_{\text{tp}}$$

Level 2:

$$\begin{aligned} {\upbeta }_{{0{\text{p}}}} &= {\upgamma }_{00} + {\text{u}}_{{0{\text{p}}}} \hfill \\ {\upbeta }_{{1{\text{p}}}} &= {\upgamma }_{10} \hfill \\ {\upbeta }_{{2{\text{p}}}} &= {\upgamma }_{20} \hfill \\ {{\varvec{\upbeta}}}_{{3{\mathbf{p}}}} &= {{\varvec{\upgamma}}}_{30} \hfill \\ {{\varvec{\upbeta}}}_{{4{\mathbf{p}}}} &= {{\varvec{\upgamma}}}_{40} \hfill \\ {{\varvec{\upbeta}}}_{{5{\mathbf{p}}}} &= {{\varvec{\upgamma}}}_{50} \hfill \\ \end{aligned}$$

Matlab model function: ‘Weightings ~ (LT + FS) * MscName + (1 | Participant)’.

To reduce the probability of type I errors, the significance level (α = 0.05) was adjusted to the number of tests with the second model, at maximum three if a significant effect was found for each pairwise comparison of UA, LT, and FS. The LMM was implemented using the maximum likelihood method.

## Results

### Temporal muscle synergies result in a more compact representation than spatial muscle synergies in sit-to-stand and stand-to-sit EMG patterns

**Fig. 2:**
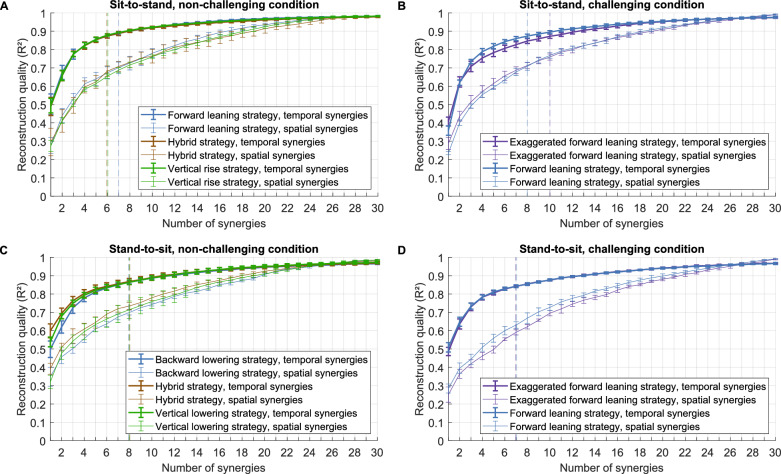
Reconstruction quality (R^2^) for every movement (**A**–**D**) and movement strategy (color-coded). Thick lines: Temporal muscle synergy reconstruction quality, thin lines: spatial muscle synergy reconstruction quality. The figure shows means and standard deviations over the fivefold cross-validation runs. The vertical dotted lines, color-coded with respect to the movement strategies, indicate the choice for the number of synergies according to Table 1

**Fig. 3:**
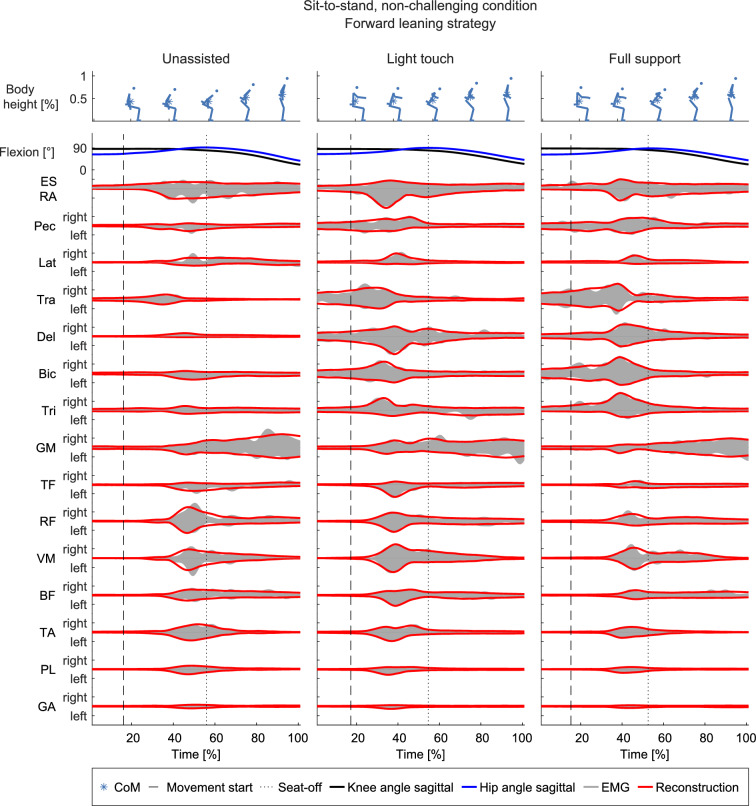
Kinematics (top), original EMG (gray areas), and reconstruction (red solid lines). Data from one exemplary participant using the forward leaning strategy to stand up. The dashed line indicates the movement start, i.e., the first visible CoM movement. The dotted line indicates seat-off. Left: unassisted, middle: light touch, right: full support condition. Muscle activity of the right limb is in the positive direction, and the left limb is in the negative direction

To investigate if temporal muscle synergies represent sit-to-stand and stand-to-sit EMG patterns with different compactness than spatial muscle synergies, both types of synergy were extracted from the EMG data. In each movement task, six to ten temporal synergies reconstructed the EMG patterns (Fig. 2) with an R^2^ between 0.84 and 0.89 (Fig. 2, Table 1). In particular, 5.8 ± 2.4 fewer synergies were necessary for the temporal than for the spatial extraction with the R^2^-knee criterion. Figure 3 shows the EMG and the good reconstruction for an exemplary participant. Furthermore, Fig. 3 also shows that muscle activity and their reconstruction are very symmetrical between the left and right sides, along with an increase in upper body muscle activity with increasing support. The number of trial-independent parts is smaller for the temporal muscle synergies than for the spatial muscle synergies, and fewer trial-dependent parts are necessary for every trial (Table 2). Accordingly, the total number of the trial-independent and trial-dependent parts is smaller for the temporal muscle synergies, which indicates that the higher R^2^ of the temporal muscle synergies is not simply due to the number of parts. Thus, temporal muscle synergies represent the EMG patterns more compactly than spatial muscle synergies (hypothesis 1). Accordingly, the subsequent analyses were done with temporal muscle synergies.

**Table 1:** The numbers and R^2^ of synergies according to the applied criterion and choice of model

Movement	Sit-to-stand, non-challenging			Sit-to-stand, challenging	
Strategy	Forward leaning	Hybrid	Vertical rise	Exaggerated forward leaning	Forward leaning
Temporal	7	6	6	10	8
R^2^-knee (N_strat_)	0.888 ± 0.008	0.874 ± 0.012	0.879 ± 0.005	0.873 ± 0.012	0.874 ± 0.011
Temporal	8	8	8	13	11
R^2^ > 0.9 (N_strat_^*^)	0.902 ± 0.005	0.902 ± 0.009	0.905 ± 0.003	0.905 ± 0.009	0.906 ± 0.009
Spatial	15	12	16	11	13
R^2^-knee (N_strat, spatial_)	0.952 ± 0.005	0.931 ± 0.007	0.953 ± 0.002	0.882 ± 0.010	0.920 ± 0.006

Movement	Stand-to-sit, non-challenging			Stand-to-sit, challenging	
Strategy	Backward lowering	Hybrid	Vertical lowering	Exaggerated forward leaning	Forward leaning
Temporal	8	8	8	7	7
R^2^-knee (N_strat_)	0.865 ± 0.009	0.868 ± 0.017	0.864 ± 0.013	0.842 ± 0.004	0.842 ± 0.007
Temporal	12	11	12	13	13
R^2^ > 0.9 (N_strat_^*^)	0.902 ± 0.004	0.901 ± 0.014	0.905 ± 0.013	0.902 ± 0.005	0.901 ± 0.005
Spatial	16	13	13	12	12
R^2^-knee (N_strat, spatial_)	0.929 ± 0.005	0.915 ± 0.013	0.914 ± 0.011	0.895 ± 0.005	0.896 ± 0.004

R^2^ values are mean and standard deviation over the fivefold cross-validation runs

**Table 2:** Amount of dimensionality reduction in terms of trial-independent and trial-dependent signals according to [42]

Movement		Sit-to-stand, non-challenging			Sit-to-stand, challenging	
Strategy		Forward leaning	Hybrid	Vertical rise	Exaggerated forward leaning	Forward leaning
Number of trial-independent parameters	Temporal: time samples ∙ synergies	707	606	606	1010	808
	Spatial: synergies ∙ muscles	450	360	480	330	390
Number of trial-dependent parameters	Temporal: synergies ∙ muscles	210	180	180	300	240
	Spatial: time samples ∙ synergies	1515	1212	1616	1111	1313
Sum temporal		917	786	786	1310	1048
Sum spatial		1965	1572	2096	1441	1703

Movement		Stand-to-sit, non-challenging			Stand-to-sit, challenging	
Strategy		Vertical lowering	Hybrid	Backward lowering	Exaggerated Forward leaning	Forward leaning
Number of trial-independent parameters	Temporal: time samples ∙ synergies	808	808	808	707	707
	Spatial: synergies ∙ muscles	480	390	390	360	360
Number of trial-dependent parameters	Temporal: synergies ∙ muscles	240	240	240	210	210
	Spatial: time samples ∙ synergies	1616	1313	1313	1212	1212
Sum temporal		1048	1048	1048	917	917
Sum spatial		2096	1703	1703	1572	1572

### The timing of temporal muscle synergies differs across movement strategies

To investigate hypothesis 2 that muscle synergy activation differs across movement strategies, temporal synergy activation profiles were clustered using k-means++ across the movement strategies and differences were identified if specific clusters were found (see 3.6.2).

#### Sit-to-stand movement strategies

**Fig. 4:**
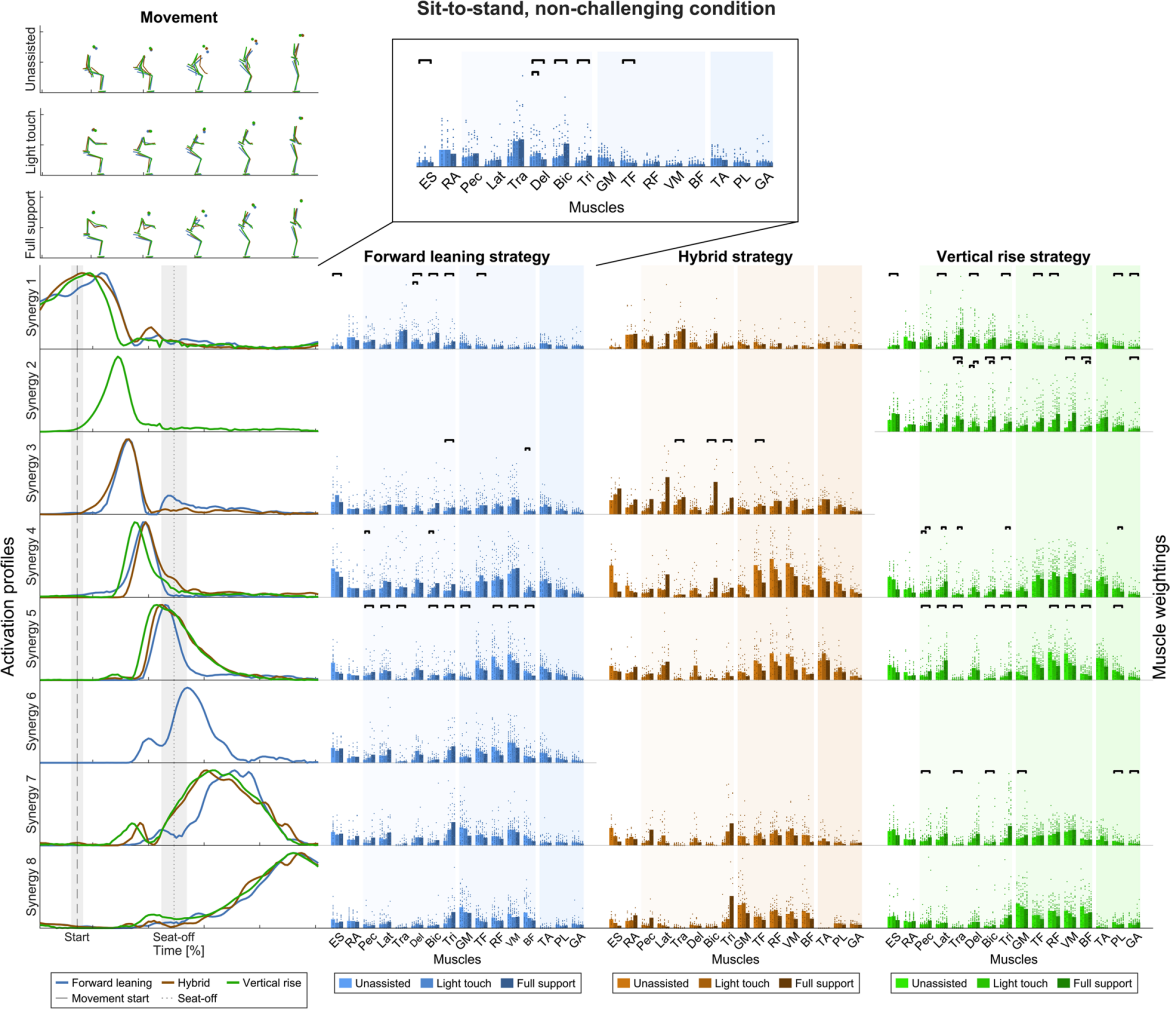
Temporal muscle synergies in the sit-to-stand movement and non-challenging condition. Top: Exemplary movements in the unassisted, light touch, and full support conditions (in rows) and movement strategies (color-coded). Bottom, left: Temporal muscle synergies sorted chronologically and color-coded according to the movement strategy. The dashed line indicates the movement start, i.e., the first identified CoM movement. The dotted line indicates seat-off. The timing of the three movement strategies was different, as indicated by the strategy-specific temporal synergies 2, 3, and 6. Bottom, right: Bar graphs show mean muscle weightings across the left and right side (except ES and RA), all trials within a strategy and support condition, and single dots represent trial-specific weightings in two columns for the left and right limbs. The different color shades indicate the support conditions. The bars indicate statistically significant differences according to the LMM statistics (details in Additional file 2)

In the non-challenging task (Fig. 4), the activation profiles of the seven forward leaning synergies and the six hybrid and six vertical rise strategies were grouped into eight clusters. Timing differed across the movement strategies between movement start and shortly before seat-off. Also, there is one distinct forward leaning synergy active right after seat-off. At movement start, seat-off, and at movement end, the clusters contained synergies from all three movement strategies. Hence, at these times, the activation profiles do not differ.

**Fig. 5:**
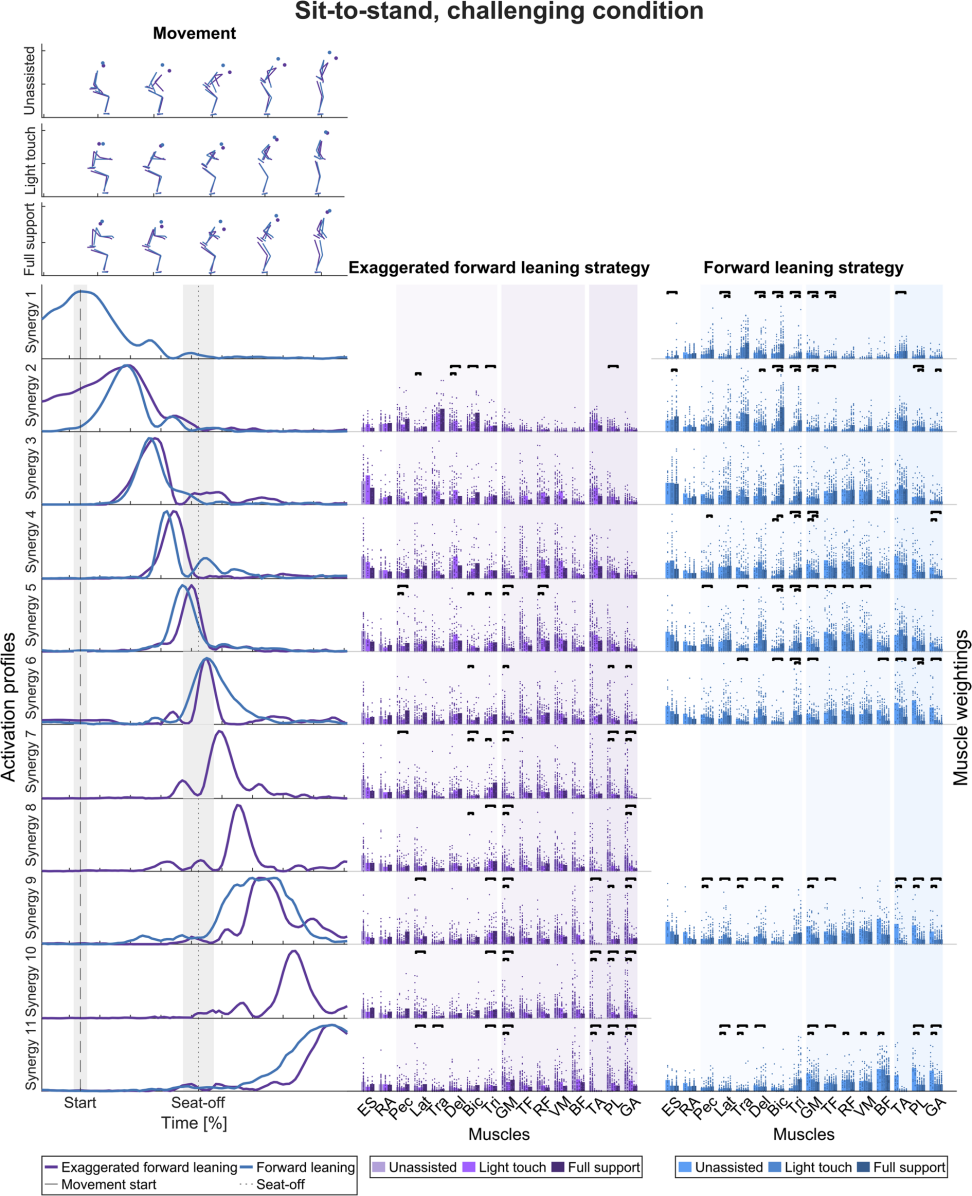
Temporal muscle synergies in the sit-to-stand movement and challenging condition. Top: Exemplary movements in the unassisted, light touch, and full support conditions (in rows) and movement strategies (color-coded). Bottom, left: Temporal muscle synergies sorted chronologically and color-coded according to the movement strategy. The dashed line indicates the movement start, i.e., the first identified CoM movement. The dotted line indicates seat-off. The timing of the two movement strategies was different, as indicated by the strategy-specific temporal synergies 1, 7, 8, and 10. Bottom, right: Bar graphs show mean muscle weightings across the left and right side (except ES and RA), all trials within a strategy and support condition, and single dots represent trial-specific weightings in two columns for the left and right limbs. The different color shades indicate the support conditions. The bars indicate statistically significant differences according to the LMM statistics (details in Additional file 2)

In the challenging condition (Fig. 5), the ten synergies of the exaggerated forward leaning and eight from the forward leaning strategy were grouped into eleven clusters. Right after seat-off, the timing of the synergies differs between the two movement strategies, with three distinct exaggerated forward leaning temporal synergies. This differs from the non-challenging task, where we additionally found differences between movement start and seat-off. Across all movement strategies, the main synergy peaks widths (FWHM) were larger at movement start and end than in the middle, especially around seat-off (Table 3).

**Table 3:** Full-width at half-maximum (FWHM) of the temporal synergies

Movement	Sit-to-stand, non-challenging			Sit-to-stand, challenging		Stand-to-sit, non-challenging			Stand-to-sit, challenging	
Strategy/synergy	Forward leaning	Hybrid	Vertical rise	Exaggerated forward leaning	Forward leaning	Vertical lowering	Hybrid	Backward lowering	Exaggerated forward leaning	Forward leaning
1	29.1	29.0	25.3		24.4	16.4	15.7	19.4	24.2	24.1
2			9.4	33.2	12.6	23.4	21.3	22.0	18.7	19.9
3	8.7	9.8		11.1	9.0	11.0	13.7	12.7	14.1	14.6
4	9.0	8.4	8.8	8.0	6.3			5.8	11.3	7.4
5	9.5	15.8	18.9	6.7	8.4	8.8	10.8	6.7		6.6
6	13.5			5.8	12.4	10.9	8.4	9.3	8.9	14.2
7	23.1	33.3	32.1	8.2			6.9		8.8	
8	22.8	27.3	25.0	7.1		10.2	10.8	11.4	30.0	30.9
9				12.0	25.5	15.1				
10				9.6		20.0	28.3	31.2		
11				13.1	20.5					

Trials are time-normalized to 101 points. Therefore, the numbers in the table represent fractions of the whole trial durations

The findings that the timing of the temporal muscle synergies differs across the movement strategies are robust regarding the criterion for selecting the numbers of synergies (Supplementary Fig. 2, Additional file 1). Therefore, we accept our second hypothesis that muscle synergy activation differs across movement strategies regarding sit-to-stand movements.

#### Stand-to-sit movement strategies

**Fig. 6:**
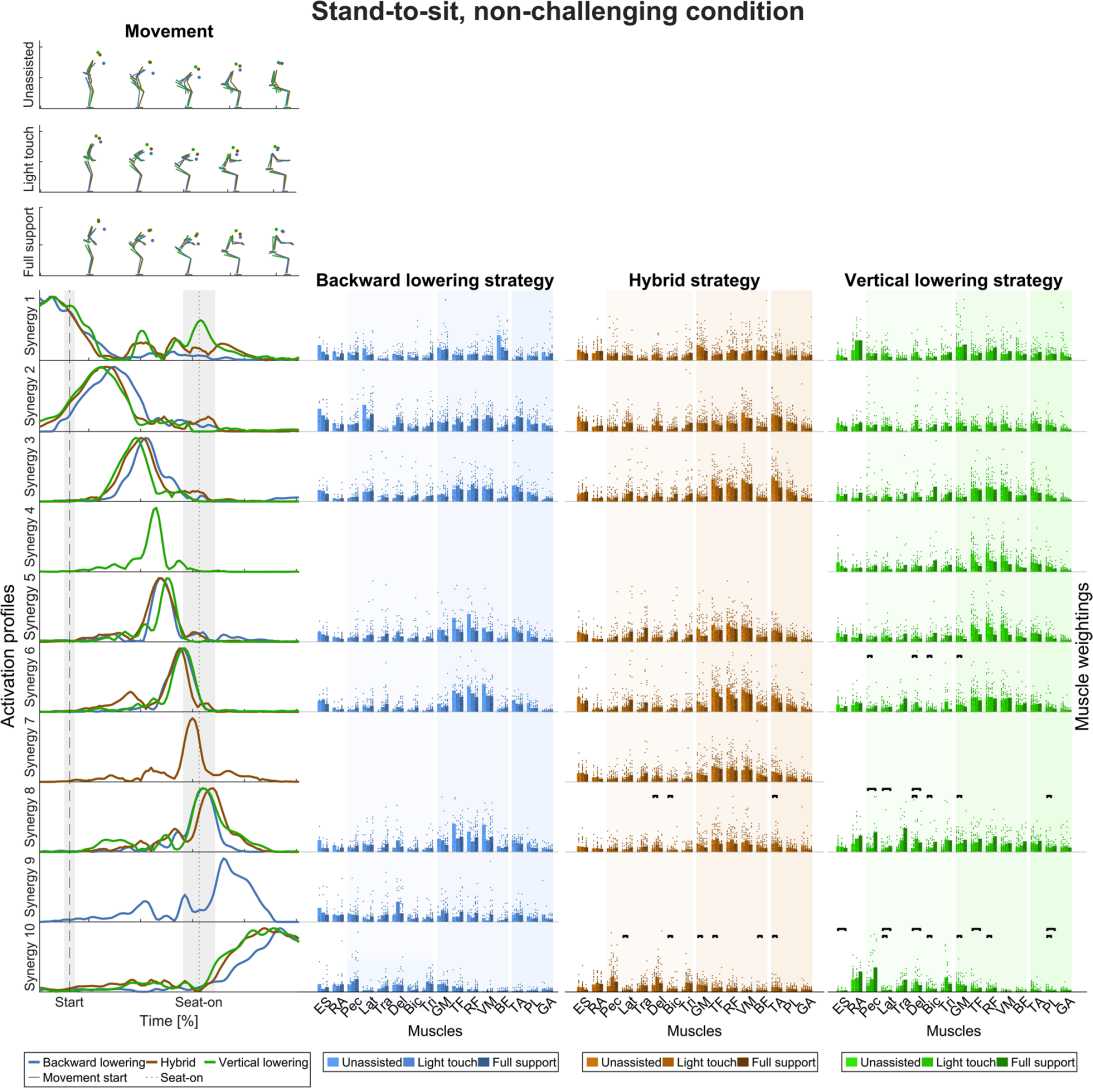
Temporal muscle synergies in the stand-to-sit movement and non-challenging condition. Top: Exemplary movements in the unassisted, light touch, and full support conditions (in rows) and movement strategies (color-coded). Bottom, left: Temporal muscle synergies sorted chronologically and color-coded according to movement strategy. The dashed line indicates movement start, i.e., the first identified CoM movement. The dotted line indicates seat-off. The timing of the three movement strategies was different, as indicated by the strategy-specific temporal synergies 4, 7, and 9. Bottom, right: Bar graphs show mean muscle weightings across the left and right side (except ES and RA), all trials within a strategy, and support condition, and single dots represent trial-specific weightings in two columns for the left and right limbs. The different color shades indicate the support conditions. The bars indicate statistically significant differences according to the LMM statistics (details in Additional file 2)

In the non-challenging task (Fig. 6), the activation profiles of the eight backward lowering, hybrid, and vertical lowering strategies were grouped into ten clusters. Most of the time, the timing across the three movement strategies did not differ. However, there was one distinct synergy for each of the movement strategies: the distinct backward lowering synergy is active after seat-on, the distinct hybrid synergy at seat-on, and the distinct vertical lowering synergy between movement start and seat-on.

**Fig. 7:**
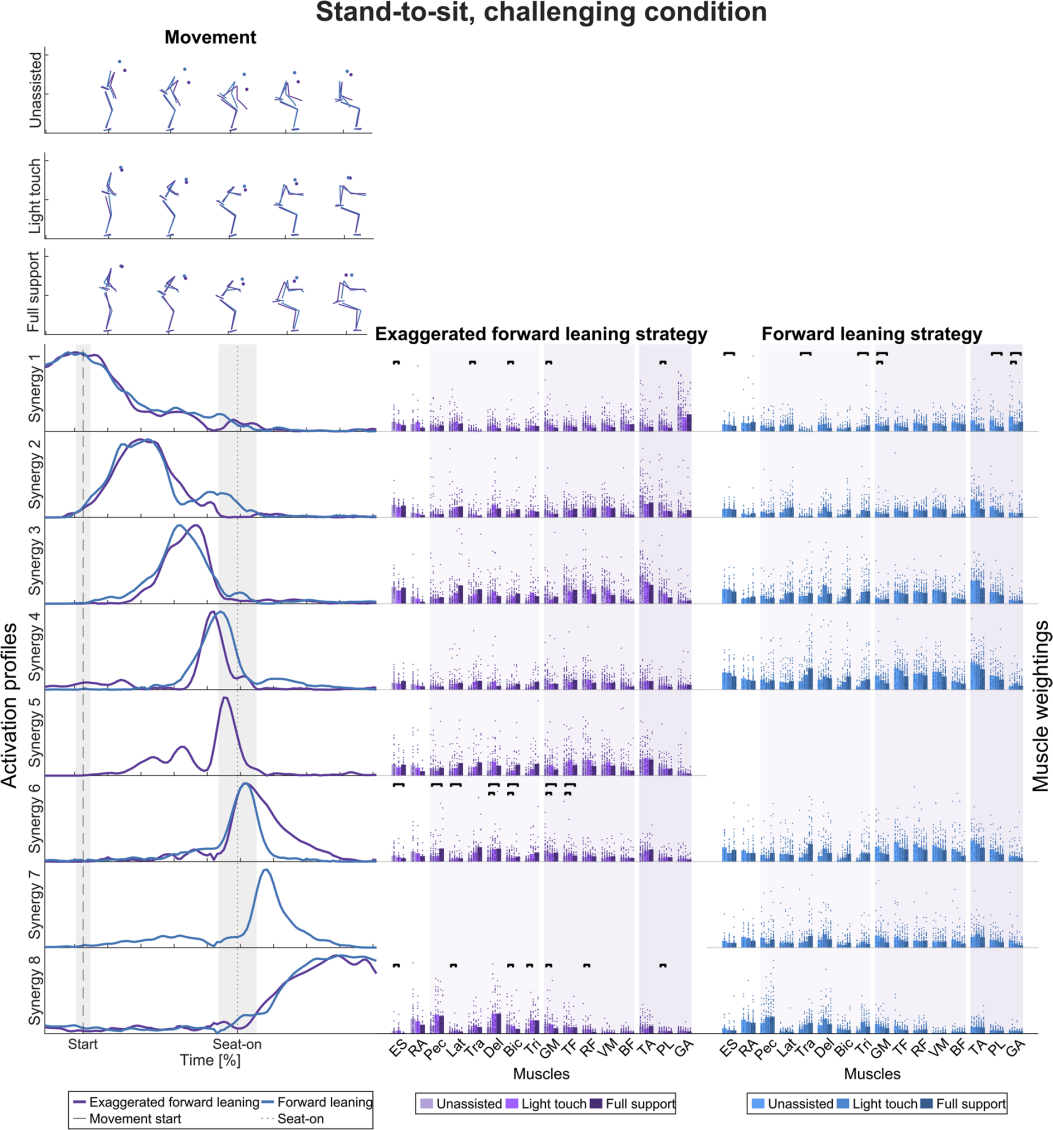
Temporal muscle synergies in the stand-to-sit movement and challenging condition. Top: Exemplary movements in the unassisted, light touch, and full support conditions (in rows) and movement strategies (color-coded). Bottom, left: Temporal muscle synergies sorted chronologically and color-coded according to the movement strategy. The dashed line indicates movement start, i.e., the first identified CoM movement. The dotted line indicates seat-off. The timing of the two movement strategies was different, as indicated by the strategy-specific temporal synergies 5 and 7. Bottom, right: Bar graphs show mean muscle weightings across the left and right side (except ES and RA), all trials within a strategy and support condition, and single dots represent trial-specific weightings in two columns for the left and right limbs. The different color shades indicate the support conditions. The bars indicate statistically significant differences according to the LMM statistics (details in Additional file 2)

In the challenging condition (Fig. 7), seven synergies of the exaggerated forward leaning and the forward leaning strategy were grouped into eight clusters. As in the non-challenging task, the timing did not differ between the two strategies most of the time. However, each strategy shows a distinct synergy. For the exaggerated forward leaning strategy, the synergy is active at seat-on, and for the forward leaning synergy after seat-on.

Similar to the sit-to-stand movements, the widths of the main synergy peaks (FWHM) were larger at movement start and end than in the middle, especially around seat-off (Table 3). Also, the finding that the timing of the temporal muscle synergies differs across the movement strategies is robust regarding the criterion for selecting the numbers of synergies (Supplementary Fig. 2, Additional file 1). Therefore, we accept our second hypothesis that muscle synergy activation differs across movement strategies for stand-to-sit movements.

### The degree of support influences muscle weightings

To test for differences in muscle weightings (right-hand columns of Figs. 4, 5, 6, and 7) across the support conditions (UA, LT, FS) with regard to hypothesis 3, that rollator support influences the weightings between upper body and lower limb muscles, LMMs were calculated. Detailed statistical results with p-values corrected for multiple testing are presented in Additional file 2.

#### Sit-to-stand movement strategies

In the non-challenging task, muscle weightings changed from the start of the movement onwards. The main change was an increased weighting of upper body muscles in the full support condition compared to unassisted across all movement strategies. Shortly before and at seat-off, muscle weightings showed a change with increased upper body and decreased lower limb muscle weightings in full support compared to the unassisted condition in the forward leaning and vertical rise strategy. After seat-off, minor changes were only observed in the vertical rise strategy.

More changes were observed in the challenging task than in the non-challenging task. At movement start, upper body muscles were more weighted in the full support condition than in the unassisted condition for all movement strategies. Shortly before and at seat-off, muscle weightings showed increased upper body and decreased lower limb muscle weightings in full support compared to the unassisted condition, as in the non-challenging task. After seat-off, we observed decreased weightings of the muscles acting around the ankle with light touch and full support, which is a specific finding of the challenging condition.

With regard to the sit-to-stand task, we accept the hypothesis that rollator support influences the weightings between upper body and lower limb muscles.

#### Stand-to-sit movement strategies

In the non-challenging task, no significant differences between the support conditions were observed for the backward lowering strategy. However, some changes were observed in the hybrid and vertical lowering strategy: at and after seat-on, the weightings of M. pectoralis major, latissimus dorsi, and arm muscles increased in the light touch and full support conditions.

In the challenging task, no significant changes in muscle weightings were observed in the forward leaning strategy after the movement began. However, muscle weightings changed at seat-on and movement end in the exaggerated forward leaning strategy. Comparable to the non-challenging task, the weightings of M. pectoralis major, latissimus dorsi, and arm muscles increased in the light touch and full support conditions. In addition, the weightings of M. gluteus medius and tensor fasciae latae decreased with support.

With regard to the sit-to-stand task, we accept the hypothesis that rollator support influences the weightings between upper body and lower limb muscles.

## Discussion

The study investigated the influence of rollator handle support on movement coordination in sit-to-stand and stand-to-sit movements while considering that people employ different movement strategies. Three support conditions were investigated: no assistance, light touch (haptic cue), and full support (supposed load reduction in the lower limbs). Furthermore, balance pads were placed underneath the young participants’ feet to make the movements challenging by increasing postural instability (non-challenging vs. challenging condition). We hypothesized that (1) temporal muscle synergies represent sit-to-stand and stand-to-sit EMG patterns with a different compactness than spatial muscle synergies. Furthermore, we hypothesized that (2) muscle synergy activation differs across movement strategies and that (3) rollator support influences the weightings between upper body and lower limb muscles.

### Temporal muscle synergies represent sit-to-stand and stand-to-sit EMG patterns more compactly than spatial muscle synergies

With six to ten temporal muscle synergies, low-dimensional representations of the sit-to-stand and stand-to-sit movement strategies with 30 EMGs were obtained with R^2^ values between 0.84 and 0.89. The number of synergies found was higher than in studies investigating sit-to-stand movements, in which three to four spatial muscle synergies explained 87–94% of the variance [51–56]. However, in these studies, no upper body muscle activity was measured, and no assistive device was used. In particular, previous studies also found that the number of synergies increased when participants used assistive devices, for example, Nordic walking sticks [59] or exoskeletons [60, 61] or while they were pushed up by the chair during sit-to-stand [58]. This supports the higher number of synergies found here compared to earlier sit-to-stand studies [51–56]. Also, spatial or spatiotemporal rather than temporal synergies were extracted in the referenced studies. Thus, comparisons regarding the reconstruction quality are limited. Nevertheless, the main functional groups, i.e., groups of coactivated muscles, identified with spatial synergies in the studies mentioned above largely align with the muscle weightings in our study.

We found that temporal muscle synergies provide a more compact representation than spatial synergies, which is supported by studies of upper-limb and postural tasks [43–45] and our finding that the change in slope (“knee”) in the R^2^ curve was more pronounced with temporal synergies was also found recently during postural tasks [44]. Spatial and temporal muscle synergies are two of several possible representations of modular control [42]. In general, the CNS might generate motor commands by a few sample-independent modules, which are thought to be shared across tasks and conditions [33] and are activated sample-dependently. The sample-independent part is thought to be stored in subcortical areas [42]. Consequently, with temporal synergies, it is assumed that activation profiles are stored and muscle weightings are sample-dependently composed [97–99]. The good reconstruction quality with temporal synergies hints that the CNS may store activation sequences for sit-to-stand and stand-to-sit movements, while it has to be acknowledged that the neural underpinning of muscle synergies is still debated [100, 101].

### The activation profiles of the temporal muscle synergies show similar shapes but differ in timing across movement strategies

Across all conditions, the activation profiles of the temporal muscle synergies mostly have a symmetric bell shape with a single point of maximum activation, similar to those found in the literature [46, 102]. The shapes are similar across the movement strategies, with a narrower width at seat-on and seat-off than at movement start and end. Furthermore, no support-specific synergies emerged with only high lower limb or upper-limb muscle weightings. However, the timing of the temporal muscle synergies differs across the movement strategies, which supports hypothesis 2. Similarly, Yang et al. [56] found that their momentum transfer and stabilization movement strategy only differed in the activation timing of the spatial muscle synergy, which was predominantly active around seat-off. This aligns with the activation times of the forward leaning, hybrid, and vertical rise strategies (synergies 2, 3, and 6).

Accordingly, the differences may stem from strategy-specific biomechanical requirements during this phase, e.g., in the forward leaning strategy to transfer the momentum generated by the upper body from the trunk to the thigh to stand up [103–105]. Also, comparing the exaggerated forward leaning to the forward leaning strategy in the sit-to-stand challenging movement, there are distinct activation profiles after seat-off while the body is erected, probably reflecting different biomechanical demands when participants keep their CoM over the BoS for longer in the exaggerated forward leaning strategy [25]. Hence, different movement strategies may emerge through different activation sequences, and rollator support does not provoke a different pattern of impulses.

### Temporal synergies reveal that tightness of control is increased in balance-critical phases

A recent study related temporal muscle synergies to the model of intermittent control of movement [44]. According to this model, the CNS sends pulsed commands that are transformed into muscle activation profiles [106]. The muscle activation profiles are adjusted in amplitude (here: sample-dependent muscle weightings) and finally result in motor output [107]. Sit-to-stand and stand-to-sit movements consist of multiple phases. Indeed, the literature has identified four to six phases, and even up to eleven [108–110], each of which has different requirements for motor control.

Numerous temporal synergies have been observed in this study, and their peaks are narrow, especially around the balance-critical seat-off and seat-on (see Table 3; [11, 13, 14]). Regarding the intermittent control model, these narrow, i.e., tight in time, peaks could reflect that each “control action” has a short duration. Accordingly, the CNS might tightly control the movements using multiple, successive pulsed commands to fulfill the demands of the phases’ different requirements. This tight CNS control is further supported by the increased number of temporal synergies in the challenging sit-to-stand condition, where the balance difficulty was increased. The exaggerated forward leaning strategy revealed three more synergies than the forward leaning strategy in the challenging sit-to-stand task. We previously aligned the former with the so-called “stabilization strategy," based on visual inspection of the stick figures, the hip and CoM-heel angles before seat-off, and the sagittal CoM trajectory [25]. We suggested it increases stability and safety when coping with the increased balance challenge due to the longer time the CoM resides inside the BoS [24, 25]. The higher number of temporal synergies found here may reflect this coping mechanism. However, the prolonged time keeping the CoM inside the BoS might increase the need for CNS control. In particular, Scarborough and colleagues propose that participants might need to sit back if adequate momentum could not be generated [111]. Therefore, the CNS probably increases the tightness of control to enable successful movements and prevent a sit-back. However, the high number of required narrow synergies, and thus a high demand for CNS control, may be critical for older people or people with disabilities. This aligns with Scarborough and colleagues’ argument that this strategy might not be the safest because both vertical and anterior momentum need to be generated adequately. Although this cannot be addressed with our healthy cohort, future studies may investigate whether people with reduced balance capacity in standing up and sitting down movements lack tight CNS control.

### Rollator support increases upper body and decreases lower limb muscle weightings

In support of hypothesis 3, we found that the weightings of upper body muscles increased with support across movement strategies in general, especially at seat-off and seat-on. Likewise, lower limb muscle weightings decreased with support. In particular, the weightings of muscles acting around the ankle decreased with support in the challenging sit-to-stand task.

Generally, these shifts find support in the literature. Suica et al. [112] found that walking with a rollator significantly reduced lower limb muscle activity in healthy subjects, and Ijmker et al.’s [113] EMG analysis with stroke survivors showed a drop in lower limb muscle activity when participants touched or held a handrail while walking on a treadmill. However, in these studies, participants walked rather than performing sit-to-stand or stand-to-sit movements, and only lower limb EMG was measured. Chihara and Seo [114] compared the activity of the anterior deltoid, triceps brachii, rectus femoris, and tibialis anterior in sit-to-stand movements with different handle heights positioned at either side of the participant and found that triceps activity increased while tibialis anterior activity decreased with higher handle height, while the anterior deltoid and rectus femoris were unaffected. This partly supports our findings, yet comparisons are limited as they did not compare muscle activity to unassisted standing up.

Interestingly, the lower limb muscles that often showed reduced weightings with increased support in our study, namely rectus femoris, gluteus medius, tibialis anterior, and peroneus longus, are highly relevant for both knee extension and balance [115–118], exemplifying the easing support of the rollator. These results could be very relevant to balance in older adults. Amiridis et al. [119] found that older adults rely more on hip muscles to maintain balance than younger adults in a number of static balance tasks, and a systematic review [120] found that hip abductor strength is critical for balance and to avoid falls. In particular, gluteus medius was among those with decreased activity with support in our study. Upper body muscle weightings, most often in the triceps, biceps brachii, and latissimus dorsi, increased with support across all movements. Their contributions likely enabled our participants to stand up and sit down with less lower limb muscle activity. The biceps flexes the elbow, and the latissimus dorsi adducts and medially rotates the humerus at the glenohumeral joints, bringing the body to the arms. The triceps is an elbow extensor [121], assisting in bringing the body upward against gravity and helping to balance the trunk during sit-to-stand [122]. Thus, the rollator-induced decrease in lower limb muscle weightings can be associated with a reduced leg strength required to fulfill both standing up and sitting down, following the roughly linear EMG/force relationship [123]. Therefore, if less force can or needs to be generated by the lower limb muscles, a rollator can help people with problems standing up. An in-depth analysis relating the changes in muscle weightings to the different movement strategies may further help to recommend specific movement strategies, e.g., strategies that most dramatically shift the muscle weightings from lower to the upper body.

### Limitations

Several potential limitations of this study need to be considered. First, our rollator-simulator device is heavier than a commercially available rollator and cannot dip or roll, which might influence how much people can lean and pull on the rollator handles. Secondly, we restricted the foot placement to be parallel and underneath the knees for standardization purposes, in line with other biomechanical studies [124]. While this is standard practice, it hindered the participants from pulling their feet backward to stand up, which is another everyday common movement strategy while standing up [22]. Thirdly, we included only young people. Even though we used balance pads to challenge them, the generalizability of the results to persons who are older or physically limited and dependent on a rollator may be limited. Nevertheless, Hanawa et al. [53] found that the synergies in standing up remain similar regardless of the participant’s age. Fourthly, despite explanation, familiarization trials, and careful observation, we cannot rule out the possibility that the participants applied more force to the handles in the light touch condition than is typical for studies with haptic cues [125]. Fifthly, the participants are low in number regarding using LMM statistics but at the highest level in the analysis; therefore, their number is not a major problem. Sixthly, even after careful consideration, we cannot exclude that other thresholds or matching procedures might have led to a different grouping of the temporal synergies’ activation profiles. Lastly, we observed symmetric activation of the muscles between the right and left limbs. Thus, future studies could focus on unilateral assessment of muscle activity and increase the resolution, e.g., by additionally measuring the hip adductors, M. gluteus maximus, all deltoid parts, and muscles acting on the wrist.

## Conclusion

This study investigated the rollator’s influence on the movement coordination for standing up and sitting down, while accounting for different movement strategies. Our temporal muscle synergy analysis found that the timing, but not the shape, of the temporal muscle synergies differs across the movement strategies. Further, we found that the CNS tightly controls standing up and sitting down movements, especially during the balance-critical phases around seat-off and seat-on. Additionally, no support-specific synergies were found, suggesting that the CNS does not need to alter the control if a rollator is used. However, muscle weightings shifted from the lower limbs to the upper body with increased support. In sum, we suggest that people struggling with rollator use practice movement strategies requiring less tight CNS control, like the forward leaning instead of the exaggerated forward leaning strategy to stand up. Further studies should investigate if these findings hold for older and fall-prone individuals, to enable safe and efficient recommendations on rollator usage.

## Supplementary Information

Additional file 1.

Additional file 2.

## Data Availability

All data generated or analyzed during this study are included in this published article and its supplementary information files.

## Abbreviations

BF: Musculus biceps femoris
Bic: Musculus biceps brachii
BoS: Base of support
CNS: Central nervous system
CoM: Center of mass
Del: Musculus deltoideus
EMG: Electromyography
ES: Musculus erector spinae
FS: Full support
FWHM: Full-width at half-maximum
GA: Musculus gastrocnemius
GM: Musculus gluteus medius
GRF: Ground reaction force
LMM: Linear mixed model
LT: Light touch
Lat: Musculus latissimus dorsi
MSE: Mean squared residual error
NMF: Non-negative matrix factorization
PL: Musculus peroneus longus
Pec: Musculus pectoralis major
RA: Musculus rectus abdominis
RF: Musculus rectus femoris
TA: Musculus tibialis anterior
TFL: Musculus tensor fasciae latae
Tra: Musculus trapezius
Tri: Musculus triceps brachii
UA: Unassisted
VM: Musculus vastus medialis
