# Supplementary material for: The central nervous system adjusts muscle synergy structure and tightly controls rollator-supported transitions between sitting and standing
Source: J Neuroeng Rehabil. 2025 Apr 25;22:96. doi: 10.1186/s12984-025-01622-y (PMC12032710; doi:10.1186/s12984-025-01622-y)
Supplement: Supplementary file 1 — Additional file 1. [file 12984_2025_1622_MOESM1_ESM.docx]

The central nervous system adjusts muscle synergy structure and tightly controls rollator-supported transitions between sitting and standing

**- Supplementary Material –**

Michael Herzog^1,2*^, Frieder C. Krafft^2,3,4^, Janis Fiedler^5^, Denise J. Berger^6,7^, Lizeth H. Sloot^2,3,8^, Andrea d’Avella^6,9†^, Thorsten Stein^1,2†^

^1^ BioMotion Center, Institute of Sports and Sports Science, Karlsruhe Institute of Technology (KIT), Karlsruhe, Germany

^2^ HEiKA – Heidelberg Karlsruhe Strategic Partnership, Heidelberg University, Karlsruhe Institute of Technology (KIT), Heidelberg & Karlsruhe, Germany

^3^ Institute of Computer Engineering, Heidelberg University, Heidelberg, Germany

^4^ Center of Prevention, Diagnostic and Performance, Center of Orthopaedics Hohenlohe, Künzelsau, Germany

^5^ Institute of Sports and Sports Science, Karlsruhe Institute of Technology, Karlsruhe, Germany

^6^ Laboratory of Neuromotor Physiology, IRCCS Fondazione Santa Lucia, Rome, Italy

^7^ Department of Systems Medicine and Centre of Space Bio-medicine, University of Rome Tor Vergata, Rome, Italy

^8^ Translational and Clinical Research Institute, Newcastle University, Newcastle upon Tyne, UK

^9^ Department of Biology, University of Rome Tor Vergata, Rome, Italy

* Correspondence:
Michael Herzog

Karlsruhe Institute of Technology

BioMotion Center

Engler-Bunte Ring 15

76131 Karlsruhe, Germany

E-Mail: Michael.Herzog@kit.edu

^†^ These authors have contributed equally to this work.

# Trials with corrupt EMG recordings

Table 1: Trials with corrupt EMG signals. Except for these trials, all EMG recordings were valid. l: left side, r: right side.

| **Participant** | **Movement and conditions** | **Trial** | **Strategy** | **Bic** | **Lat** | **Tra** | **GM** | **TF** | **RF** | **VM** | **BF** | **TA** | **PL** | **GA** |
| --- | --- | --- | --- | --- | --- | --- | --- | --- | --- | --- | --- | --- | --- | --- |
| 2 | Stand-to-sit,  non-challenging, unassisted | 1 | Vertical lowering |  |  |  | l |  |  |  |  |  |  |  |
| 2 | Stand-to-sit,  non-challenging, unassisted | 2 | Vertical lowering |  |  |  | l |  |  |  |  |  |  |  |
| 2 | Stand-to-sit,  non-challenging, unassisted | 3 | Vertical lowering |  |  |  | l |  |  |  |  |  |  |  |
| 2 | Sit-to-stand,  non-challenging, light touch | 1 | Hybrid |  |  |  |  | l, r | l, r | l, r | l, r | l, r | l, r | l, r |
| 2 | Sit-to-stand,  non-challenging, light touch | 2 | Hybrid |  |  |  |  | l, r | l, r | l, r | l, r | l, r | l, r | l, r |
| 2 | Sit-to-stand,  non-challenging, light touch | 3 | Hybrid |  |  |  |  | l, r | l, r | l, r | l, r | l, r | l, r | l, r |
| 2 | Stand-to-sit,  non-challenging, light touch | 1 | Vertical lowering |  |  |  |  | l, r | l, r | l, r | l, r | l, r | l, r | l, r |
| 2 | Stand-to-sit,  non-challenging, light touch | 2 | Vertical lowering |  |  |  |  | l, r | l, r | l, r | l, r | l, r | l, r | l, r |
| 2 | Stand-to-sit,  non-challenging, light touch | 3 | Vertical lowering |  |  |  |  | l, r | l, r | l, r | l, r | l, r | l, r | l, r |
| 2 | Sit-to-stand,  non-challenging, full support | 1 | Vertical rise |  |  |  |  | l, r | l, r | l, r | l, r | l, r | l, r | l, r |
| 2 | Sit-to-stand,  non-challenging, full support | 2 | Hybrid |  |  |  |  | l, r | l, r | l, r | l, r | l, r | l, r | l, r |
| 2 | Sit-to-stand, non-challenging,  full support | 2 | Exaggerated forward leaning |  |  |  |  | l, r | l, r | l, r | l, r | l, r | l, r | l, r |
| 2 | Stand-to-sit, non-challenging,  full support | 1 | Vertical lowering |  |  |  |  |  | l | l |  | l, r | l, r | l, r |
| 2 | Stand-to-sit, challenging,  light touch | 2 | Backward lowering |  |  |  |  |  |  |  |  | l, r | l, r | l, r |
| 4 | Sit-to-stand, challenging,  light touch | 2 | Forward leaning | l, r |  |  |  | r | l | l, r | l, r | l, r | l, r | r |
| 4 | Stand-to-sit, challenging,  light touch | 2 | Exaggerated forward leaning | l, r |  |  |  | r | l | l, r | l, r | l, r | l, r | r |
| 5 | Sit-to-stand,  non-challenging, light touch | 1 | Hybrid |  | l |  |  | l | l |  |  | l, r | l, r | l, r |
| 5 | Stand-to-sit,  non-challenging, light touch | 1 | Backward lowering |  | l |  |  | l | l |  |  | l, r | l, r | l, r |
| 9 | Sit-to-stand,  non-challenging, unassisted | 3 | Hybrid |  |  | r |  | l | l |  |  | l, r | r | l, r |
| 9 | Stand-to-sit,  non-challenging, unassisted | 3 | Vertical lowering |  |  | r |  | l | l |  |  | l, r | r | l, r |
| 9 | Sit-to-stand, challenging, unassisted | 3 | Exaggerated forward leaning |  |  |  |  | l | l |  |  | l, r | r | l |
| 9 | Stand-to-sit, challenging, unassisted | 3 | Exaggerated forward leaning |  |  |  |  | l | l |  |  | l, r | r | l |
| 12 | Sit-to-stand,  non-challenging, light touch | 2 | Forward leaning |  | l |  |  |  | l, r | l, r | l | l, r | r | l, r |
| 12 | Stand-to-sit,  non-challenging, light touch | 2 | Vertical lowering |  | l |  |  |  | l, r | l, r | l | l, r | r | l, r |

# Distribution of trials among strategies


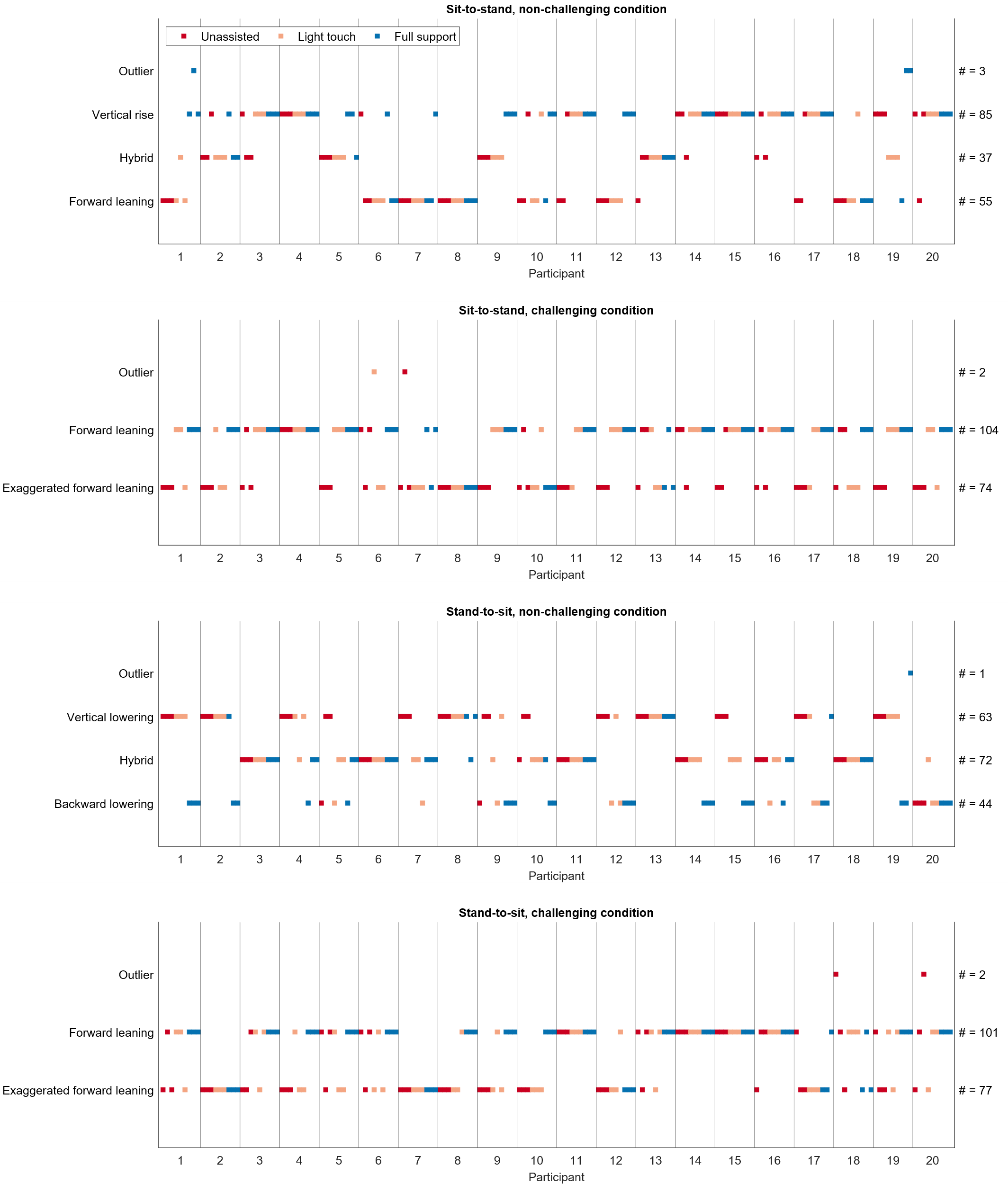


Figure 1: Distribution of trials among strategies. One dot represents one trial. The row indicates the strategy to which it belongs. The column shows to which participant it belongs. The support conditions are color-coded as indicated by the legend. The labels on the right y-axis show how many trials were associated with the strategy written on the left y-axis. This figure is taken from [1].

# Differences in the temporal composition remain with the R² > 0.9 (N_strat_^*^) criterion


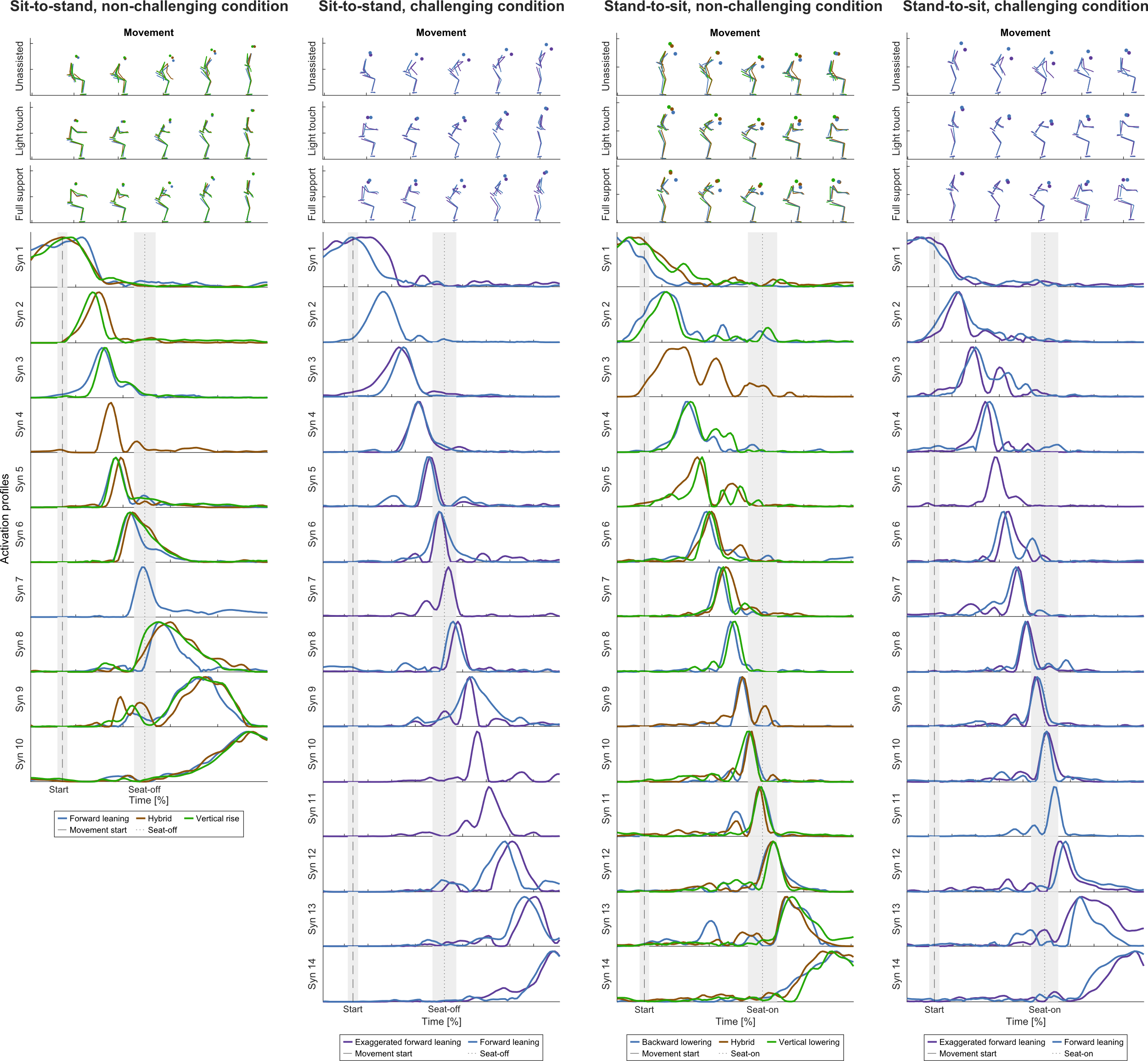


Figure 2: Temporal synergies for every movement strategy with the R² > 0.9 (N_strat_^*^) criterion. Clustered and ordered chronologically.

# References

1. Herzog M, Krafft FC, Stetter BJ, d’Avella A, Sloot LH, Stein T. Rollator usage lets young individuals switch movement strategies in sit-to-stand and stand-to-sit tasks. Sci Rep. 2023;13:16901.
