# Supplementary material for: The central nervous system adjusts muscle synergy structure and tightly controls rollator-supported transitions between sitting and standing
Source: J Neuroeng Rehabil. 2025 Apr 25;22:96. doi: 10.1186/s12984-025-01622-y (PMC12032710; doi:10.1186/s12984-025-01622-y)
Supplement: Supplementary file 2 — Additional file 2. [file 12984_2025_1622_MOESM2_ESM.docx]

The central nervous system adjusts muscle synergy structure and tightly controls rollator-supported transitions between sitting and standing

**- Supplementary Material –**

Michael Herzog^1,2*^, Frieder C. Krafft^2,3,4^, Janis Fiedler^5^, Denise J. Berger^6,7^, Lizeth H. Sloot^2,3,8^, Andrea d’Avella^6,9†^, Thorsten Stein^1,2†^

^1^ BioMotion Center, Institute of Sports and Sports Science, Karlsruhe Institute of Technology (KIT), Karlsruhe, Germany

^2^ HEiKA – Heidelberg Karlsruhe Strategic Partnership, Heidelberg University, Karlsruhe Institute of Technology (KIT), Heidelberg & Karlsruhe, Germany

^3^ Institute of Computer Engineering, Heidelberg University, Heidelberg, Germany

^4^ Center of Prevention, Diagnostic and Performance, Center of Orthopaedics Hohenlohe, Künzelsau, Germany

^5^ Institute of Sports and Sports Science, Karlsruhe Institute of Technology, Karlsruhe, Germany

^6^ Laboratory of Neuromotor Physiology, IRCCS Fondazione Santa Lucia, Rome, Italy

^7^ Department of Systems Medicine and Centre of Space Bio-medicine, University of Rome Tor Vergata, Rome, Italy

^8^ Translational and Clinical Research Institute, Newcastle University, Newcastle upon Tyne, UK

^9^ Department of Biology, University of Rome Tor Vergata, Rome, Italy

* Correspondence:
Michael Herzog

Karlsruhe Institute of Technology

BioMotion Center

Engler-Bunte Ring 15

76131 Karlsruhe, Germany

E-Mail: Michael.Herzog@kit.edu

^†^ These authors have contributed equally to this work.

# Detailed linear mixed model statistics

## Sit-to-stand, non-challenging condition

**Detailed statistics: sit-to-stand, non-challenging condition, forward leaning strategy, synergy 1**

Reference group: UA

| Model information | Values |
| --- | --- |
| Number of observations | 864 |
| Number of participants | 12 |
| Intraclass correlation coefficient (ICC) | 0.502 |

| Fixed effects | Estimate | Lower 95% CI | Upper 95% CI | t | p |
| --- | --- | --- | --- | --- | --- |
| (Intercept) | 0.023 | 0.017 | 0.029 | 7.614 | <.001 |
| LT | 0.008 | 0.003 | 0.014 | 2.941 | 0.047 |
| FS | 0.010 | 0.004 | 0.016 | 3.059 | 0.032 |

| Random effects | Estimate | Lower 95% CI | Upper 95% CI |
| --- | --- | --- | --- |
| (Intercept) | 0.008 | 0.005 | 0.015 |
| Error | 0.034 | 0.032 | 0.035 |

Reference group: LT

| Fixed effects | Estimate | Lower 95% CI | Upper 95% CI | t | p |
| --- | --- | --- | --- | --- | --- |
| (Intercept) | 0.031 | 0.025 | 0.038 | 9.167 | <.001 |
| UA | -0.008 | -0.014 | -0.003 | -2.941 | 0.047 |
| FS | 0.002 | -0.005 | 0.008 | 0.513 | 1.000 |

| Random effects | Estimate | Lower 95% CI | Upper 95% CI |
| --- | --- | --- | --- |
| (Intercept) | 0.008 | 0.005 | 0.015 |
| Error | 0.034 | 0.032 | 0.035 |

Post-hoc UA vs. LT

| Fixed effects | Estimate | Lower 95% CI | Upper 95% CI | t | p |
| --- | --- | --- | --- | --- | --- |
| RA:LT | -0.007 | -0.023 | 0.008 | -0.899 | 0.738 |
| ES:LT | 0.002 | -0.014 | 0.017 | 0.195 | 1.000 |
| Pec:LT | -0.003 | -0.018 | 0.013 | -0.339 | 1.000 |
| Lat:LT | 0.008 | -0.007 | 0.024 | 1.055 | 0.584 |
| Tra:LT | 0.052 | 0.036 | 0.067 | 6.553 | <.001 |
| Del:LT | 0.005 | -0.010 | 0.021 | 0.661 | 1.000 |
| Bic:LT | 0.001 | -0.015 | 0.016 | 0.115 | 1.000 |
| Tri:LT | 0.002 | -0.014 | 0.017 | 0.251 | 1.000 |
| GM:LT | -0.009 | -0.025 | 0.006 | -1.160 | 0.493 |
| TF:LT | -0.015 | -0.030 | 0.001 | -1.886 | 0.119 |
| RF:LT | -0.009 | -0.024 | 0.007 | -1.117 | 0.528 |
| VM:LT | -0.007 | -0.022 | 0.009 | -0.869 | 0.770 |
| BF:LT | -0.006 | -0.022 | 0.009 | -0.819 | 0.826 |
| TA:LT | -0.006 | -0.022 | 0.009 | -0.805 | 0.843 |
| PL:LT | -0.005 | -0.020 | 0.011 | -0.582 | 1.000 |

| Random effects | Estimate | Lower 95% CI | Upper 95% CI |
| --- | --- | --- | --- |
| (Intercept) | 0.009 | 0.006 | 0.015 |
| Error | 0.026 | 0.025 | 0.027 |

Post-hoc UA vs. FS

| Fixed effects | Estimate | Lower 95% CI | Upper 95% CI | t | p |
| --- | --- | --- | --- | --- | --- |
| RA:FS | -0.024 | -0.041 | -0.007 | -2.725 | 0.013 |
| ES:FS | -0.008 | -0.025 | 0.009 | -0.901 | 0.735 |
| Pec:FS | 0.007 | -0.010 | 0.024 | 0.795 | 0.854 |
| Lat:FS | 0.009 | -0.008 | 0.027 | 1.069 | 0.571 |
| Tra:FS | 0.058 | 0.040 | 0.075 | 6.547 | <.001 |
| Del:FS | -0.020 | -0.038 | -0.003 | -2.323 | 0.041 |
| Bic:FS | 0.048 | 0.031 | 0.065 | 5.454 | <.001 |
| Tri:FS | 0.020 | 0.002 | 0.037 | 2.241 | 0.051 |
| GM:FS | -0.025 | -0.042 | -0.008 | -2.853 | 0.009 |
| TF:FS | -0.018 | -0.035 | -0.001 | -2.022 | 0.087 |
| RF:FS | -0.002 | -0.019 | 0.016 | -0.178 | 1.000 |
| VM:FS | -0.003 | -0.020 | 0.015 | -0.306 | 1.000 |
| BF:FS | -0.008 | -0.025 | 0.009 | -0.924 | 0.711 |
| TA:FS | -0.015 | -0.032 | 0.003 | -1.646 | 0.200 |
| PL:FS | -0.011 | -0.028 | 0.006 | -1.263 | 0.414 |

| Random effects | Estimate | Lower 95% CI | Upper 95% CI |
| --- | --- | --- | --- |
| (Intercept) | 0.009 | 0.006 | 0.015 |
| Error | 0.026 | 0.025 | 0.027 |

**Detailed statistics: sit-to-stand, non-challenging condition, forward leaning strategy, synergy 3**

Reference group: UA

| Model information | Values |
| --- | --- |
| Number of observations | 864 |
| Number of participants | 12 |
| ICC | 0.566 |

| Fixed effects | Estimate | Lower 95% CI | Upper 95% CI | t | p |
| --- | --- | --- | --- | --- | --- |
| (Intercept) | 0.020 | 0.014 | 0.027 | 6.490 | <.001 |
| LT | 0.016 | 0.011 | 0.021 | 5.924 | <.001 |
| FS | 0.011 | 0.005 | 0.017 | 3.448 | 0.008 |

| Random effects | Estimate | Lower 95% CI | Upper 95% CI |
| --- | --- | --- | --- |
| (Intercept) | 0.009 | 0.005 | 0.015 |
| Error | 0.032 | 0.031 | 0.034 |

Reference group: LT

| Fixed effects | Estimate | Lower 95% CI | Upper 95% CI | t | p |
| --- | --- | --- | --- | --- | --- |
| (Intercept) | 0.036 | 0.029 | 0.043 | 10.350 | <.001 |
| UA | -0.016 | -0.021 | -0.011 | -5.924 | <.001 |
| FS | -0.005 | -0.011 | 0.001 | -1.601 | 1.000 |

| Random effects | Estimate | Lower 95% CI | Upper 95% CI |
| --- | --- | --- | --- |
| (Intercept) | 0.009 | 0.005 | 0.015 |
| Error | 0.032 | 0.031 | 0.034 |

Post-hoc UA vs. LT

| Fixed effects | Estimate | Lower 95% CI | Upper 95% CI | t | p |
| --- | --- | --- | --- | --- | --- |
| RA:LT | -0.014 | -0.032 | 0.003 | -1.660 | 0.195 |
| ES:LT | 0.015 | -0.002 | 0.032 | 1.744 | 0.163 |
| Pec:LT | -0.014 | -0.031 | 0.004 | -1.555 | 0.241 |
| Lat:LT | 0.009 | -0.009 | 0.026 | 0.980 | 0.655 |
| Tra:LT | -0.008 | -0.025 | 0.009 | -0.937 | 0.698 |
| Del:LT | 0.017 | -0.000 | 0.034 | 1.937 | 0.106 |
| Bic:LT | 0.002 | -0.015 | 0.019 | 0.191 | 1.000 |
| Tri:LT | -0.002 | -0.019 | 0.015 | -0.213 | 1.000 |
| GM:LT | -0.006 | -0.023 | 0.011 | -0.698 | 0.971 |
| TF:LT | -0.001 | -0.018 | 0.016 | -0.118 | 1.000 |
| RF:LT | 0.009 | -0.009 | 0.026 | 0.976 | 0.658 |
| VM:LT | 0.023 | 0.006 | 0.040 | 2.622 | 0.018 |
| BF:LT | -0.008 | -0.025 | 0.009 | -0.919 | 0.717 |
| TA:LT | -0.004 | -0.021 | 0.014 | -0.412 | 1.000 |
| PL:LT | -0.006 | -0.023 | 0.011 | -0.664 | 1.000 |

| Random effects | Estimate | Lower 95% CI | Upper 95% CI |
| --- | --- | --- | --- |
| (Intercept) | 0.009 | 0.006 | 0.016 |
| Error | 0.029 | 0.027 | 0.030 |

Post-hoc UA vs. FS

| Fixed effects | Estimate | Lower 95% CI | Upper 95% CI | t | p |
| --- | --- | --- | --- | --- | --- |
| RA:FS | -0.007 | -0.026 | 0.012 | -0.720 | 0.944 |
| ES:FS | -0.009 | -0.028 | 0.010 | -0.929 | 0.706 |
| Pec:FS | -0.004 | -0.023 | 0.015 | -0.438 | 1.000 |
| Lat:FS | 0.017 | -0.002 | 0.036 | 1.756 | 0.159 |
| Tra:FS | -0.013 | -0.032 | 0.006 | -1.312 | 0.380 |
| Del:FS | -0.001 | -0.020 | 0.018 | -0.119 | 1.000 |
| Bic:FS | 0.030 | 0.010 | 0.049 | 3.036 | 0.005 |
| Tri:FS | 0.006 | -0.013 | 0.025 | 0.611 | 1.000 |
| GM:FS | -0.013 | -0.032 | 0.006 | -1.343 | 0.359 |
| TF:FS | 0.004 | -0.015 | 0.023 | 0.383 | 1.000 |
| RF:FS | 0.003 | -0.016 | 0.023 | 0.359 | 1.000 |
| VM:FS | 0.019 | 0.000 | 0.038 | 1.988 | 0.094 |
| BF:FS | -0.007 | -0.026 | 0.013 | -0.672 | 1.000 |
| TA:FS | -0.007 | -0.027 | 0.012 | -0.764 | 0.890 |
| PL:FS | -0.008 | -0.027 | 0.011 | -0.834 | 0.809 |

| Random effects | Estimate | Lower 95% CI | Upper 95% CI |
| --- | --- | --- | --- |
| (Intercept) | 0.009 | 0.006 | 0.016 |
| Error | 0.029 | 0.027 | 0.030 |

**Detailed statistics: sit-to-stand, non-challenging condition, forward leaning strategy, synergy 4**

Reference group: UA

| Model information | Values |
| --- | --- |
| Number of observations | 864 |
| Number of participants | 12 |
| ICC | 0.434 |

| Fixed effects | Estimate | Lower 95% CI | Upper 95% CI | t | p |
| --- | --- | --- | --- | --- | --- |
| (Intercept) | 0.030 | 0.024 | 0.036 | 10.441 | <.001 |
| LT | 0.009 | 0.003 | 0.015 | 3.133 | 0.025 |
| FS | 0.005 | -0.001 | 0.012 | 1.589 | 1.000 |

| Random effects | Estimate | Lower 95% CI | Upper 95% CI |
| --- | --- | --- | --- |
| (Intercept) | 0.008 | 0.004 | 0.013 |
| Error | 0.035 | 0.033 | 0.037 |

Reference group: LT

| Fixed effects | Estimate | Lower 95% CI | Upper 95% CI | t | p |
| --- | --- | --- | --- | --- | --- |
| (Intercept) | 0.039 | 0.033 | 0.046 | 11.893 | <.001 |
| UA | -0.009 | -0.015 | -0.003 | -3.133 | 0.025 |
| FS | -0.004 | -0.010 | 0.003 | -1.083 | 1.000 |

| Random effects | Estimate | Lower 95% CI | Upper 95% CI |
| --- | --- | --- | --- |
| (Intercept) | 0.008 | 0.004 | 0.013 |
| Error | 0.035 | 0.033 | 0.037 |

Post-hoc UA vs. LT

| Fixed effects | Estimate | Lower 95% CI | Upper 95% CI | t | p |
| --- | --- | --- | --- | --- | --- |
| RA:LT | -0.011 | -0.028 | 0.006 | -1.281 | 0.201 |
| ES:LT | -0.019 | -0.036 | -0.002 | -2.187 | 0.029 |
| Pec:LT | -0.008 | -0.025 | 0.009 | -0.907 | 0.365 |
| Lat:LT | 0.013 | -0.004 | 0.029 | 1.452 | 0.147 |
| Tra:LT | -0.003 | -0.020 | 0.014 | -0.375 | 0.708 |
| Del:LT | 0.032 | 0.015 | 0.049 | 3.674 | <.001 |
| Bic:LT | 0.010 | -0.007 | 0.027 | 1.123 | 0.262 |
| Tri:LT | -0.008 | -0.025 | 0.009 | -0.948 | 0.343 |
| GM:LT | -0.011 | -0.028 | 0.006 | -1.295 | 0.196 |
| TF:LT | 0.008 | -0.009 | 0.025 | 0.955 | 0.340 |
| RF:LT | 0.002 | -0.014 | 0.019 | 0.289 | 0.772 |
| VM:LT | 0.005 | -0.012 | 0.022 | 0.553 | 0.580 |
| BF:LT | 0.007 | -0.010 | 0.024 | 0.815 | 0.415 |
| TA:LT | -0.003 | -0.020 | 0.014 | -0.374 | 0.709 |
| PL:LT | -0.007 | -0.024 | 0.010 | -0.796 | 0.426 |

| Random effects | Estimate | Lower 95% CI | Upper 95% CI |
| --- | --- | --- | --- |
| (Intercept) | 0.008 | 0.005 | 0.013 |
| Error | 0.028 | 0.027 | 0.030 |

**Detailed statistics: sit-to-stand, non-challenging condition, forward leaning strategy, synergy 5**

Reference group: UA

| Model information | Values |
| --- | --- |
| Number of observations | 864 |
| Number of participants | 12 |
| ICC | 0.598 |

| Fixed effects | Estimate | Lower 95% CI | Upper 95% CI | t | p |
| --- | --- | --- | --- | --- | --- |
| (Intercept) | 0.039 | 0.032 | 0.047 | 10.559 | <0.001 |
| LT | -0.008 | -0.014 | -0.002 | -2.621 | 0.125 |
| FS | -0.012 | -0.018 | -0.005 | -3.290 | 0.015 |

| Random effects | Estimate | Lower 95% CI | Upper 95% CI |
| --- | --- | --- | --- |
| (Intercept) | 0.011 | 0.007 | 0.017 |
| Error | 0.036 | 0.035 | 0.038 |

Reference group: LT

| Fixed effects | Estimate | Lower 95% CI | Upper 95% CI | t | p |
| --- | --- | --- | --- | --- | --- |
| (Intercept) | 0.031 | 0.023 | 0.039 | 7.661 | <0.001 |
| UA | 0.008 | 0.002 | 0.014 | 2.621 | 0.125 |
| FS | -0.004 | -0.011 | 0.003 | -1.019 | 1.000 |

| Random effects | Estimate | Lower 95% CI | Upper 95% CI |
| --- | --- | --- | --- |
| (Intercept) | 0.011 | 0.007 | 0.017 |
| Error | 0.036 | 0.035 | 0.038 |

Post-hoc UA vs. FS

| Fixed effects | Estimate | Lower 95% CI | Upper 95% CI | t | p |
| --- | --- | --- | --- | --- | --- |
| RA:FS | 0.004 | -0.013 | 0.022 | 0.509 | 0.611 |
| ES:FS | -0.035 | -0.052 | -0.018 | -3.977 | <0.001 |
| Pec:FS | 0.026 | 0.009 | 0.043 | 2.959 | 0.003 |
| Lat:FS | 0.019 | 0.002 | 0.036 | 2.144 | 0.032 |
| Tra:FS | 0.015 | -0.002 | 0.032 | 1.739 | 0.082 |
| Del:FS | 0.040 | 0.023 | 0.057 | 4.545 | <0.001 |
| Bic:FS | 0.024 | 0.007 | 0.041 | 2.752 | 0.006 |
| Tri:FS | 0.020 | 0.003 | 0.037 | 2.318 | 0.021 |
| GM:FS | 0.004 | -0.013 | 0.021 | 0.440 | 0.660 |
| TF:FS | -0.031 | -0.048 | -0.013 | -3.504 | <0.001 |
| RF:FS | -0.043 | -0.060 | -0.026 | -4.965 | <0.001 |
| VM:FS | -0.025 | -0.042 | -0.008 | -2.834 | 0.005 |
| BF:FS | -0.009 | -0.026 | 0.008 | -1.030 | 0.303 |
| TA:FS | -0.013 | -0.030 | 0.004 | -1.490 | 0.137 |
| PL:FS | -0.002 | -0.019 | 0.015 | -0.212 | 0.833 |

| Random effects | Estimate | Lower 95% CI | Upper 95% CI |
| --- | --- | --- | --- |
| (Intercept) | 0.011 | 0.007 | 0.017 |
| Error | 0.026 | 0.024 | 0.027 |

**Detailed statistics: sit-to-stand, non-challenging condition, forward leaning strategy, synergy 6**

Reference group: UA

| Model information | Values |
| --- | --- |
| Number of observations | 864 |
| Number of participants | 12 |
| ICC | 0.576 |

| Fixed effects | Estimate | Lower 95% CI | Upper 95% CI | t | p |
| --- | --- | --- | --- | --- | --- |
| (Intercept) | 0.031 | 0.024 | 0.037 | 9.299 | <0.001 |
| LT | 0.001 | -0.005 | 0.006 | 0.202 | 1.000 |
| FS | 0.000 | -0.006 | 0.006 | 0.011 | 1.000 |

| Random effects | Estimate | Lower 95% CI | Upper 95% CI |
| --- | --- | --- | --- |
| (Intercept) | 0.010 | 0.006 | 0.017 |
| Error | 0.033 | 0.032 | 0.035 |

Reference group: LT

| Fixed effects | Estimate | Lower 95% CI | Upper 95% CI | t | p |
| --- | --- | --- | --- | --- | --- |
| (Intercept) | 0.031 | 0.024 | 0.039 | 8.560 | <0.001 |
| UA | -0.001 | -0.006 | 0.005 | -0.202 | 1.000 |
| FS | -0.001 | -0.007 | 0.006 | -0.159 | 1.000 |

| Random effects | Estimate | Lower 95% CI | Upper 95% CI |
| --- | --- | --- | --- |
| (Intercept) | 0.010 | 0.006 | 0.017 |
| Error | 0.033 | 0.032 | 0.035 |

**Detailed statistics: sit-to-stand, non-challenging condition, forward leaning strategy, synergy 7**

Reference group: UA

| Model information | Values |
| --- | --- |
| Number of observations | 864 |
| Number of participants | 12 |
| ICC | 0.339 |

| Fixed effects | Estimate | Lower 95% CI | Upper 95% CI | t | p |
| --- | --- | --- | --- | --- | --- |
| (Intercept) | 0.029 | 0.024 | 0.034 | 12.360 | <0.001 |
| LT | 0.003 | -0.002 | 0.008 | 1.137 | 1.000 |
| FS | 0.007 | 0.001 | 0.013 | 2.137 | 0.460 |

| Random effects | Estimate | Lower 95% CI | Upper 95% CI |
| --- | --- | --- | --- |
| (Intercept) | 0.006 | 0.003 | 0.010 |
| Error | 0.033 | 0.031 | 0.034 |

Reference group: LT

| Fixed effects | Estimate | Lower 95% CI | Upper 95% CI | t | p |
| --- | --- | --- | --- | --- | --- |
| (Intercept) | 0.032 | 0.027 | 0.038 | 11.705 | <0.001 |
| UA | -0.003 | -0.008 | 0.002 | -1.137 | 1.000 |
| FS | 0.004 | -0.003 | 0.010 | 1.106 | 1.000 |

| Random effects | Estimate | Lower 95% CI | Upper 95% CI |
| --- | --- | --- | --- |
| (Intercept) | 0.006 | 0.003 | 0.010 |
| Error | 0.033 | 0.031 | 0.034 |

**Detailed statistics: sit-to-stand, non-challenging condition, forward leaning strategy, synergy 8**

Reference group: UA

| Model information | Values |
| --- | --- |
| Number of observations | 864 |
| Number of participants | 12 |
| ICC | 0.346 |

| Fixed effects | Estimate | Lower 95% CI | Upper 95% CI | t | p |
| --- | --- | --- | --- | --- | --- |
| (Intercept) | 0.040 | 0.034 | 0.046 | 13.390 | <0.001 |
| LT | -0.005 | -0.012 | 0.001 | -1.583 | 1.000 |
| FS | -0.002 | -0.010 | 0.005 | -0.562 | 1.000 |

| Random effects | Estimate | Lower 95% CI | Upper 95% CI |
| --- | --- | --- | --- |
| (Intercept) | 0.007 | 0.004 | 0.013 |
| Error | 0.041 | 0.039 | 0.043 |

Reference group: LT

| Fixed effects | Estimate | Lower 95% CI | Upper 95% CI | t | p |
| --- | --- | --- | --- | --- | --- |
| (Intercept) | 0.035 | 0.028 | 0.041 | 9.985 | <0.001 |
| UA | 0.005 | -0.001 | 0.012 | 1.583 | 1.000 |
| FS | 0.003 | -0.005 | 0.011 | 0.781 | 1.000 |

| Random effects | Estimate | Lower 95% CI | Upper 95% CI |
| --- | --- | --- | --- |
| (Intercept) | 0.007 | 0.004 | 0.013 |
| Error | 0.041 | 0.039 | 0.043 |

**Detailed statistics: sit-to-stand, non-challenging condition, hybrid strategy, synergy 1**

Reference group: UA

| Model information | Values |
| --- | --- |
| Number of observations | 496 |
| Number of participants | 9 |
| ICC | 0.220 |

| Fixed effects | Estimate | Lower 95% CI | Upper 95% CI | t | p |
| --- | --- | --- | --- | --- | --- |
| (Intercept) | 0.029 | 0.024 | 0.035 | 9.978 | <0.001 |
| LT | 0.000 | -0.008 | 0.008 | 0.065 | 1.000 |
| FS | 0.011 | 0.001 | 0.021 | 2.192 | 0.346 |

| Random effects | Estimate | Lower 95% CI | Upper 95% CI |
| --- | --- | --- | --- |
| (Intercept) | 0.004 | 0.001 | 0.023 |
| Error | 0.037 | 0.035 | 0.040 |

Reference group: LT

| Fixed effects | Estimate | Lower 95% CI | Upper 95% CI | t | p |
| --- | --- | --- | --- | --- | --- |
| (Intercept) | 0.030 | 0.023 | 0.036 | 9.124 | <0.001 |
| UA | -0.000 | -0.008 | 0.008 | -0.065 | 1.000 |
| FS | 0.011 | 0.001 | 0.021 | 2.085 | 0.451 |

| Random effects | Estimate | Lower 95% CI | Upper 95% CI |
| --- | --- | --- | --- |
| (Intercept) | 0.004 | 0.001 | 0.023 |
| Error | 0.037 | 0.035 | 0.040 |

**Detailed statistics: sit-to-stand, non-challenging condition, hybrid strategy, synergy 3**

Reference group: UA

| Model information | Values |
| --- | --- |
| Number of observations | 496 |
| Number of participants | 9 |
| ICC | 0.703 |

| Fixed effects | Estimate | Lower 95% CI | Upper 95% CI | t | p |
| --- | --- | --- | --- | --- | --- |
| (Intercept) | 0.025 | 0.014 | 0.035 | 4.797 | <0.001 |
| LT | 0.022 | 0.014 | 0.031 | 5.026 | <0.001 |
| FS | 0.034 | 0.024 | 0.044 | 6.409 | <0.001 |

| Random effects | Estimate | Lower 95% CI | Upper 95% CI |
| --- | --- | --- | --- |
| (Intercept) | 0.013 | 0.007 | 0.023 |
| Error | 0.037 | 0.034 | 0.039 |

Reference group: LT

| Fixed effects | Estimate | Lower 95% CI | Upper 95% CI | t | p |
| --- | --- | --- | --- | --- | --- |
| (Intercept) | 0.047 | 0.036 | 0.057 | 8.616 | <0.001 |
| UA | -0.022 | -0.031 | -0.014 | -5.026 | <0.001 |
| FS | 0.012 | 0.001 | 0.023 | 2.118 | 0.416 |

| Random effects | Estimate | Lower 95% CI | Upper 95% CI |
| --- | --- | --- | --- |
| (Intercept) | 0.013 | 0.007 | 0.023 |
| Error | 0.037 | 0.034 | 0.039 |

Post-hoc UA vs. LT

| Fixed effects | Estimate | Lower 95% CI | Upper 95% CI | t | p |
| --- | --- | --- | --- | --- | --- |
| RA:LT | -0.005 | -0.027 | 0.017 | -0.429 | 1.000 |
| ES:LT | 0.007 | -0.014 | 0.029 | 0.672 | 1.000 |
| Pec:LT | -0.004 | -0.026 | 0.018 | -0.337 | 1.000 |
| Lat:LT | 0.023 | 0.002 | 0.045 | 2.108 | 0.071 |
| Tra:LT | 0.006 | -0.016 | 0.028 | 0.556 | 1.000 |
| Del:LT | -0.005 | -0.027 | 0.017 | -0.447 | 1.000 |
| Bic:LT | 0.015 | -0.007 | 0.036 | 1.309 | 0.383 |
| Tri:LT | -0.009 | -0.031 | 0.012 | -0.848 | 0.794 |
| GM:LT | -0.020 | -0.042 | 0.002 | -1.823 | 0.138 |
| TF:LT | -0.004 | -0.026 | 0.018 | -0.358 | 1.000 |
| RF:LT | 0.007 | -0.015 | 0.028 | 0.587 | 1.000 |
| VM:LT | 0.009 | -0.013 | 0.031 | 0.836 | 0.807 |
| BF:LT | -0.011 | -0.033 | 0.011 | -1.019 | 0.618 |
| TA:LT | 0.009 | -0.013 | 0.031 | 0.825 | 0.820 |
| PL:LT | 0.000 | -0.022 | 0.022 | 0.012 | 1.000 |

| Random effects | Estimate | Lower 95% CI | Upper 95% CI |
| --- | --- | --- | --- |
| (Intercept) | 0.014 | 0.008 | 0.023 |
| Error | 0.029 | 0.027 | 0.031 |

Post-hoc UA vs. FS

| Fixed effects | Estimate | Lower 95% CI | Upper 95% CI | t | p |
| --- | --- | --- | --- | --- | --- |
| RA:FS | -0.003 | -0.032 | 0.026 | -0.211 | 1.000 |
| ES:FS | 0.012 | -0.017 | 0.041 | 0.845 | 0.797 |
| Pec:FS | 0.014 | -0.015 | 0.043 | 0.960 | 0.676 |
| Lat:FS | 0.106 | 0.077 | 0.134 | 7.162 | <0.001 |
| Tra:FS | -0.004 | -0.033 | 0.025 | -0.254 | 1.000 |
| Del:FS | -0.038 | -0.067 | -0.009 | -2.576 | 0.021 |
| Bic:FS | 0.093 | 0.064 | 0.122 | 6.324 | <0.001 |
| Tri:FS | 0.027 | -0.002 | 0.056 | 1.861 | 0.127 |
| GM:FS | -0.046 | -0.075 | -0.017 | -3.125 | 0.004 |
| TF:FS | -0.031 | -0.060 | -0.002 | -2.122 | 0.069 |
| RF:FS | -0.015 | -0.043 | 0.014 | -0.985 | 0.650 |
| VM:FS | -0.006 | -0.034 | 0.023 | -0.375 | 1.000 |
| BF:FS | -0.031 | -0.060 | -0.002 | -2.117 | 0.070 |
| TA:FS | -0.012 | -0.040 | 0.017 | -0.783 | 0.869 |
| PL:FS | -0.029 | -0.058 | 0.000 | -1.964 | 0.100 |

| Random effects | Estimate | Lower 95% CI | Upper 95% CI |
| --- | --- | --- | --- |
| (Intercept) | 0.014 | 0.008 | 0.023 |
| Error | 0.029 | 0.027 | 0.031 |

**Detailed statistics: sit-to-stand, non-challenging condition, hybrid strategy, synergy 4**

Reference group: UA

| Model information | Values |
| --- | --- |
| Number of observations | 496 |
| Number of participants | 9 |
| ICC | 0.439 |

| Fixed effects | Estimate | Lower 95% CI | Upper 95% CI | t | p |
| --- | --- | --- | --- | --- | --- |
| (Intercept) | 0.046 | 0.038 | 0.054 | 11.567 | <0.001 |
| LT | -0.010 | -0.019 | -0.001 | -2.233 | 0.312 |
| FS | -0.012 | -0.023 | -0.001 | -2.235 | 0.310 |

| Random effects | Estimate | Lower 95% CI | Upper 95% CI |
| --- | --- | --- | --- |
| (Intercept) | 0.009 | 0.004 | 0.018 |
| Error | 0.039 | 0.036 | 0.041 |

Reference group: LT

| Fixed effects | Estimate | Lower 95% CI | Upper 95% CI | t | p |
| --- | --- | --- | --- | --- | --- |
| (Intercept) | 0.036 | 0.028 | 0.045 | 8.327 | <0.001 |
| UA | 0.010 | 0.001 | 0.019 | 2.233 | 0.312 |
| FS | -0.002 | -0.014 | 0.009 | -0.418 | 1.000 |

| Random effects | Estimate | Lower 95% CI | Upper 95% CI |
| --- | --- | --- | --- |
| (Intercept) | 0.009 | 0.004 | 0.018 |
| Error | 0.039 | 0.036 | 0.041 |

**Detailed statistics: sit-to-stand, non-challenging condition, hybrid strategy, synergy 5**

Reference group: UA

| Model information | Values |
| --- | --- |
| Number of observations | 496 |
| Number of participants | 9 |
| ICC | 0.527 |

| Fixed effects | Estimate | Lower 95% CI | Upper 95% CI | t | p |
| --- | --- | --- | --- | --- | --- |
| (Intercept) | 0.045 | 0.035 | 0.055 | 9.198 | <0.001 |
| LT | 0.003 | -0.006 | 0.013 | 0.679 | 1.000 |
| FS | 0.000 | -0.012 | 0.012 | 0.070 | 1.000 |

| Random effects | Estimate | Lower 95% CI | Upper 95% CI |
| --- | --- | --- | --- |
| (Intercept) | 0.011 | 0.006 | 0.023 |
| Error | 0.043 | 0.040 | 0.046 |

Reference group: LT

| Fixed effects | Estimate | Lower 95% CI | Upper 95% CI | t | p |
| --- | --- | --- | --- | --- | --- |
| (Intercept) | 0.049 | 0.038 | 0.059 | 9.145 | <0.001 |
| UA | -0.003 | -0.013 | 0.006 | -0.679 | 1.000 |
| FS | -0.003 | -0.015 | 0.010 | -0.468 | 1.000 |

| Random effects | Estimate | Lower 95% CI | Upper 95% CI |
| --- | --- | --- | --- |
| (Intercept) | 0.011 | 0.006 | 0.023 |
| Error | 0.043 | 0.040 | 0.046 |

**Detailed statistics: sit-to-stand, non-challenging condition, hybrid strategy, synergy 7**

Reference group: UA

| Model information | Values |
| --- | --- |
| Number of observations | 496 |
| Number of participants | 9 |
| ICC | 0.760 |

| Fixed effects | Estimate | Lower 95% CI | Upper 95% CI | t | p |
| --- | --- | --- | --- | --- | --- |
| (Intercept) | 0.035 | 0.023 | 0.047 | 5.701 | <0.001 |
| LT | 0.012 | 0.002 | 0.022 | 2.284 | 0.273 |
| FS | 0.005 | -0.007 | 0.017 | 0.799 | 1.000 |

| Random effects | Estimate | Lower 95% CI | Upper 95% CI |
| --- | --- | --- | --- |
| (Intercept) | 0.016 | 0.009 | 0.027 |
| Error | 0.042 | 0.039 | 0.045 |

Reference group: LT

| Fixed effects | Estimate | Lower 95% CI | Upper 95% CI | t | p |
| --- | --- | --- | --- | --- | --- |
| (Intercept) | 0.047 | 0.034 | 0.060 | 7.175 | <0.001 |
| UA | -0.012 | -0.022 | -0.002 | -2.284 | 0.273 |
| FS | -0.007 | -0.019 | 0.006 | -1.072 | 1.000 |

| Random effects | Estimate | Lower 95% CI | Upper 95% CI |
| --- | --- | --- | --- |
| (Intercept) | 0.016 | 0.009 | 0.027 |
| Error | 0.042 | 0.039 | 0.045 |

**Detailed statistics: sit-to-stand, non-challenging condition, hybrid strategy, synergy 8**

Reference group: UA

| Model information | Values |
| --- | --- |
| Number of observations | 496 |
| Number of participants | 9 |
| ICC | 0.304 |

| Fixed effects | Estimate | Lower 95% CI | Upper 95% CI | t | p |
| --- | --- | --- | --- | --- | --- |
| (Intercept) | 0.045 | 0.037 | 0.054 | 10.664 | <0.001 |
| LT | -0.005 | -0.015 | 0.006 | -0.888 | 1.000 |
| FS | -0.002 | -0.015 | 0.012 | -0.234 | 1.000 |

| Random effects | Estimate | Lower 95% CI | Upper 95% CI |
| --- | --- | --- | --- |
| (Intercept) | 0.007 | 0.003 | 0.018 |
| Error | 0.049 | 0.046 | 0.052 |

Reference group: LT

| Fixed effects | Estimate | Lower 95% CI | Upper 95% CI | t | p |
| --- | --- | --- | --- | --- | --- |
| (Intercept) | 0.040 | 0.031 | 0.050 | 8.655 | <0.001 |
| UA | 0.005 | -0.006 | 0.015 | 0.888 | 1.000 |
| FS | 0.003 | -0.011 | 0.017 | 0.449 | 1.000 |

| Random effects | Estimate | Lower 95% CI | Upper 95% CI |
| --- | --- | --- | --- |
| (Intercept) | 0.007 | 0.003 | 0.018 |
| Error | 0.049 | 0.046 | 0.052 |

**Detailed statistics: sit-to-stand, non-challenging condition, vertical rise strategy, synergy 1**

Reference group: UA

| Model information | Values |
| --- | --- |
| Number of observations | 1344 |
| Number of participants | 18 |
| ICC | 0.424 |

| Fixed effects | Estimate | Lower 95% CI | Upper 95% CI | t | p |
| --- | --- | --- | --- | --- | --- |
| (Intercept) | 0.026 | 0.020 | 0.032 | 8.712 | <0.001 |
| LT | 0.005 | -0.001 | 0.010 | 1.516 | 1.000 |
| FS | 0.010 | 0.005 | 0.016 | 3.599 | 0.004 |

| Random effects | Estimate | Lower 95% CI | Upper 95% CI |
| --- | --- | --- | --- |
| (Intercept) | 0.008 | 0.005 | 0.013 |
| Error | 0.038 | 0.036 | 0.039 |

Reference group: LT

| Fixed effects | Estimate | Lower 95% CI | Upper 95% CI | t | p |
| --- | --- | --- | --- | --- | --- |
| (Intercept) | 0.031 | 0.025 | 0.036 | 10.804 | <0.001 |
| UA | -0.005 | -0.010 | 0.001 | -1.516 | 1.000 |
| FS | 0.006 | 0.001 | 0.011 | 2.259 | 0.288 |

| Random effects | Estimate | Lower 95% CI | Upper 95% CI |
| --- | --- | --- | --- |
| (Intercept) | 0.008 | 0.005 | 0.013 |
| Error | 0.038 | 0.036 | 0.039 |

Post-hoc UA vs. FS

| Fixed effects | Estimate | Lower 95% CI | Upper 95% CI | t | p |
| --- | --- | --- | --- | --- | --- |
| RA:FS | -0.039 | -0.054 | -0.024 | -4.976 | <0.001 |
| ES:FS | -0.001 | -0.017 | 0.014 | -0.188 | 0.851 |
| Pec:FS | 0.024 | 0.009 | 0.039 | 3.080 | 0.002 |
| Lat:FS | 0.015 | -0.000 | 0.031 | 1.947 | 0.052 |
| Tra:FS | 0.086 | 0.071 | 0.101 | 10.973 | <0.001 |
| Del:FS | -0.013 | -0.029 | 0.002 | -1.721 | 0.086 |
| Bic:FS | 0.027 | 0.012 | 0.042 | 3.437 | 0.001 |
| Tri:FS | 0.010 | -0.005 | 0.026 | 1.299 | 0.194 |
| GM:FS | -0.023 | -0.038 | -0.008 | -2.951 | 0.003 |
| TF:FS | -0.022 | -0.037 | -0.006 | -2.789 | 0.005 |
| RF:FS | -0.007 | -0.022 | 0.008 | -0.904 | 0.366 |
| VM:FS | -0.007 | -0.022 | 0.009 | -0.840 | 0.401 |
| BF:FS | -0.007 | -0.022 | 0.009 | -0.860 | 0.390 |
| TA:FS | -0.017 | -0.033 | -0.002 | -2.234 | 0.026 |
| PL:FS | -0.016 | -0.031 | -0.001 | -2.047 | 0.041 |

| Random effects | Estimate | Lower 95% CI | Upper 95% CI |
| --- | --- | --- | --- |
| (Intercept) | 0.009 | 0.006 | 0.013 |
| Error | 0.029 | 0.028 | 0.030 |

**Detailed statistics: sit-to-stand, non-challenging condition, vertical rise strategy, synergy 2**

Reference group: UA

| Model information | Values |
| --- | --- |
| Number of observations | 1344 |
| Number of participants | 18 |
| ICC | 0.827 |

| Fixed effects | Estimate | Lower 95% CI | Upper 95% CI | t | p |
| --- | --- | --- | --- | --- | --- |
| (Intercept) | 0.020 | 0.012 | 0.028 | 4.845 | <0.001 |
| LT | 0.016 | 0.011 | 0.022 | 5.655 | <0.001 |
| FS | 0.025 | 0.020 | 0.031 | 9.042 | <0.001 |

| Random effects | Estimate | Lower 95% CI | Upper 95% CI |
| --- | --- | --- | --- |
| (Intercept) | 0.015 | 0.010 | 0.021 |
| Error | 0.036 | 0.034 | 0.037 |

Reference group: LT

| Fixed effects | Estimate | Lower 95% CI | Upper 95% CI | t | p |
| --- | --- | --- | --- | --- | --- |
| (Intercept) | 0.036 | 0.028 | 0.044 | 8.988 | <0.001 |
| UA | -0.016 | -0.022 | -0.011 | -5.655 | <0.001 |
| FS | 0.009 | 0.004 | 0.014 | 3.644 | 0.003 |

| Random effects | Estimate | Lower 95% CI | Upper 95% CI |
| --- | --- | --- | --- |
| (Intercept) | 0.015 | 0.010 | 0.021 |
| Error | 0.036 | 0.034 | 0.037 |

Post-hoc UA vs. LT

| Fixed effects | Estimate | Lower 95% CI | Upper 95% CI | t | p |
| --- | --- | --- | --- | --- | --- |
| RA:LT | -0.008 | -0.026 | 0.009 | -0.924 | 1.000 |
| ES:LT | 0.007 | -0.010 | 0.025 | 0.803 | 1.000 |
| Pec:LT | -0.021 | -0.038 | -0.003 | -2.347 | 0.057 |
| Lat:LT | -0.001 | -0.018 | 0.017 | -0.068 | 1.000 |
| Tra:LT | 0.023 | 0.006 | 0.041 | 2.583 | 0.030 |
| Del:LT | 0.004 | -0.013 | 0.022 | 0.491 | 1.000 |
| Bic:LT | 0.020 | 0.002 | 0.037 | 2.224 | 0.079 |
| Tri:LT | 0.007 | -0.011 | 0.024 | 0.759 | 1.000 |
| GM:LT | -0.012 | -0.030 | 0.005 | -1.386 | 0.498 |
| TF:LT | -0.002 | -0.019 | 0.016 | -0.220 | 1.000 |
| RF:LT | 0.010 | -0.007 | 0.028 | 1.153 | 0.747 |
| VM:LT | 0.005 | -0.012 | 0.023 | 0.569 | 1.000 |
| BF:LT | -0.007 | -0.025 | 0.010 | -0.820 | 1.000 |
| TA:LT | 0.002 | -0.016 | 0.019 | 0.193 | 1.000 |
| PL:LT | -0.012 | -0.030 | 0.005 | -1.365 | 0.517 |

| Random effects | Estimate | Lower 95% CI | Upper 95% CI |
| --- | --- | --- | --- |
| (Intercept) | 0.015 | 0.010 | 0.021 |
| Error | 0.031 | 0.030 | 0.032 |

Post-hoc UA vs. FS

| Fixed effects | Estimate | Lower 95% CI | Upper 95% CI | t | p |
| --- | --- | --- | --- | --- | --- |
| RA:FS | -0.017 | -0.033 | -0.001 | -2.038 | 0.125 |
| ES:FS | -0.004 | -0.020 | 0.012 | -0.496 | 1.000 |
| Pec:FS | -0.012 | -0.029 | 0.004 | -1.493 | 0.407 |
| Lat:FS | 0.028 | 0.012 | 0.044 | 3.356 | 0.002 |
| Tra:FS | 0.001 | -0.015 | 0.017 | 0.100 | 1.000 |
| Del:FS | -0.024 | -0.040 | -0.008 | -2.881 | 0.012 |
| Bic:FS | 0.027 | 0.011 | 0.043 | 3.289 | 0.003 |
| Tri:FS | 0.007 | -0.009 | 0.023 | 0.853 | 1.000 |
| GM:FS | -0.016 | -0.032 | 0.001 | -1.886 | 0.179 |
| TF:FS | 0.008 | -0.009 | 0.024 | 0.915 | 1.000 |
| RF:FS | 0.026 | 0.010 | 0.042 | 3.115 | 0.006 |
| VM:FS | 0.033 | 0.017 | 0.049 | 3.984 | <0.001 |
| BF:FS | -0.010 | -0.026 | 0.006 | -1.217 | 0.672 |
| TA:FS | -0.001 | -0.018 | 0.015 | -0.163 | 1.000 |
| PL:FS | -0.021 | -0.037 | -0.005 | -2.565 | 0.031 |

| Random effects | Estimate | Lower 95% CI | Upper 95% CI |
| --- | --- | --- | --- |
| (Intercept) | 0.015 | 0.010 | 0.021 |
| Error | 0.031 | 0.030 | 0.032 |

Post-hoc: LT vs. FS

| Fixed effects | Estimate | Lower 95% CI | Upper 95% CI | t | p |
| --- | --- | --- | --- | --- | --- |
| RA:FS | -0.009 | -0.024 | 0.006 | -1.133 | 0.773 |
| ES:FS | -0.011 | -0.026 | 0.004 | -1.477 | 0.420 |
| Pec:FS | 0.009 | -0.006 | 0.024 | 1.122 | 0.787 |
| Lat:FS | 0.028 | 0.013 | 0.043 | 3.722 | 0.001 |
| Tra:FS | -0.022 | -0.037 | -0.007 | -2.910 | 0.011 |
| Del:FS | -0.028 | -0.043 | -0.013 | -3.701 | 0.001 |
| Bic:FS | 0.007 | -0.008 | 0.022 | 0.971 | 0.996 |
| Tri:FS | 0.000 | -0.015 | 0.015 | 0.039 | 1.000 |
| GM:FS | -0.003 | -0.018 | 0.012 | -0.428 | 1.000 |
| TF:FS | 0.010 | -0.005 | 0.025 | 1.251 | 0.633 |
| RF:FS | 0.016 | 0.001 | 0.030 | 2.033 | 0.127 |
| VM:FS | 0.028 | 0.013 | 0.043 | 3.660 | 0.001 |
| BF:FS | -0.003 | -0.018 | 0.012 | -0.362 | 1.000 |
| TA:FS | -0.003 | -0.018 | 0.012 | -0.402 | 1.000 |
| PL:FS | -0.009 | -0.024 | 0.006 | -1.189 | 0.704 |

| Random effects | Estimate | Lower 95% CI | Upper 95% CI |
| --- | --- | --- | --- |
| (Intercept) | 0.015 | 0.010 | 0.021 |
| Error | 0.031 | 0.030 | 0.032 |

**Detailed statistics: sit-to-stand, non-challenging condition, vertical rise strategy, synergy 4**

Reference group: UA

| Model information | Values |
| --- | --- |
| Number of observations | 1344 |
| Number of participants | 18 |
| ICC | 0.353 |

| Fixed effects | Estimate | Lower 95% CI | Upper 95% CI | t | p |
| --- | --- | --- | --- | --- | --- |
| (Intercept) | 0.037 | 0.031 | 0.043 | 11.650 | <0.001 |
| LT | 0.013 | 0.006 | 0.020 | 3.884 | 0.001 |
| FS | 0.004 | -0.003 | 0.010 | 1.139 | 1.000 |

| Random effects | Estimate | Lower 95% CI | Upper 95% CI |
| --- | --- | --- | --- |
| (Intercept) | 0.008 | 0.005 | 0.012 |
| Error | 0.043 | 0.041 | 0.044 |

Reference group: LT

| Fixed effects | Estimate | Lower 95% CI | Upper 95% CI | t | p |
| --- | --- | --- | --- | --- | --- |
| (Intercept) | 0.050 | 0.044 | 0.056 | 16.823 | <0.001 |
| UA | -0.013 | -0.020 | -0.006 | -3.884 | 0.001 |
| FS | -0.009 | -0.015 | -0.004 | -3.280 | 0.013 |

| Random effects | Estimate | Lower 95% CI | Upper 95% CI |
| --- | --- | --- | --- |
| (Intercept) | 0.008 | 0.005 | 0.012 |
| Error | 0.043 | 0.041 | 0.044 |

Post-hoc UA vs. LT

| Fixed effects | Estimate | Lower 95% CI | Upper 95% CI | t | p |
| --- | --- | --- | --- | --- | --- |
| RA:LT | -0.007 | -0.025 | 0.011 | -0.757 | 0.899 |
| ES:LT | -0.024 | -0.042 | -0.006 | -2.660 | 0.016 |
| Pec:LT | -0.006 | -0.024 | 0.012 | -0.655 | 1.000 |
| Lat:LT | -0.001 | -0.019 | 0.017 | -0.118 | 1.000 |
| Tra:LT | -0.006 | -0.024 | 0.012 | -0.683 | 0.989 |
| Del:LT | 0.016 | -0.002 | 0.034 | 1.792 | 0.147 |
| Bic:LT | 0.003 | -0.015 | 0.021 | 0.319 | 1.000 |
| Tri:LT | -0.001 | -0.019 | 0.017 | -0.150 | 1.000 |
| GM:LT | 0.000 | -0.018 | 0.018 | 0.019 | 1.000 |
| TF:LT | 0.013 | -0.005 | 0.031 | 1.458 | 0.290 |
| RF:LT | 0.017 | -0.001 | 0.035 | 1.886 | 0.119 |
| VM:LT | 0.010 | -0.008 | 0.028 | 1.053 | 0.586 |
| BF:LT | 0.004 | -0.014 | 0.022 | 0.401 | 1.000 |
| TA:LT | 0.004 | -0.014 | 0.022 | 0.472 | 1.000 |
| PL:LT | -0.010 | -0.028 | 0.008 | -1.119 | 0.526 |

| Random effects | Estimate | Lower 95% CI | Upper 95% CI |
| --- | --- | --- | --- |
| (Intercept) | 0.008 | 0.005 | 0.012 |
| Error | 0.032 | 0.031 | 0.033 |

Post-hoc: LT vs. FS

| Fixed effects | Estimate | Lower 95% CI | Upper 95% CI | t | p |
| --- | --- | --- | --- | --- | --- |
| RA:FS | -0.010 | -0.025 | 0.006 | -1.249 | 0.424 |
| ES:FS | -0.031 | -0.047 | -0.016 | -4.014 | <0.001 |
| Pec:FS | 0.018 | 0.003 | 0.033 | 2.289 | 0.044 |
| Lat:FS | 0.032 | 0.017 | 0.047 | 4.094 | <0.001 |
| Tra:FS | 0.005 | -0.010 | 0.021 | 0.695 | 0.974 |
| Del:FS | -0.010 | -0.025 | 0.006 | -1.245 | 0.427 |
| Bic:FS | 0.020 | 0.005 | 0.036 | 2.599 | 0.019 |
| Tri:FS | 0.010 | -0.006 | 0.025 | 1.229 | 0.438 |
| GM:FS | -0.007 | -0.022 | 0.009 | -0.864 | 0.775 |
| TF:FS | -0.007 | -0.022 | 0.008 | -0.880 | 0.758 |
| RF:FS | -0.007 | -0.022 | 0.008 | -0.903 | 0.733 |
| VM:FS | 0.002 | -0.013 | 0.018 | 0.271 | 1.000 |
| BF:FS | 0.005 | -0.010 | 0.021 | 0.675 | 1.000 |
| TA:FS | -0.020 | -0.035 | -0.005 | -2.562 | 0.021 |
| PL:FS | -0.005 | -0.021 | 0.010 | -0.687 | 0.985 |

| Random effects | Estimate | Lower 95% CI | Upper 95% CI |
| --- | --- | --- | --- |
| (Intercept) | 0.008 | 0.005 | 0.012 |
| Error | 0.032 | 0.031 | 0.033 |

**Detailed statistics: sit-to-stand, non-challenging condition, vertical rise strategy, synergy 5**

Reference group: UA

| Model information | Values |
| --- | --- |
| Number of observations | 1344 |
| Number of participants | 18 |
| ICC | 0.351 |

| Fixed effects | Estimate | Lower 95% CI | Upper 95% CI | t | p |
| --- | --- | --- | --- | --- | --- |
| (Intercept) | 0.058 | 0.051 | 0.065 | 15.893 | <0.001 |
| LT | -0.007 | -0.014 | 0.001 | -1.715 | 1.000 |
| FS | -0.013 | -0.020 | -0.005 | -3.432 | 0.007 |

| Random effects | Estimate | Lower 95% CI | Upper 95% CI |
| --- | --- | --- | --- |
| (Intercept) | 0.009 | 0.005 | 0.014 |
| Error | 0.049 | 0.047 | 0.051 |

Reference group: LT

| Fixed effects | Estimate | Lower 95% CI | Upper 95% CI | t | p |
| --- | --- | --- | --- | --- | --- |
| (Intercept) | 0.051 | 0.045 | 0.058 | 15.032 | <0.001 |
| UA | 0.007 | -0.001 | 0.014 | 1.715 | 1.000 |
| FS | -0.006 | -0.012 | 0.000 | -1.820 | 0.827 |

| Random effects | Estimate | Lower 95% CI | Upper 95% CI |
| --- | --- | --- | --- |
| (Intercept) | 0.009 | 0.005 | 0.014 |
| Error | 0.049 | 0.047 | 0.051 |

Post-hoc UA vs. FS

| Fixed effects | Estimate | Lower 95% CI | Upper 95% CI | t | p |
| --- | --- | --- | --- | --- | --- |
| RA:FS | -0.003 | -0.020 | 0.014 | -0.370 | 0.711 |
| ES:FS | -0.026 | -0.043 | -0.009 | -3.065 | 0.002 |
| Pec:FS | 0.036 | 0.020 | 0.053 | 4.259 | <0.001 |
| Lat:FS | 0.081 | 0.064 | 0.097 | 9.414 | <0.001 |
| Tra:FS | 0.014 | -0.003 | 0.031 | 1.631 | 0.103 |
| Del:FS | 0.050 | 0.033 | 0.067 | 5.824 | <0.001 |
| Bic:FS | 0.017 | 0.000 | 0.034 | 2.003 | 0.045 |
| Tri:FS | 0.046 | 0.029 | 0.063 | 5.401 | <0.001 |
| GM:FS | -0.010 | -0.027 | 0.006 | -1.211 | 0.226 |
| TF:FS | -0.045 | -0.062 | -0.028 | -5.240 | <0.001 |
| RF:FS | -0.064 | -0.080 | -0.047 | -7.427 | <0.001 |
| VM:FS | -0.054 | -0.071 | -0.037 | -6.311 | <0.001 |
| BF:FS | 0.003 | -0.013 | 0.020 | 0.392 | 0.695 |
| TA:FS | -0.034 | -0.051 | -0.018 | -4.014 | <0.001 |
| PL:FS | -0.015 | -0.032 | 0.002 | -1.757 | 0.079 |

| Random effects | Estimate | Lower 95% CI | Upper 95% CI |
| --- | --- | --- | --- |
| (Intercept) | 0.010 | 0.007 | 0.015 |
| Error | 0.032 | 0.031 | 0.033 |

**Detailed statistics: sit-to-stand, non-challenging condition, vertical rise strategy, synergy 7**

Reference group: UA

| Model information | Values |
| --- | --- |
| Number of observations | 1344 |
| Number of participants | 18 |
| ICC | 0.197 |

| Fixed effects | Estimate | Lower 95% CI | Upper 95% CI | t | p |
| --- | --- | --- | --- | --- | --- |
| (Intercept) | 0.036 | 0.030 | 0.041 | 12.193 | <0.001 |
| LT | 0.007 | 0.000 | 0.014 | 1.988 | 0.564 |
| FS | 0.013 | 0.007 | 0.020 | 3.966 | 0.001 |

| Random effects | Estimate | Lower 95% CI | Upper 95% CI |
| --- | --- | --- | --- |
| (Intercept) | 0.005 | 0.002 | 0.010 |
| Error | 0.046 | 0.044 | 0.048 |

Reference group: LT

| Fixed effects | Estimate | Lower 95% CI | Upper 95% CI | t | p |
| --- | --- | --- | --- | --- | --- |
| (Intercept) | 0.043 | 0.037 | 0.048 | 16.051 | <0.001 |
| UA | -0.007 | -0.014 | -0.000 | -1.988 | 0.564 |
| FS | 0.006 | 0.000 | 0.012 | 2.037 | 0.502 |

| Random effects | Estimate | Lower 95% CI | Upper 95% CI |
| --- | --- | --- | --- |
| (Intercept) | 0.005 | 0.002 | 0.010 |
| Error | 0.046 | 0.044 | 0.048 |

Post-hoc UA vs. FS

| Fixed effects | Estimate | Lower 95% CI | Upper 95% CI | t | p |
| --- | --- | --- | --- | --- | --- |
| RA:FS | -0.015 | -0.035 | 0.005 | -1.477 | 0.140 |
| ES:FS | -0.038 | -0.058 | -0.018 | -3.716 | <0.001 |
| Pec:FS | 0.008 | -0.012 | 0.028 | 0.818 | 0.414 |
| Lat:FS | 0.021 | 0.001 | 0.041 | 2.081 | 0.038 |
| Tra:FS | -0.011 | -0.031 | 0.008 | -1.131 | 0.258 |
| Del:FS | 0.035 | 0.015 | 0.055 | 3.441 | 0.001 |
| Bic:FS | -0.005 | -0.025 | 0.015 | -0.527 | 0.598 |
| Tri:FS | 0.080 | 0.061 | 0.100 | 7.946 | <0.001 |
| GM:FS | 0.007 | -0.013 | 0.027 | 0.721 | 0.471 |
| TF:FS | -0.003 | -0.023 | 0.017 | -0.332 | 0.740 |
| RF:FS | 0.005 | -0.015 | 0.025 | 0.495 | 0.620 |
| VM:FS | -0.001 | -0.021 | 0.019 | -0.090 | 0.928 |
| BF:FS | -0.019 | -0.039 | 0.001 | -1.848 | 0.065 |
| TA:FS | -0.020 | -0.040 | -0.001 | -2.015 | 0.044 |
| PL:FS | -0.026 | -0.046 | -0.006 | -2.579 | 0.010 |

| Random effects | Estimate | Lower 95% CI | Upper 95% CI |
| --- | --- | --- | --- |
| (Intercept) | 0.005 | 0.003 | 0.010 |
| Error | 0.038 | 0.036 | 0.039 |

**Detailed statistics: sit-to-stand, non-challenging condition, vertical rise strategy, synergy 8**

Reference group: UA

| Model information | Values |
| --- | --- |
| Number of observations | 1344 |
| Number of participants | 18 |
| ICC | 0.338 |

| Fixed effects | Estimate | Lower 95% CI | Upper 95% CI | t | p |
| --- | --- | --- | --- | --- | --- |
| (Intercept) | 0.049 | 0.042 | 0.056 | 13.873 | <0.001 |
| LT | -0.003 | -0.010 | 0.004 | -0.787 | 1.000 |
| FS | -0.005 | -0.013 | 0.002 | -1.515 | 1.000 |

| Random effects | Estimate | Lower 95% CI | Upper 95% CI |
| --- | --- | --- | --- |
| (Intercept) | 0.008 | 0.005 | 0.014 |
| Error | 0.048 | 0.047 | 0.050 |

Reference group: LT

| Fixed effects | Estimate | Lower 95% CI | Upper 95% CI | t | p |
| --- | --- | --- | --- | --- | --- |
| (Intercept) | 0.046 | 0.040 | 0.053 | 13.941 | <0.001 |
| UA | 0.003 | -0.004 | 0.010 | 0.787 | 1.000 |
| FS | -0.002 | -0.009 | 0.004 | -0.766 | 1.000 |

| Random effects | Estimate | Lower 95% CI | Upper 95% CI |
| --- | --- | --- | --- |
| (Intercept) | 0.008 | 0.005 | 0.014 |
| Error | 0.048 | 0.047 | 0.050 |

## Sit-to-stand, challenging condition

**Detailed statistics: sit-to-stand, challenging condition, exaggerated forward leaning strategy, synergy 2**

Reference group: UA

| Model information | Values |
| --- | --- |
| Number of observations | 1152 |
| Number of participants | 19 |
| ICC | 0.458 |

| Fixed effects | Estimate | Lower 95% CI | Upper 95% CI | t | p |
| --- | --- | --- | --- | --- | --- |
| (Intercept) | 0.029 | 0.024 | 0.034 | 11.991 | <0.001 |
| LT | 0.011 | 0.005 | 0.016 | 3.853 | 0.002 |
| FS | 0.015 | 0.007 | 0.023 | 3.834 | 0.003 |

| Random effects | Estimate | Lower 95% CI | Upper 95% CI |
| --- | --- | --- | --- |
| (Intercept) | 0.008 | 0.006 | 0.013 |
| Error | 0.037 | 0.035 | 0.038 |

Reference group: LT

| Fixed effects | Estimate | Lower 95% CI | Upper 95% CI | t | p |
| --- | --- | --- | --- | --- | --- |
| (Intercept) | 0.040 | 0.034 | 0.046 | 13.020 | <0.001 |
| UA | -0.011 | -0.016 | -0.005 | -3.853 | 0.002 |
| FS | 0.004 | -0.003 | 0.012 | 1.091 | 1.000 |

| Random effects | Estimate | Lower 95% CI | Upper 95% CI |
| --- | --- | --- | --- |
| (Intercept) | 0.008 | 0.006 | 0.013 |
| Error | 0.037 | 0.035 | 0.038 |

Post-hoc UA vs. LT

| Fixed effects | Estimate | Lower 95% CI | Upper 95% CI | t | p |
| --- | --- | --- | --- | --- | --- |
| RA:LT | -0.009 | -0.023 | 0.004 | -1.387 | 0.331 |
| ES:LT | 0.010 | -0.003 | 0.023 | 1.467 | 0.285 |
| Pec:LT | -0.022 | -0.036 | -0.009 | -3.251 | 0.002 |
| Lat:LT | -0.004 | -0.018 | 0.009 | -0.608 | 1.000 |
| Tra:LT | 0.047 | 0.033 | 0.060 | 6.854 | <0.001 |
| Del:LT | 0.001 | -0.012 | 0.015 | 0.165 | 1.000 |
| Bic:LT | 0.012 | -0.001 | 0.026 | 1.798 | 0.145 |
| Tri:LT | 0.009 | -0.005 | 0.022 | 1.254 | 0.420 |
| GM:LT | -0.015 | -0.028 | -0.002 | -2.186 | 0.058 |
| TF:LT | -0.012 | -0.025 | 0.001 | -1.753 | 0.160 |
| RF:LT | -0.005 | -0.018 | 0.009 | -0.697 | 0.972 |
| VM:LT | -0.010 | -0.023 | 0.003 | -1.467 | 0.285 |
| BF:LT | -0.008 | -0.022 | 0.005 | -1.209 | 0.454 |
| TA:LT | 0.012 | -0.001 | 0.026 | 1.814 | 0.140 |
| PL:LT | 0.001 | -0.013 | 0.014 | 0.103 | 1.000 |

| Random effects | Estimate | Lower 95% CI | Upper 95% CI |
| --- | --- | --- | --- |
| (Intercept) | 0.009 | 0.006 | 0.013 |
| Error | 0.026 | 0.025 | 0.027 |

Post-hoc UA vs. FS

| Fixed effects | Estimate | Lower 95% CI | Upper 95% CI | t | p |
| --- | --- | --- | --- | --- | --- |
| RA:FS | -0.005 | -0.023 | 0.013 | -0.541 | 1.000 |
| ES:FS | -0.014 | -0.032 | 0.004 | -1.506 | 0.265 |
| Pec:FS | 0.013 | -0.006 | 0.031 | 1.362 | 0.347 |
| Lat:FS | 0.011 | -0.007 | 0.029 | 1.171 | 0.484 |
| Tra:FS | 0.076 | 0.058 | 0.094 | 8.219 | <0.001 |
| Del:FS | -0.046 | -0.064 | -0.027 | -4.937 | <0.001 |
| Bic:FS | 0.067 | 0.048 | 0.085 | 7.207 | <0.001 |
| Tri:FS | 0.010 | -0.008 | 0.028 | 1.052 | 0.586 |
| GM:FS | -0.021 | -0.039 | -0.003 | -2.242 | 0.050 |
| TF:FS | -0.016 | -0.034 | 0.002 | -1.768 | 0.155 |
| RF:FS | -0.006 | -0.024 | 0.012 | -0.687 | 0.985 |
| VM:FS | -0.008 | -0.027 | 0.010 | -0.918 | 0.717 |
| BF:FS | -0.008 | -0.026 | 0.010 | -0.880 | 0.758 |
| TA:FS | -0.026 | -0.044 | -0.008 | -2.783 | 0.011 |
| PL:FS | -0.016 | -0.034 | 0.002 | -1.717 | 0.172 |

| Random effects | Estimate | Lower 95% CI | Upper 95% CI |
| --- | --- | --- | --- |
| (Intercept) | 0.009 | 0.006 | 0.013 |
| Error | 0.026 | 0.025 | 0.027 |

**Detailed statistics: sit-to-stand, challenging condition, exaggerated forward leaning strategy, synergy 3**

Reference group: UA

| Model information | Values |
| --- | --- |
| Number of observations | 1152 |
| Number of participants | 19 |
| ICC | 0.437 |

| Fixed effects | Estimate | Lower 95% CI | Upper 95% CI | t | p |
| --- | --- | --- | --- | --- | --- |
| (Intercept) | 0.032 | 0.027 | 0.036 | 14.212 | <0.001 |
| LT | 0.007 | 0.002 | 0.012 | 2.573 | 0.204 |
| FS | -0.002 | -0.009 | 0.005 | -0.521 | 1.000 |

| Random effects | Estimate | Lower 95% CI | Upper 95% CI |
| --- | --- | --- | --- |
| (Intercept) | 0.008 | 0.005 | 0.012 |
| Error | 0.035 | 0.033 | 0.036 |

Reference group: LT

| Fixed effects | Estimate | Lower 95% CI | Upper 95% CI | t | p |
| --- | --- | --- | --- | --- | --- |
| (Intercept) | 0.038 | 0.033 | 0.044 | 13.535 | <0.001 |
| UA | -0.007 | -0.012 | -0.002 | -2.573 | 0.204 |
| FS | -0.009 | -0.016 | -0.001 | -2.331 | 0.398 |

| Random effects | Estimate | Lower 95% CI | Upper 95% CI |
| --- | --- | --- | --- |
| (Intercept) | 0.008 | 0.005 | 0.012 |
| Error | 0.035 | 0.033 | 0.036 |

**Detailed statistics: sit-to-stand, challenging condition, exaggerated forward leaning strategy, synergy 4**

Reference group: UA

| Model information | Values |
| --- | --- |
| Number of observations | 1152 |
| Number of participants | 19 |
| ICC | 0.482 |

| Fixed effects | Estimate | Lower 95% CI | Upper 95% CI | t | p |
| --- | --- | --- | --- | --- | --- |
| (Intercept) | 0.034 | 0.029 | 0.038 | 15.385 | <0.001 |
| LT | 0.002 | -0.003 | 0.007 | 0.901 | 1.000 |
| FS | -0.003 | -0.010 | 0.004 | -0.761 | 1.000 |

| Random effects | Estimate | Lower 95% CI | Upper 95% CI |
| --- | --- | --- | --- |
| (Intercept) | 0.008 | 0.005 | 0.012 |
| Error | 0.032 | 0.031 | 0.034 |

Reference group: LT

| Fixed effects | Estimate | Lower 95% CI | Upper 95% CI | t | p |
| --- | --- | --- | --- | --- | --- |
| (Intercept) | 0.036 | 0.031 | 0.041 | 13.089 | <0.001 |
| UA | -0.002 | -0.007 | 0.003 | -0.901 | 1.000 |
| FS | -0.005 | -0.012 | 0.002 | -1.396 | 1.000 |

| Random effects | Estimate | Lower 95% CI | Upper 95% CI |
| --- | --- | --- | --- |
| (Intercept) | 0.008 | 0.005 | 0.012 |
| Error | 0.032 | 0.031 | 0.034 |

**Detailed statistics: sit-to-stand, challenging condition, exaggerated forward leaning strategy, synergy 5**

Reference group: UA

| Model information | Values |
| --- | --- |
| Number of observations | 1152 |
| Number of participants | 19 |
| ICC | 0.607 |

| Fixed effects | Estimate | Lower 95% CI | Upper 95% CI | t | p |
| --- | --- | --- | --- | --- | --- |
| (Intercept) | 0.038 | 0.033 | 0.042 | 15.178 | <0.001 |
| LT | -0.009 | -0.014 | -0.005 | -3.879 | 0.002 |
| FS | -0.012 | -0.019 | -0.006 | -3.619 | 0.006 |

| Random effects | Estimate | Lower 95% CI | Upper 95% CI |
| --- | --- | --- | --- |
| (Intercept) | 0.009 | 0.006 | 0.014 |
| Error | 0.031 | 0.030 | 0.032 |

Reference group: LT

| Fixed effects | Estimate | Lower 95% CI | Upper 95% CI | t | p |
| --- | --- | --- | --- | --- | --- |
| (Intercept) | 0.028 | 0.022 | 0.034 | 9.526 | <0.001 |
| UA | 0.009 | 0.005 | 0.014 | 3.879 | 0.002 |
| FS | -0.003 | -0.010 | 0.004 | -0.867 | 1.000 |

| Random effects | Estimate | Lower 95% CI | Upper 95% CI |
| --- | --- | --- | --- |
| (Intercept) | 0.009 | 0.006 | 0.014 |
| Error | 0.031 | 0.030 | 0.032 |

Post-hoc UA vs. LT

| Fixed effects | Estimate | Lower 95% CI | Upper 95% CI | t | p |
| --- | --- | --- | --- | --- | --- |
| RA:LT | -0.003 | -0.015 | 0.010 | -0.408 | 1.000 |
| ES:LT | -0.017 | -0.030 | -0.005 | -2.657 | 0.016 |
| Pec:LT | 0.004 | -0.009 | 0.017 | 0.617 | 1.000 |
| Lat:LT | 0.006 | -0.007 | 0.019 | 0.877 | 0.761 |
| Tra:LT | -0.001 | -0.014 | 0.012 | -0.203 | 1.000 |
| Del:LT | 0.034 | 0.021 | 0.047 | 5.198 | <0.001 |
| Bic:LT | 0.019 | 0.006 | 0.032 | 2.890 | 0.008 |
| Tri:LT | 0.028 | 0.015 | 0.041 | 4.242 | <0.001 |
| GM:LT | -0.010 | -0.023 | 0.003 | -1.572 | 0.232 |
| TF:LT | -0.026 | -0.039 | -0.013 | -3.988 | <0.001 |
| RF:LT | -0.014 | -0.027 | -0.002 | -2.205 | 0.055 |
| VM:LT | -0.006 | -0.019 | 0.006 | -0.994 | 0.641 |
| BF:LT | -0.008 | -0.021 | 0.005 | -1.217 | 0.448 |
| TA:LT | -0.002 | -0.015 | 0.011 | -0.329 | 1.000 |
| PL:LT | -0.004 | -0.017 | 0.009 | -0.605 | 1.000 |

| Random effects | Estimate | Lower 95% CI | Upper 95% CI |
| --- | --- | --- | --- |
| (Intercept) | 0.010 | 0.007 | 0.015 |
| Error | 0.025 | 0.024 | 0.026 |

Post-hoc UA vs. FS

| Fixed effects | Estimate | Lower 95% CI | Upper 95% CI | t | p |
| --- | --- | --- | --- | --- | --- |
| RA:FS | 0.009 | -0.008 | 0.026 | 1.017 | 0.619 |
| ES:FS | -0.022 | -0.039 | -0.004 | -2.446 | 0.029 |
| Pec:FS | 0.017 | -0.000 | 0.035 | 1.945 | 0.104 |
| Lat:FS | 0.019 | 0.002 | 0.036 | 2.136 | 0.066 |
| Tra:FS | 0.003 | -0.014 | 0.021 | 0.385 | 1.000 |
| Del:FS | 0.017 | -0.001 | 0.034 | 1.884 | 0.120 |
| Bic:FS | 0.017 | -0.000 | 0.035 | 1.935 | 0.107 |
| Tri:FS | 0.033 | 0.015 | 0.050 | 3.687 | <0.001 |
| GM:FS | -0.005 | -0.022 | 0.012 | -0.561 | 1.000 |
| TF:FS | -0.034 | -0.052 | -0.017 | -3.870 | <0.001 |
| RF:FS | -0.017 | -0.035 | 0.000 | -1.945 | 0.104 |
| VM:FS | -0.011 | -0.028 | 0.007 | -1.191 | 0.467 |
| BF:FS | -0.003 | -0.021 | 0.014 | -0.356 | 1.000 |
| TA:FS | -0.018 | -0.035 | -0.001 | -2.031 | 0.085 |
| PL:FS | -0.010 | -0.027 | 0.007 | -1.143 | 0.507 |

| Random effects | Estimate | Lower 95% CI | Upper 95% CI |
| --- | --- | --- | --- |
| (Intercept) | 0.010 | 0.007 | 0.015 |
| Error | 0.025 | 0.024 | 0.026 |

**Detailed statistics: sit-to-stand, challenging condition, exaggerated forward leaning strategy, synergy 6**

Reference group: UA

| Model information | Values |
| --- | --- |
| Number of observations | 1152 |
| Number of participants | 19 |
| ICC | 0.449 |

| Fixed effects | Estimate | Lower 95% CI | Upper 95% CI | t | p |
| --- | --- | --- | --- | --- | --- |
| (Intercept) | 0.031 | 0.028 | 0.035 | 17.801 | <0.001 |
| LT | -0.009 | -0.013 | -0.005 | -4.493 | <0.001 |
| FS | -0.005 | -0.010 | 0.001 | -1.557 | 1.000 |

| Random effects | Estimate | Lower 95% CI | Upper 95% CI |
| --- | --- | --- | --- |
| (Intercept) | 0.006 | 0.004 | 0.010 |
| Error | 0.027 | 0.026 | 0.028 |

Reference group: LT

| Fixed effects | Estimate | Lower 95% CI | Upper 95% CI | t | p |
| --- | --- | --- | --- | --- | --- |
| (Intercept) | 0.022 | 0.017 | 0.026 | 9.841 | <0.001 |
| UA | 0.009 | 0.005 | 0.013 | 4.493 | <0.001 |
| FS | 0.005 | -0.001 | 0.010 | 1.624 | 1.000 |

| Random effects | Estimate | Lower 95% CI | Upper 95% CI |
| --- | --- | --- | --- |
| (Intercept) | 0.006 | 0.004 | 0.010 |
| Error | 0.027 | 0.026 | 0.028 |

Post-hoc UA vs. LT

| Fixed effects | Estimate | Lower 95% CI | Upper 95% CI | t | p |
| --- | --- | --- | --- | --- | --- |
| RA:LT | 0.001 | -0.011 | 0.014 | 0.208 | 0.835 |
| ES:LT | -0.005 | -0.018 | 0.007 | -0.833 | 0.405 |
| Pec:LT | -0.001 | -0.014 | 0.011 | -0.184 | 0.854 |
| Lat:LT | 0.011 | -0.001 | 0.024 | 1.773 | 0.077 |
| Tra:LT | 0.005 | -0.007 | 0.018 | 0.805 | 0.421 |
| Del:LT | 0.018 | 0.006 | 0.030 | 2.837 | 0.005 |
| Bic:LT | 0.009 | -0.004 | 0.021 | 1.380 | 0.168 |
| Tri:LT | 0.026 | 0.013 | 0.038 | 4.045 | <0.001 |
| GM:LT | -0.012 | -0.025 | 0.000 | -1.938 | 0.053 |
| TF:LT | -0.010 | -0.023 | 0.002 | -1.633 | 0.103 |
| RF:LT | -0.005 | -0.017 | 0.007 | -0.790 | 0.430 |
| VM:LT | 0.007 | -0.005 | 0.020 | 1.159 | 0.247 |
| BF:LT | 0.000 | -0.012 | 0.013 | 0.044 | 0.965 |
| TA:LT | -0.022 | -0.035 | -0.010 | -3.509 | <0.001 |
| PL:LT | -0.018 | -0.030 | -0.005 | -2.807 | 0.005 |

| Random effects | Estimate | Lower 95% CI | Upper 95% CI |
| --- | --- | --- | --- |
| (Intercept) | 0.006 | 0.004 | 0.010 |
| Error | 0.024 | 0.023 | 0.025 |

**Detailed statistics: sit-to-stand, challenging condition, exaggerated forward leaning strategy, synergy 7**

Reference group: UA

| Model information | Values |
| --- | --- |
| Number of observations | 1152 |
| Number of participants | 19 |
| ICC | 0.473 |

| Fixed effects | Estimate | Lower 95% CI | Upper 95% CI | t | p |
| --- | --- | --- | --- | --- | --- |
| (Intercept) | 0.035 | 0.031 | 0.039 | 16.607 | <0.001 |
| LT | -0.016 | -0.021 | -0.012 | -6.769 | <0.001 |
| FS | -0.016 | -0.023 | -0.010 | -4.829 | <0.001 |

| Random effects | Estimate | Lower 95% CI | Upper 95% CI |
| --- | --- | --- | --- |
| (Intercept) | 0.007 | 0.005 | 0.012 |
| Error | 0.031 | 0.030 | 0.033 |

Reference group: LT

| Fixed effects | Estimate | Lower 95% CI | Upper 95% CI | t | p |
| --- | --- | --- | --- | --- | --- |
| (Intercept) | 0.019 | 0.014 | 0.024 | 7.047 | <0.001 |
| UA | 0.016 | 0.012 | 0.021 | 6.769 | <0.001 |
| FS | -0.000 | -0.007 | 0.007 | -0.021 | 1.000 |

| Random effects | Estimate | Lower 95% CI | Upper 95% CI |
| --- | --- | --- | --- |
| (Intercept) | 0.007 | 0.005 | 0.012 |
| Error | 0.031 | 0.030 | 0.033 |

Post-hoc UA vs. LT

| Fixed effects | Estimate | Lower 95% CI | Upper 95% CI | t | p |
| --- | --- | --- | --- | --- | --- |
| RA:LT | 0.003 | -0.011 | 0.017 | 0.457 | 1.000 |
| ES:LT | -0.013 | -0.027 | 0.001 | -1.768 | 0.155 |
| Pec:LT | 0.007 | -0.007 | 0.022 | 1.029 | 0.607 |
| Lat:LT | 0.012 | -0.002 | 0.026 | 1.657 | 0.196 |
| Tra:LT | 0.011 | -0.003 | 0.025 | 1.545 | 0.245 |
| Del:LT | 0.023 | 0.009 | 0.037 | 3.234 | 0.003 |
| Bic:LT | 0.016 | 0.002 | 0.031 | 2.283 | 0.045 |
| Tri:LT | 0.043 | 0.029 | 0.057 | 5.992 | <0.001 |
| GM:LT | 0.001 | -0.013 | 0.015 | 0.130 | 1.000 |
| TF:LT | -0.016 | -0.030 | -0.002 | -2.236 | 0.051 |
| RF:LT | -0.010 | -0.024 | 0.005 | -1.335 | 0.365 |
| VM:LT | -0.002 | -0.016 | 0.012 | -0.238 | 1.000 |
| BF:LT | -0.005 | -0.019 | 0.009 | -0.686 | 0.986 |
| TA:LT | -0.024 | -0.038 | -0.010 | -3.365 | 0.002 |
| PL:LT | -0.028 | -0.042 | -0.014 | -3.849 | <0.001 |

| Random effects | Estimate | Lower 95% CI | Upper 95% CI |
| --- | --- | --- | --- |
| (Intercept) | 0.008 | 0.005 | 0.012 |
| Error | 0.027 | 0.026 | 0.029 |

Post-hoc UA vs. FS

| Fixed effects | Estimate | Lower 95% CI | Upper 95% CI | t | p |
| --- | --- | --- | --- | --- | --- |
| RA:FS | 0.002 | -0.017 | 0.022 | 0.252 | 1.000 |
| ES:FS | -0.026 | -0.045 | -0.007 | -2.647 | 0.016 |
| Pec:FS | 0.021 | 0.002 | 0.040 | 2.163 | 0.061 |
| Lat:FS | 0.018 | -0.001 | 0.037 | 1.864 | 0.125 |
| Tra:FS | 0.009 | -0.010 | 0.028 | 0.895 | 0.742 |
| Del:FS | 0.028 | 0.009 | 0.047 | 2.839 | 0.009 |
| Bic:FS | 0.017 | -0.002 | 0.036 | 1.748 | 0.162 |
| Tri:FS | 0.060 | 0.041 | 0.080 | 6.210 | <0.001 |
| GM:FS | -0.007 | -0.026 | 0.012 | -0.720 | 0.943 |
| TF:FS | -0.019 | -0.038 | -0.000 | -1.981 | 0.096 |
| RF:FS | -0.013 | -0.032 | 0.006 | -1.371 | 0.342 |
| VM:FS | -0.004 | -0.023 | 0.015 | -0.378 | 1.000 |
| BF:FS | -0.005 | -0.024 | 0.014 | -0.490 | 1.000 |
| TA:FS | -0.023 | -0.042 | -0.004 | -2.393 | 0.034 |
| PL:FS | -0.032 | -0.051 | -0.013 | -3.248 | 0.002 |

| Random effects | Estimate | Lower 95% CI | Upper 95% CI |
| --- | --- | --- | --- |
| (Intercept) | 0.008 | 0.005 | 0.012 |
| Error | 0.027 | 0.026 | 0.029 |

**Detailed statistics: sit-to-stand, challenging condition, exaggerated forward leaning strategy, synergy 8**

Reference group: UA

| Model information | Values |
| --- | --- |
| Number of observations | 1152 |
| Number of participants | 19 |
| ICC | 0.355 |

| Fixed effects | Estimate | Lower 95% CI | Upper 95% CI | t | p |
| --- | --- | --- | --- | --- | --- |
| (Intercept) | 0.032 | 0.029 | 0.035 | 20.775 | <0.001 |
| LT | -0.015 | -0.019 | -0.011 | -7.143 | <0.001 |
| FS | -0.016 | -0.021 | -0.010 | -5.428 | <0.001 |

| Random effects | Estimate | Lower 95% CI | Upper 95% CI |
| --- | --- | --- | --- |
| (Intercept) | 0.005 | 0.003 | 0.008 |
| Error | 0.027 | 0.026 | 0.028 |

Reference group: LT

| Fixed effects | Estimate | Lower 95% CI | Upper 95% CI | t | p |
| --- | --- | --- | --- | --- | --- |
| (Intercept) | 0.017 | 0.013 | 0.021 | 8.527 | <0.001 |
| UA | 0.015 | 0.011 | 0.019 | 7.143 | <0.001 |
| FS | -0.001 | -0.007 | 0.005 | -0.345 | 1.000 |

| Random effects | Estimate | Lower 95% CI | Upper 95% CI |
| --- | --- | --- | --- |
| (Intercept) | 0.005 | 0.003 | 0.008 |
| Error | 0.027 | 0.026 | 0.028 |

Post-hoc UA vs. LT

| Fixed effects | Estimate | Lower 95% CI | Upper 95% CI | t | p |
| --- | --- | --- | --- | --- | --- |
| RA:LT | 0.003 | -0.009 | 0.016 | 0.502 | 1.000 |
| ES:LT | -0.011 | -0.023 | 0.002 | -1.672 | 0.189 |
| Pec:LT | 0.003 | -0.009 | 0.016 | 0.535 | 1.000 |
| Lat:LT | 0.005 | -0.007 | 0.017 | 0.780 | 0.871 |
| Tra:LT | 0.011 | -0.001 | 0.024 | 1.771 | 0.154 |
| Del:LT | 0.021 | 0.009 | 0.034 | 3.364 | 0.002 |
| Bic:LT | 0.013 | 0.001 | 0.025 | 2.076 | 0.076 |
| Tri:LT | 0.039 | 0.027 | 0.051 | 6.180 | <0.001 |
| GM:LT | 0.001 | -0.011 | 0.013 | 0.163 | 1.000 |
| TF:LT | -0.008 | -0.020 | 0.004 | -1.250 | 0.423 |
| RF:LT | -0.012 | -0.025 | 0.000 | -1.934 | 0.107 |
| VM:LT | 0.001 | -0.012 | 0.013 | 0.131 | 1.000 |
| BF:LT | -0.007 | -0.020 | 0.005 | -1.158 | 0.495 |
| TA:LT | -0.010 | -0.023 | 0.002 | -1.633 | 0.206 |
| PL:LT | -0.030 | -0.043 | -0.018 | -4.819 | <0.001 |

| Random effects | Estimate | Lower 95% CI | Upper 95% CI |
| --- | --- | --- | --- |
| (Intercept) | 0.005 | 0.003 | 0.008 |
| Error | 0.024 | 0.023 | 0.025 |

Post-hoc UA vs. FS

| Fixed effects | Estimate | Lower 95% CI | Upper 95% CI | t | p |
| --- | --- | --- | --- | --- | --- |
| RA:FS | 0.011 | -0.006 | 0.028 | 1.316 | 0.377 |
| ES:FS | -0.009 | -0.026 | 0.007 | -1.089 | 0.553 |
| Pec:FS | 0.010 | -0.007 | 0.027 | 1.169 | 0.486 |
| Lat:FS | 0.008 | -0.009 | 0.024 | 0.906 | 0.730 |
| Tra:FS | 0.015 | -0.001 | 0.032 | 1.787 | 0.148 |
| Del:FS | 0.013 | -0.004 | 0.029 | 1.487 | 0.275 |
| Bic:FS | 0.020 | 0.003 | 0.037 | 2.325 | 0.041 |
| Tri:FS | 0.039 | 0.022 | 0.056 | 4.588 | <0.001 |
| GM:FS | -0.003 | -0.020 | 0.013 | -0.409 | 1.000 |
| TF:FS | -0.017 | -0.034 | -0.000 | -1.974 | 0.097 |
| RF:FS | -0.003 | -0.019 | 0.014 | -0.313 | 1.000 |
| VM:FS | -0.002 | -0.019 | 0.014 | -0.281 | 1.000 |
| BF:FS | -0.002 | -0.019 | 0.014 | -0.263 | 1.000 |
| TA:FS | -0.013 | -0.030 | 0.004 | -1.536 | 0.250 |
| PL:FS | -0.040 | -0.056 | -0.023 | -4.663 | <0.001 |

| Random effects | Estimate | Lower 95% CI | Upper 95% CI |
| --- | --- | --- | --- |
| (Intercept) | 0.005 | 0.003 | 0.008 |
| Error | 0.024 | 0.023 | 0.025 |

**Detailed statistics: sit-to-stand, challenging condition, exaggerated forward leaning strategy, synergy 9**

Reference group: UA

| Model information | Values |
| --- | --- |
| Number of observations | 1152 |
| Number of participants | 19 |
| ICC | 0.253 |

| Fixed effects | Estimate | Lower 95% CI | Upper 95% CI | t | p |
| --- | --- | --- | --- | --- | --- |
| (Intercept) | 0.039 | 0.036 | 0.042 | 23.596 | <0.001 |
| LT | -0.018 | -0.023 | -0.014 | -7.429 | <0.001 |
| FS | -0.014 | -0.021 | -0.007 | -4.024 | 0.001 |

| Random effects | Estimate | Lower 95% CI | Upper 95% CI |
| --- | --- | --- | --- |
| (Intercept) | 0.004 | 0.002 | 0.009 |
| Error | 0.034 | 0.033 | 0.036 |

Reference group: LT

| Fixed effects | Estimate | Lower 95% CI | Upper 95% CI | t | p |
| --- | --- | --- | --- | --- | --- |
| (Intercept) | 0.021 | 0.016 | 0.025 | 8.973 | <0.001 |
| UA | 0.018 | 0.014 | 0.023 | 7.429 | <0.001 |
| FS | 0.004 | -0.003 | 0.012 | 1.232 | 1.000 |

| Random effects | Estimate | Lower 95% CI | Upper 95% CI |
| --- | --- | --- | --- |
| (Intercept) | 0.004 | 0.002 | 0.009 |
| Error | 0.034 | 0.033 | 0.036 |

Post-hoc UA vs. LT

| Fixed effects | Estimate | Lower 95% CI | Upper 95% CI | t | p |
| --- | --- | --- | --- | --- | --- |
| RA:LT | 0.009 | -0.006 | 0.024 | 1.161 | 0.492 |
| ES:LT | -0.003 | -0.018 | 0.012 | -0.440 | 1.000 |
| Pec:LT | 0.009 | -0.006 | 0.024 | 1.189 | 0.469 |
| Lat:LT | 0.009 | -0.006 | 0.024 | 1.211 | 0.453 |
| Tra:LT | 0.016 | 0.001 | 0.031 | 2.121 | 0.068 |
| Del:LT | 0.013 | -0.002 | 0.028 | 1.746 | 0.162 |
| Bic:LT | 0.012 | -0.003 | 0.027 | 1.628 | 0.208 |
| Tri:LT | 0.047 | 0.032 | 0.062 | 6.201 | <0.001 |
| GM:LT | -0.005 | -0.019 | 0.010 | -0.600 | 1.000 |
| TF:LT | -0.006 | -0.021 | 0.008 | -0.853 | 0.788 |
| RF:LT | -0.002 | -0.016 | 0.013 | -0.205 | 1.000 |
| VM:LT | 0.010 | -0.005 | 0.025 | 1.337 | 0.363 |
| BF:LT | -0.016 | -0.031 | -0.001 | -2.060 | 0.079 |
| TA:LT | -0.017 | -0.032 | -0.003 | -2.299 | 0.043 |
| PL:LT | -0.044 | -0.059 | -0.029 | -5.784 | <0.001 |

| Random effects | Estimate | Lower 95% CI | Upper 95% CI |
| --- | --- | --- | --- |
| (Intercept) | 0.005 | 0.003 | 0.009 |
| Error | 0.029 | 0.028 | 0.030 |

Post-hoc UA vs. FS

| Fixed effects | Estimate | Lower 95% CI | Upper 95% CI | t | p |
| --- | --- | --- | --- | --- | --- |
| RA:FS | 0.014 | -0.007 | 0.034 | 1.327 | 0.370 |
| ES:FS | -0.011 | -0.032 | 0.009 | -1.101 | 0.542 |
| Pec:FS | 0.042 | 0.022 | 0.062 | 4.050 | <0.001 |
| Lat:FS | 0.014 | -0.006 | 0.034 | 1.363 | 0.346 |
| Tra:FS | 0.019 | -0.002 | 0.039 | 1.811 | 0.141 |
| Del:FS | 0.015 | -0.005 | 0.035 | 1.448 | 0.296 |
| Bic:FS | 0.027 | 0.006 | 0.047 | 2.589 | 0.020 |
| Tri:FS | 0.050 | 0.030 | 0.070 | 4.861 | <0.001 |
| GM:FS | -0.006 | -0.026 | 0.014 | -0.574 | 1.000 |
| TF:FS | -0.010 | -0.031 | 0.010 | -1.018 | 0.617 |
| RF:FS | -0.008 | -0.028 | 0.012 | -0.756 | 0.899 |
| VM:FS | 0.000 | -0.020 | 0.020 | 0.021 | 1.000 |
| BF:FS | -0.038 | -0.058 | -0.018 | -3.711 | <0.001 |
| TA:FS | -0.022 | -0.042 | -0.002 | -2.135 | 0.066 |
| PL:FS | -0.048 | -0.068 | -0.027 | -4.625 | <0.001 |

| Random effects | Estimate | Lower 95% CI | Upper 95% CI |
| --- | --- | --- | --- |
| (Intercept) | 0.005 | 0.003 | 0.009 |
| Error | 0.029 | 0.028 | 0.030 |

**Detailed statistics: sit-to-stand, challenging condition, exaggerated forward leaning strategy, synergy 10**

Reference group: UA

| Model information | Values |
| --- | --- |
| Number of observations | 1152 |
| Number of participants | 19 |
| ICC | 0.210 |

| Fixed effects | Estimate | Lower 95% CI | Upper 95% CI | t | p |
| --- | --- | --- | --- | --- | --- |
| (Intercept) | 0.032 | 0.030 | 0.035 | 24.172 | <0.001 |
| LT | -0.014 | -0.018 | -0.009 | -6.465 | <0.001 |
| FS | -0.011 | -0.017 | -0.006 | -3.914 | 0.002 |

| Random effects | Estimate | Lower 95% CI | Upper 95% CI |
| --- | --- | --- | --- |
| (Intercept) | 0.003 | 0.001 | 0.008 |
| Error | 0.029 | 0.028 | 0.031 |

Reference group: LT

| Fixed effects | Estimate | Lower 95% CI | Upper 95% CI | t | p |
| --- | --- | --- | --- | --- | --- |
| (Intercept) | 0.019 | 0.015 | 0.022 | 9.915 | <0.001 |
| UA | 0.014 | 0.009 | 0.018 | 6.465 | <0.001 |
| FS | 0.002 | -0.004 | 0.008 | 0.685 | 1.000 |

| Random effects | Estimate | Lower 95% CI | Upper 95% CI |
| --- | --- | --- | --- |
| (Intercept) | 0.003 | 0.001 | 0.008 |
| Error | 0.029 | 0.028 | 0.031 |

Post-hoc UA vs. LT

| Fixed effects | Estimate | Lower 95% CI | Upper 95% CI | t | p |
| --- | --- | --- | --- | --- | --- |
| RA:LT | 0.006 | -0.007 | 0.019 | 0.929 | 0.706 |
| ES:LT | 0.004 | -0.009 | 0.016 | 0.553 | 1.000 |
| Pec:LT | 0.007 | -0.005 | 0.020 | 1.130 | 0.517 |
| Lat:LT | 0.003 | -0.010 | 0.015 | 0.411 | 1.000 |
| Tra:LT | 0.014 | 0.001 | 0.026 | 2.165 | 0.061 |
| Del:LT | 0.013 | 0.001 | 0.026 | 2.084 | 0.075 |
| Bic:LT | 0.010 | -0.003 | 0.022 | 1.515 | 0.260 |
| Tri:LT | 0.029 | 0.017 | 0.042 | 4.537 | <0.001 |
| GM:LT | -0.006 | -0.019 | 0.007 | -0.936 | 0.699 |
| TF:LT | -0.001 | -0.014 | 0.011 | -0.184 | 1.000 |
| RF:LT | 0.000 | -0.012 | 0.013 | 0.067 | 1.000 |
| VM:LT | 0.011 | -0.002 | 0.023 | 1.689 | 0.183 |
| BF:LT | -0.018 | -0.031 | -0.005 | -2.806 | 0.010 |
| TA:LT | -0.019 | -0.031 | -0.006 | -2.919 | 0.007 |
| PL:LT | -0.025 | -0.037 | -0.012 | -3.817 | <0.001 |

| Random effects | Estimate | Lower 95% CI | Upper 95% CI |
| --- | --- | --- | --- |
| (Intercept) | 0.004 | 0.002 | 0.007 |
| Error | 0.024 | 0.024 | 0.026 |

Post-hoc UA vs. FS

| Fixed effects | Estimate | Lower 95% CI | Upper 95% CI | t | p |
| --- | --- | --- | --- | --- | --- |
| RA:FS | 0.015 | -0.002 | 0.032 | 1.723 | 0.170 |
| ES:FS | 0.017 | -0.000 | 0.034 | 1.916 | 0.111 |
| Pec:FS | 0.034 | 0.017 | 0.051 | 3.959 | <0.001 |
| Lat:FS | 0.012 | -0.005 | 0.029 | 1.359 | 0.349 |
| Tra:FS | 0.015 | -0.002 | 0.032 | 1.738 | 0.165 |
| Del:FS | 0.018 | 0.001 | 0.035 | 2.032 | 0.085 |
| Bic:FS | 0.031 | 0.013 | 0.048 | 3.514 | 0.001 |
| Tri:FS | 0.032 | 0.015 | 0.049 | 3.708 | <0.001 |
| GM:FS | -0.007 | -0.024 | 0.010 | -0.783 | 0.867 |
| TF:FS | -0.010 | -0.028 | 0.007 | -1.203 | 0.458 |
| RF:FS | -0.011 | -0.028 | 0.006 | -1.304 | 0.385 |
| VM:FS | -0.010 | -0.027 | 0.007 | -1.161 | 0.492 |
| BF:FS | -0.035 | -0.052 | -0.018 | -4.024 | <0.001 |
| TA:FS | -0.021 | -0.038 | -0.003 | -2.364 | 0.037 |
| PL:FS | -0.035 | -0.052 | -0.018 | -4.063 | <0.001 |

| Random effects | Estimate | Lower 95% CI | Upper 95% CI |
| --- | --- | --- | --- |
| (Intercept) | 0.004 | 0.002 | 0.007 |
| Error | 0.024 | 0.024 | 0.026 |

**Detailed statistics: sit-to-stand, challenging condition, exaggerated forward leaning strategy, synergy 11**

Reference group: UA

| Model information | Values |
| --- | --- |
| Number of observations | 1152 |
| Number of participants | 19 |
| ICC | 0.211 |

| Fixed effects | Estimate | Lower 95% CI | Upper 95% CI | t | p |
| --- | --- | --- | --- | --- | --- |
| (Intercept) | 0.038 | 0.035 | 0.041 | 24.836 | <0.001 |
| LT | -0.015 | -0.019 | -0.010 | -6.246 | <0.001 |
| FS | -0.015 | -0.022 | -0.009 | -4.617 | <0.001 |

| Random effects | Estimate | Lower 95% CI | Upper 95% CI |
| --- | --- | --- | --- |
| (Intercept) | 0.003 | 0.001 | 0.008 |
| Error | 0.033 | 0.032 | 0.035 |

Reference group: LT

| Fixed effects | Estimate | Lower 95% CI | Upper 95% CI | t | p |
| --- | --- | --- | --- | --- | --- |
| (Intercept) | 0.023 | 0.019 | 0.027 | 10.644 | <0.001 |
| UA | 0.015 | 0.010 | 0.019 | 6.246 | <0.001 |
| FS | -0.000 | -0.007 | 0.006 | -0.137 | 1.000 |

| Random effects | Estimate | Lower 95% CI | Upper 95% CI |
| --- | --- | --- | --- |
| (Intercept) | 0.003 | 0.001 | 0.008 |
| Error | 0.033 | 0.032 | 0.035 |

Post-hoc UA vs. LT

| Fixed effects | Estimate | Lower 95% CI | Upper 95% CI | t | p |
| --- | --- | --- | --- | --- | --- |
| RA:LT | 0.015 | 0.001 | 0.028 | 2.153 | 0.063 |
| ES:LT | -0.004 | -0.018 | 0.009 | -0.652 | 1.000 |
| Pec:LT | 0.011 | -0.003 | 0.024 | 1.533 | 0.251 |
| Lat:LT | 0.009 | -0.005 | 0.022 | 1.288 | 0.396 |
| Tra:LT | 0.013 | -0.001 | 0.027 | 1.885 | 0.119 |
| Del:LT | 0.009 | -0.005 | 0.022 | 1.274 | 0.406 |
| Bic:LT | 0.009 | -0.004 | 0.023 | 1.346 | 0.357 |
| Tri:LT | 0.027 | 0.014 | 0.041 | 3.940 | <0.001 |
| GM:LT | -0.009 | -0.022 | 0.005 | -1.296 | 0.390 |
| TF:LT | 0.001 | -0.012 | 0.015 | 0.208 | 1.000 |
| RF:LT | -0.003 | -0.016 | 0.011 | -0.376 | 1.000 |
| VM:LT | 0.010 | -0.003 | 0.024 | 1.518 | 0.259 |
| BF:LT | -0.023 | -0.037 | -0.010 | -3.365 | 0.002 |
| TA:LT | -0.023 | -0.037 | -0.010 | -3.405 | 0.001 |
| PL:LT | -0.018 | -0.032 | -0.005 | -2.677 | 0.015 |

| Random effects | Estimate | Lower 95% CI | Upper 95% CI |
| --- | --- | --- | --- |
| (Intercept) | 0.005 | 0.003 | 0.008 |
| Error | 0.026 | 0.025 | 0.027 |

Post-hoc UA vs. FS

| Fixed effects | Estimate | Lower 95% CI | Upper 95% CI | t | p |
| --- | --- | --- | --- | --- | --- |
| RA:FS | 0.014 | -0.005 | 0.032 | 1.469 | 0.284 |
| ES:FS | 0.007 | -0.011 | 0.025 | 0.747 | 0.910 |
| Pec:FS | 0.027 | 0.009 | 0.046 | 2.921 | 0.007 |
| Lat:FS | 0.022 | 0.004 | 0.041 | 2.410 | 0.032 |
| Tra:FS | 0.019 | 0.000 | 0.037 | 1.996 | 0.092 |
| Del:FS | 0.004 | -0.014 | 0.023 | 0.473 | 1.000 |
| Bic:FS | 0.028 | 0.010 | 0.046 | 2.988 | 0.006 |
| Tri:FS | 0.025 | 0.007 | 0.043 | 2.685 | 0.015 |
| GM:FS | 0.000 | -0.018 | 0.018 | 0.002 | 1.000 |
| TF:FS | -0.003 | -0.021 | 0.015 | -0.337 | 1.000 |
| RF:FS | -0.010 | -0.029 | 0.008 | -1.100 | 0.544 |
| VM:FS | -0.004 | -0.022 | 0.015 | -0.378 | 1.000 |
| BF:FS | -0.034 | -0.052 | -0.016 | -3.647 | 0.001 |
| TA:FS | -0.025 | -0.043 | -0.006 | -2.633 | 0.017 |
| PL:FS | -0.027 | -0.045 | -0.009 | -2.901 | 0.008 |

| Random effects | Estimate | Lower 95% CI | Upper 95% CI |
| --- | --- | --- | --- |
| (Intercept) | 0.005 | 0.003 | 0.008 |
| Error | 0.026 | 0.025 | 0.027 |

**Detailed statistics: sit-to-stand, challenging condition, forward leaning strategy, synergy 1**

Reference group: UA

| Model information | Values |
| --- | --- |
| Number of observations | 1648 |
| Number of participants | 19 |
| ICC | 0.436 |

| Fixed effects | Estimate | Lower 95% CI | Upper 95% CI | t | p |
| --- | --- | --- | --- | --- | --- |
| (Intercept) | 0.021 | 0.015 | 0.027 | 6.954 | <0.001 |
| LT | 0.002 | -0.004 | 0.008 | 0.661 | 1.000 |
| FS | 0.012 | 0.006 | 0.017 | 4.257 | <0.001 |

| Random effects | Estimate | Lower 95% CI | Upper 95% CI |
| --- | --- | --- | --- |
| (Intercept) | 0.008 | 0.005 | 0.011 |
| Error | 0.035 | 0.034 | 0.036 |

Reference group: LT

| Fixed effects | Estimate | Lower 95% CI | Upper 95% CI | t | p |
| --- | --- | --- | --- | --- | --- |
| (Intercept) | 0.023 | 0.018 | 0.028 | 9.926 | <0.001 |
| UA | -0.002 | -0.008 | 0.004 | -0.661 | 1.000 |
| FS | 0.010 | 0.006 | 0.014 | 5.137 | <0.001 |

| Random effects | Estimate | Lower 95% CI | Upper 95% CI |
| --- | --- | --- | --- |
| (Intercept) | 0.008 | 0.005 | 0.011 |
| Error | 0.035 | 0.034 | 0.036 |

Post-hoc UA vs. FS

| Fixed effects | Estimate | Lower 95% CI | Upper 95% CI | t | p |
| --- | --- | --- | --- | --- | --- |
| RA:FS | -0.018 | -0.033 | -0.003 | -2.406 | 0.033 |
| ES:FS | -0.008 | -0.022 | 0.007 | -0.986 | 0.649 |
| Pec:FS | 0.018 | 0.003 | 0.033 | 2.389 | 0.034 |
| Lat:FS | -0.005 | -0.020 | 0.010 | -0.669 | 1.000 |
| Tra:FS | 0.067 | 0.053 | 0.082 | 8.864 | <0.001 |
| Del:FS | -0.023 | -0.038 | -0.008 | -3.044 | 0.005 |
| Bic:FS | 0.044 | 0.029 | 0.059 | 5.779 | <0.001 |
| Tri:FS | 0.019 | 0.004 | 0.034 | 2.456 | 0.028 |
| GM:FS | -0.034 | -0.049 | -0.019 | -4.455 | <0.001 |
| TF:FS | -0.012 | -0.026 | 0.003 | -1.519 | 0.258 |
| RF:FS | -0.007 | -0.022 | 0.008 | -0.913 | 0.723 |
| VM:FS | -0.006 | -0.021 | 0.009 | -0.843 | 0.799 |
| BF:FS | -0.019 | -0.034 | -0.004 | -2.516 | 0.024 |
| TA:FS | 0.005 | -0.010 | 0.020 | 0.651 | 1.000 |
| PL:FS | -0.005 | -0.020 | 0.010 | -0.649 | 1.000 |

| Random effects | Estimate | Lower 95% CI | Upper 95% CI |
| --- | --- | --- | --- |
| (Intercept) | 0.008 | 0.006 | 0.012 |
| Error | 0.027 | 0.026 | 0.028 |

Post-hoc: LT vs. FS

| Fixed effects | Estimate | Lower 95% CI | Upper 95% CI | t | p |
| --- | --- | --- | --- | --- | --- |
| RA:FS | -0.004 | -0.015 | 0.007 | -0.774 | 0.878 |
| ES:FS | 0.004 | -0.007 | 0.014 | 0.630 | 1.000 |
| Pec:FS | 0.016 | 0.005 | 0.027 | 2.836 | 0.009 |
| Lat:FS | 0.005 | -0.006 | 0.016 | 0.854 | 0.786 |
| Tra:FS | 0.018 | 0.007 | 0.029 | 3.293 | 0.002 |
| Del:FS | -0.034 | -0.045 | -0.023 | -6.064 | <0.001 |
| Bic:FS | 0.043 | 0.032 | 0.054 | 7.652 | <0.001 |
| Tri:FS | 0.016 | 0.005 | 0.027 | 2.940 | 0.007 |
| GM:FS | -0.016 | -0.027 | -0.005 | -2.819 | 0.010 |
| TF:FS | -0.008 | -0.019 | 0.003 | -1.474 | 0.281 |
| RF:FS | -0.002 | -0.013 | 0.009 | -0.379 | 1.000 |
| VM:FS | -0.003 | -0.014 | 0.008 | -0.510 | 1.000 |
| BF:FS | -0.011 | -0.022 | -0.000 | -2.031 | 0.085 |
| TA:FS | -0.008 | -0.019 | 0.003 | -1.357 | 0.350 |
| PL:FS | -0.008 | -0.019 | 0.003 | -1.418 | 0.313 |

| Random effects | Estimate | Lower 95% CI | Upper 95% CI |
| --- | --- | --- | --- |
| (Intercept) | 0.008 | 0.006 | 0.012 |
| Error | 0.027 | 0.026 | 0.028 |

**Detailed statistics: sit-to-stand, challenging condition, forward leaning strategy, synergy 2**

Reference group: UA

| Model information | Values |
| --- | --- |
| Number of observations | 1648 |
| Number of participants | 19 |
| ICC | 0.439 |

| Fixed effects | Estimate | Lower 95% CI | Upper 95% CI | t | p |
| --- | --- | --- | --- | --- | --- |
| (Intercept) | 0.023 | 0.016 | 0.029 | 6.983 | <0.001 |
| LT | 0.008 | 0.002 | 0.014 | 2.445 | 0.233 |
| FS | 0.015 | 0.009 | 0.021 | 5.093 | <0.001 |

| Random effects | Estimate | Lower 95% CI | Upper 95% CI |
| --- | --- | --- | --- |
| (Intercept) | 0.008 | 0.006 | 0.012 |
| Error | 0.038 | 0.036 | 0.039 |

Reference group: LT

| Fixed effects | Estimate | Lower 95% CI | Upper 95% CI | t | p |
| --- | --- | --- | --- | --- | --- |
| (Intercept) | 0.031 | 0.026 | 0.036 | 12.207 | <0.001 |
| UA | -0.008 | -0.014 | -0.002 | -2.445 | 0.233 |
| FS | 0.008 | 0.003 | 0.012 | 3.634 | 0.005 |

| Random effects | Estimate | Lower 95% CI | Upper 95% CI |
| --- | --- | --- | --- |
| (Intercept) | 0.008 | 0.006 | 0.012 |
| Error | 0.038 | 0.036 | 0.039 |

Post-hoc UA vs. FS

| Fixed effects | Estimate | Lower 95% CI | Upper 95% CI | t | p |
| --- | --- | --- | --- | --- | --- |
| RA:FS | -0.018 | -0.034 | -0.001 | -2.123 | 0.068 |
| ES:FS | 0.002 | -0.014 | 0.019 | 0.259 | 1.000 |
| Pec:FS | -0.010 | -0.027 | 0.006 | -1.247 | 0.425 |
| Lat:FS | 0.015 | -0.001 | 0.031 | 1.791 | 0.147 |
| Tra:FS | 0.015 | -0.002 | 0.031 | 1.767 | 0.155 |
| Del:FS | -0.029 | -0.045 | -0.013 | -3.472 | 0.001 |
| Bic:FS | 0.079 | 0.063 | 0.096 | 9.470 | <0.001 |
| Tri:FS | 0.028 | 0.012 | 0.045 | 3.371 | 0.002 |
| GM:FS | -0.019 | -0.035 | -0.002 | -2.253 | 0.049 |
| TF:FS | -0.003 | -0.019 | 0.014 | -0.316 | 1.000 |
| RF:FS | 0.007 | -0.009 | 0.024 | 0.875 | 0.763 |
| VM:FS | -0.002 | -0.018 | 0.015 | -0.208 | 1.000 |
| BF:FS | -0.011 | -0.028 | 0.005 | -1.354 | 0.352 |
| TA:FS | -0.019 | -0.035 | -0.002 | -2.258 | 0.048 |
| PL:FS | -0.016 | -0.033 | 0.000 | -1.929 | 0.108 |

| Random effects | Estimate | Lower 95% CI | Upper 95% CI |
| --- | --- | --- | --- |
| (Intercept) | 0.009 | 0.006 | 0.012 |
| Error | 0.029 | 0.028 | 0.030 |

Post-hoc: LT vs. FS

| Fixed effects | Estimate | Lower 95% CI | Upper 95% CI | t | p |
| --- | --- | --- | --- | --- | --- |
| RA:FS | -0.016 | -0.029 | -0.004 | -2.678 | 0.015 |
| ES:FS | 0.006 | -0.006 | 0.018 | 0.906 | 0.730 |
| Pec:FS | 0.003 | -0.009 | 0.015 | 0.531 | 1.000 |
| Lat:FS | 0.012 | 0.000 | 0.024 | 2.010 | 0.089 |
| Tra:FS | -0.016 | -0.028 | -0.004 | -2.626 | 0.017 |
| Del:FS | -0.025 | -0.037 | -0.013 | -4.062 | <0.001 |
| Bic:FS | 0.053 | 0.041 | 0.065 | 8.631 | <0.001 |
| Tri:FS | 0.017 | 0.005 | 0.029 | 2.795 | 0.010 |
| GM:FS | -0.002 | -0.014 | 0.010 | -0.374 | 1.000 |
| TF:FS | 0.006 | -0.006 | 0.018 | 0.938 | 0.697 |
| RF:FS | 0.010 | -0.003 | 0.022 | 1.555 | 0.240 |
| VM:FS | 0.008 | -0.004 | 0.020 | 1.298 | 0.389 |
| BF:FS | -0.002 | -0.014 | 0.010 | -0.390 | 1.000 |
| TA:FS | -0.024 | -0.036 | -0.012 | -3.930 | <0.001 |
| PL:FS | -0.016 | -0.028 | -0.004 | -2.611 | 0.018 |

| Random effects | Estimate | Lower 95% CI | Upper 95% CI |
| --- | --- | --- | --- |
| (Intercept) | 0.009 | 0.006 | 0.012 |
| Error | 0.029 | 0.028 | 0.030 |

**Detailed statistics: sit-to-stand, challenging condition, forward leaning strategy, synergy 3**

Reference group: UA

| Model information | Values |
| --- | --- |
| Number of observations | 1648 |
| Number of participants | 19 |
| ICC | 0.500 |

| Fixed effects | Estimate | Lower 95% CI | Upper 95% CI | t | p |
| --- | --- | --- | --- | --- | --- |
| (Intercept) | 0.038 | 0.031 | 0.044 | 10.893 | <0.001 |
| LT | 0.005 | -0.001 | 0.012 | 1.695 | 1.000 |
| FS | 0.003 | -0.003 | 0.009 | 1.019 | 1.000 |

| Random effects | Estimate | Lower 95% CI | Upper 95% CI |
| --- | --- | --- | --- |
| (Intercept) | 0.009 | 0.006 | 0.014 |
| Error | 0.038 | 0.036 | 0.039 |

Reference group: LT

| Fixed effects | Estimate | Lower 95% CI | Upper 95% CI | t | p |
| --- | --- | --- | --- | --- | --- |
| (Intercept) | 0.043 | 0.038 | 0.048 | 15.793 | <0.001 |
| UA | -0.005 | -0.012 | 0.001 | -1.695 | 1.000 |
| FS | -0.002 | -0.006 | 0.002 | -1.108 | 1.000 |

| Random effects | Estimate | Lower 95% CI | Upper 95% CI |
| --- | --- | --- | --- |
| (Intercept) | 0.009 | 0.006 | 0.014 |
| Error | 0.038 | 0.036 | 0.039 |

**Detailed statistics: sit-to-stand, challenging condition, forward leaning strategy, synergy 4**

Reference group: UA

| Model information | Values |
| --- | --- |
| Number of observations | 1648 |
| Number of participants | 19 |
| ICC | 0.284 |

| Fixed effects | Estimate | Lower 95% CI | Upper 95% CI | t | p |
| --- | --- | --- | --- | --- | --- |
| (Intercept) | 0.044 | 0.039 | 0.048 | 18.266 | <0.001 |
| LT | -0.008 | -0.013 | -0.003 | -3.230 | 0.020 |
| FS | -0.014 | -0.019 | -0.010 | -5.880 | <0.001 |

| Random effects | Estimate | Lower 95% CI | Upper 95% CI |
| --- | --- | --- | --- |
| (Intercept) | 0.004 | 0.003 | 0.007 |
| Error | 0.031 | 0.030 | 0.032 |

Reference group: LT

| Fixed effects | Estimate | Lower 95% CI | Upper 95% CI | t | p |
| --- | --- | --- | --- | --- | --- |
| (Intercept) | 0.035 | 0.032 | 0.039 | 21.006 | <0.001 |
| UA | 0.008 | 0.003 | 0.013 | 3.230 | 0.020 |
| FS | -0.006 | -0.009 | -0.003 | -3.531 | 0.007 |

| Random effects | Estimate | Lower 95% CI | Upper 95% CI |
| --- | --- | --- | --- |
| (Intercept) | 0.004 | 0.003 | 0.007 |
| Error | 0.031 | 0.030 | 0.032 |

Post-hoc UA vs. LT

| Fixed effects | Estimate | Lower 95% CI | Upper 95% CI | t | p |
| --- | --- | --- | --- | --- | --- |
| RA:LT | -0.001 | -0.017 | 0.015 | -0.067 | 1.000 |
| ES:LT | 0.016 | -0.000 | 0.032 | 1.920 | 0.165 |
| Pec:LT | 0.003 | -0.013 | 0.019 | 0.336 | 1.000 |
| Lat:LT | 0.008 | -0.008 | 0.024 | 0.983 | 0.977 |
| Tra:LT | -0.005 | -0.021 | 0.011 | -0.588 | 1.000 |
| Del:LT | 0.027 | 0.011 | 0.043 | 3.275 | 0.003 |
| Bic:LT | 0.015 | -0.001 | 0.031 | 1.818 | 0.208 |
| Tri:LT | 0.027 | 0.011 | 0.042 | 3.258 | 0.003 |
| GM:LT | -0.013 | -0.029 | 0.003 | -1.567 | 0.352 |
| TF:LT | -0.011 | -0.027 | 0.005 | -1.385 | 0.499 |
| RF:LT | 0.008 | -0.008 | 0.024 | 1.008 | 0.940 |
| VM:LT | -0.001 | -0.016 | 0.015 | -0.062 | 1.000 |
| BF:LT | -0.006 | -0.022 | 0.010 | -0.749 | 1.000 |
| TA:LT | -0.016 | -0.032 | 0.000 | -1.955 | 0.152 |
| PL:LT | -0.034 | -0.050 | -0.018 | -4.186 | <0.001 |

| Random effects | Estimate | Lower 95% CI | Upper 95% CI |
| --- | --- | --- | --- |
| (Intercept) | 0.005 | 0.003 | 0.007 |
| Error | 0.027 | 0.027 | 0.028 |

Post-hoc: UA vs. FS

| Fixed effects | Estimate | Lower 95% CI | Upper 95% CI | t | p |
| --- | --- | --- | --- | --- | --- |
| RA:FS | 0.005 | -0.010 | 0.021 | 0.685 | 1.000 |
| ES:FS | -0.007 | -0.023 | 0.008 | -0.927 | 1.000 |
| Pec:FS | 0.006 | -0.009 | 0.021 | 0.747 | 1.000 |
| Lat:FS | 0.018 | 0.003 | 0.034 | 2.344 | 0.058 |
| Tra:FS | -0.010 | -0.026 | 0.005 | -1.338 | 0.543 |
| Del:FS | 0.007 | -0.009 | 0.022 | 0.854 | 1.000 |
| Bic:FS | 0.035 | 0.019 | 0.050 | 4.451 | <0.001 |
| Tri:FS | 0.042 | 0.027 | 0.058 | 5.408 | <0.001 |
| GM:FS | -0.016 | -0.031 | -0.000 | -2.000 | 0.137 |
| TF:FS | -0.012 | -0.027 | 0.004 | -1.494 | 0.406 |
| RF:FS | -0.004 | -0.019 | 0.012 | -0.456 | 1.000 |
| VM:FS | 0.003 | -0.012 | 0.018 | 0.367 | 1.000 |
| BF:FS | -0.006 | -0.021 | 0.009 | -0.754 | 1.000 |
| TA:FS | -0.018 | -0.033 | -0.002 | -2.257 | 0.072 |
| PL:FS | -0.029 | -0.045 | -0.014 | -3.751 | 0.001 |

| Random effects | Estimate | Lower 95% CI | Upper 95% CI |
| --- | --- | --- | --- |
| (Intercept) | 0.005 | 0.003 | 0.007 |
| Error | 0.027 | 0.027 | 0.028 |

Post-hoc: LT vs. FS

| Fixed effects | Estimate | Lower 95% CI | Upper 95% CI | t | p |
| --- | --- | --- | --- | --- | --- |
| RA:FS | 0.006 | -0.005 | 0.017 | 1.028 | 0.913 |
| ES:FS | -0.023 | -0.034 | -0.012 | -3.983 | <0.001 |
| Pec:FS | 0.003 | -0.008 | 0.014 | 0.540 | 1.000 |
| Lat:FS | 0.010 | -0.001 | 0.022 | 1.795 | 0.218 |
| Tra:FS | -0.006 | -0.017 | 0.006 | -0.987 | 0.972 |
| Del:FS | -0.020 | -0.031 | -0.009 | -3.480 | 0.002 |
| Bic:FS | 0.020 | 0.009 | 0.031 | 3.477 | 0.002 |
| Tri:FS | 0.016 | 0.004 | 0.027 | 2.737 | 0.019 |
| GM:FS | -0.003 | -0.014 | 0.008 | -0.499 | 1.000 |
| TF:FS | -0.000 | -0.012 | 0.011 | -0.069 | 1.000 |
| RF:FS | -0.012 | -0.023 | -0.001 | -2.050 | 0.122 |
| VM:FS | 0.003 | -0.008 | 0.015 | 0.587 | 1.000 |
| BF:FS | 0.000 | -0.011 | 0.011 | 0.037 | 1.000 |
| TA:FS | -0.002 | -0.013 | 0.010 | -0.298 | 1.000 |
| PL:FS | 0.005 | -0.006 | 0.016 | 0.832 | 1.000 |

| Random effects | Estimate | Lower 95% CI | Upper 95% CI |
| --- | --- | --- | --- |
| (Intercept) | 0.005 | 0.003 | 0.007 |
| Error | 0.027 | 0.027 | 0.028 |

**Detailed statistics: sit-to-stand, challenging condition, forward leaning strategy, synergy 5**

Reference group: UA

| Model information | Values |
| --- | --- |
| Number of observations | 1648 |
| Number of participants | 19 |
| ICC | 0.425 |

| Fixed effects | Estimate | Lower 95% CI | Upper 95% CI | t | p |
| --- | --- | --- | --- | --- | --- |
| (Intercept) | 0.041 | 0.035 | 0.046 | 14.535 | <0.001 |
| LT | 0.002 | -0.003 | 0.007 | 0.733 | 1.000 |
| FS | -0.011 | -0.016 | -0.006 | -4.326 | <0.001 |

| Random effects | Estimate | Lower 95% CI | Upper 95% CI |
| --- | --- | --- | --- |
| (Intercept) | 0.007 | 0.005 | 0.010 |
| Error | 0.033 | 0.032 | 0.034 |

Reference group: LT

| Fixed effects | Estimate | Lower 95% CI | Upper 95% CI | t | p |
| --- | --- | --- | --- | --- | --- |
| (Intercept) | 0.043 | 0.039 | 0.047 | 20.042 | <0.001 |
| UA | -0.002 | -0.007 | 0.003 | -0.733 | 1.000 |
| FS | -0.013 | -0.017 | -0.010 | -7.349 | <0.001 |

| Random effects | Estimate | Lower 95% CI | Upper 95% CI |
| --- | --- | --- | --- |
| (Intercept) | 0.007 | 0.005 | 0.010 |
| Error | 0.033 | 0.032 | 0.034 |

Post-hoc: UA vs. FS

| Fixed effects | Estimate | Lower 95% CI | Upper 95% CI | t | p |
| --- | --- | --- | --- | --- | --- |
| RA:FS | -0.004 | -0.019 | 0.011 | -0.499 | 1.000 |
| ES:FS | -0.023 | -0.038 | -0.008 | -2.974 | 0.006 |
| Pec:FS | 0.004 | -0.011 | 0.019 | 0.472 | 1.000 |
| Lat:FS | 0.018 | 0.003 | 0.033 | 2.374 | 0.035 |
| Tra:FS | 0.013 | -0.002 | 0.028 | 1.645 | 0.200 |
| Del:FS | 0.024 | 0.009 | 0.039 | 3.151 | 0.003 |
| Bic:FS | 0.032 | 0.017 | 0.047 | 4.159 | <0.001 |
| Tri:FS | 0.033 | 0.018 | 0.048 | 4.358 | <0.001 |
| GM:FS | -0.024 | -0.039 | -0.009 | -3.157 | 0.003 |
| TF:FS | -0.019 | -0.034 | -0.004 | -2.464 | 0.028 |
| RF:FS | -0.020 | -0.035 | -0.005 | -2.575 | 0.020 |
| VM:FS | -0.012 | -0.027 | 0.003 | -1.547 | 0.244 |
| BF:FS | -0.007 | -0.022 | 0.008 | -0.856 | 0.784 |
| TA:FS | 0.010 | -0.005 | 0.025 | 1.281 | 0.401 |
| PL:FS | -0.016 | -0.031 | -0.001 | -2.079 | 0.076 |

| Random effects | Estimate | Lower 95% CI | Upper 95% CI |
| --- | --- | --- | --- |
| (Intercept) | 0.007 | 0.005 | 0.011 |
| Error | 0.027 | 0.026 | 0.028 |

Post-hoc: LT vs. FS

| Fixed effects | Estimate | Lower 95% CI | Upper 95% CI | t | p |
| --- | --- | --- | --- | --- | --- |
| RA:FS | 0.011 | -0.000 | 0.022 | 1.950 | 0.103 |
| ES:FS | -0.011 | -0.022 | 0.000 | -1.902 | 0.115 |
| Pec:FS | 0.004 | -0.007 | 0.015 | 0.636 | 1.000 |
| Lat:FS | 0.012 | 0.001 | 0.023 | 2.080 | 0.075 |
| Tra:FS | 0.003 | -0.008 | 0.014 | 0.484 | 1.000 |
| Del:FS | -0.025 | -0.037 | -0.014 | -4.520 | <0.001 |
| Bic:FS | 0.029 | 0.018 | 0.040 | 5.165 | <0.001 |
| Tri:FS | 0.005 | -0.006 | 0.016 | 0.926 | 0.709 |
| GM:FS | 0.000 | -0.011 | 0.011 | 0.015 | 1.000 |
| TF:FS | -0.011 | -0.022 | 0.000 | -1.919 | 0.110 |
| RF:FS | -0.012 | -0.023 | -0.001 | -2.160 | 0.062 |
| VM:FS | -0.007 | -0.018 | 0.004 | -1.183 | 0.474 |
| BF:FS | 0.006 | -0.005 | 0.018 | 1.151 | 0.500 |
| TA:FS | -0.009 | -0.020 | 0.002 | -1.635 | 0.205 |
| PL:FS | -0.002 | -0.013 | 0.009 | -0.318 | 1.000 |

| Random effects | Estimate | Lower 95% CI | Upper 95% CI |
| --- | --- | --- | --- |
| (Intercept) | 0.007 | 0.005 | 0.011 |
| Error | 0.027 | 0.026 | 0.028 |

**Detailed statistics: sit-to-stand, challenging condition, forward leaning strategy, synergy 6**

Reference group: UA

| Model information | Values |
| --- | --- |
| Number of observations | 1648 |
| Number of participants | 19 |
| ICC | 0.491 |

| Fixed effects | Estimate | Lower 95% CI | Upper 95% CI | t | p |
| --- | --- | --- | --- | --- | --- |
| (Intercept) | 0.047 | 0.041 | 0.053 | 15.373 | <0.001 |
| LT | -0.006 | -0.012 | -0.001 | -2.275 | 0.369 |
| FS | -0.021 | -0.026 | -0.016 | -7.794 | <0.001 |

| Random effects | Estimate | Lower 95% CI | Upper 95% CI |
| --- | --- | --- | --- |
| (Intercept) | 0.008 | 0.006 | 0.012 |
| Error | 0.034 | 0.033 | 0.035 |

Reference group: LT

| Fixed effects | Estimate | Lower 95% CI | Upper 95% CI | t | p |
| --- | --- | --- | --- | --- | --- |
| (Intercept) | 0.040 | 0.036 | 0.045 | 16.853 | <0.001 |
| UA | 0.006 | 0.001 | 0.012 | 2.275 | 0.369 |
| FS | -0.015 | -0.018 | -0.011 | -7.813 | <0.001 |

| Random effects | Estimate | Lower 95% CI | Upper 95% CI |
| --- | --- | --- | --- |
| (Intercept) | 0.008 | 0.006 | 0.012 |
| Error | 0.034 | 0.033 | 0.035 |

Post-hoc: UA vs. FS

| Fixed effects | Estimate | Lower 95% CI | Upper 95% CI | t | p |
| --- | --- | --- | --- | --- | --- |
| RA:FS | -0.000 | -0.017 | 0.016 | -0.045 | 1.000 |
| ES:FS | -0.013 | -0.029 | 0.004 | -1.523 | 0.256 |
| Pec:FS | 0.006 | -0.011 | 0.022 | 0.683 | 0.990 |
| Lat:FS | 0.034 | 0.017 | 0.050 | 4.040 | <0.001 |
| Tra:FS | 0.009 | -0.007 | 0.026 | 1.130 | 0.517 |
| Del:FS | 0.028 | 0.012 | 0.045 | 3.404 | 0.001 |
| Bic:FS | 0.020 | 0.004 | 0.037 | 2.462 | 0.028 |
| Tri:FS | 0.051 | 0.034 | 0.067 | 6.105 | <0.001 |
| GM:FS | -0.002 | -0.018 | 0.014 | -0.254 | 1.000 |
| TF:FS | 0.007 | -0.009 | 0.023 | 0.835 | 0.807 |
| RF:FS | 0.001 | -0.015 | 0.017 | 0.112 | 1.000 |
| VM:FS | 0.022 | 0.005 | 0.038 | 2.612 | 0.018 |
| BF:FS | -0.023 | -0.039 | -0.006 | -2.735 | 0.013 |
| TA:FS | -0.036 | -0.053 | -0.020 | -4.351 | <0.001 |
| PL:FS | -0.062 | -0.079 | -0.046 | -7.474 | <0.001 |

| Random effects | Estimate | Lower 95% CI | Upper 95% CI |
| --- | --- | --- | --- |
| (Intercept) | 0.008 | 0.006 | 0.012 |
| Error | 0.029 | 0.028 | 0.030 |

Post-hoc: LT vs. FS

| Fixed effects | Estimate | Lower 95% CI | Upper 95% CI | t | p |
| --- | --- | --- | --- | --- | --- |
| RA:FS | 0.008 | -0.004 | 0.020 | 1.378 | 0.337 |
| ES:FS | 0.003 | -0.009 | 0.015 | 0.548 | 1.000 |
| Pec:FS | 0.009 | -0.003 | 0.021 | 1.479 | 0.279 |
| Lat:FS | 0.002 | -0.010 | 0.014 | 0.247 | 1.000 |
| Tra:FS | 0.010 | -0.002 | 0.022 | 1.653 | 0.197 |
| Del:FS | -0.012 | -0.024 | -0.000 | -1.970 | 0.098 |
| Bic:FS | 0.014 | 0.002 | 0.026 | 2.348 | 0.038 |
| Tri:FS | -0.003 | -0.015 | 0.009 | -0.462 | 1.000 |
| GM:FS | 0.005 | -0.007 | 0.017 | 0.829 | 0.814 |
| TF:FS | -0.009 | -0.021 | 0.003 | -1.418 | 0.312 |
| RF:FS | 0.001 | -0.011 | 0.013 | 0.221 | 1.000 |
| VM:FS | -0.002 | -0.014 | 0.010 | -0.254 | 1.000 |
| BF:FS | -0.005 | -0.017 | 0.007 | -0.808 | 0.839 |
| TA:FS | -0.021 | -0.033 | -0.009 | -3.399 | 0.001 |
| PL:FS | -0.006 | -0.018 | 0.006 | -1.040 | 0.597 |

| Random effects | Estimate | Lower 95% CI | Upper 95% CI |
| --- | --- | --- | --- |
| (Intercept) | 0.008 | 0.006 | 0.012 |
| Error | 0.029 | 0.028 | 0.030 |

**Detailed statistics: sit-to-stand, challenging condition, forward leaning strategy, synergy 9**

Reference group: UA

| Model information | Values |
| --- | --- |
| Number of observations | 1648 |
| Number of participants | 19 |
| ICC | 0.392 |

| Fixed effects | Estimate | Lower 95% CI | Upper 95% CI | t | p |
| --- | --- | --- | --- | --- | --- |
| (Intercept) | 0.063 | 0.057 | 0.069 | 20.377 | <0.001 |
| LT | -0.025 | -0.031 | -0.019 | -8.061 | <0.001 |
| FS | -0.031 | -0.036 | -0.025 | -10.380 | <0.001 |

| Random effects | Estimate | Lower 95% CI | Upper 95% CI |
| --- | --- | --- | --- |
| (Intercept) | 0.007 | 0.005 | 0.011 |
| Error | 0.037 | 0.036 | 0.038 |

Reference group: LT

| Fixed effects | Estimate | Lower 95% CI | Upper 95% CI | t | p |
| --- | --- | --- | --- | --- | --- |
| (Intercept) | 0.038 | 0.033 | 0.043 | 16.412 | <0.001 |
| UA | 0.025 | 0.019 | 0.031 | 8.061 | <0.001 |
| FS | -0.006 | -0.010 | -0.002 | -2.722 | 0.105 |

| Random effects | Estimate | Lower 95% CI | Upper 95% CI |
| --- | --- | --- | --- |
| (Intercept) | 0.007 | 0.005 | 0.011 |
| Error | 0.037 | 0.036 | 0.038 |

Post-hoc: UA vs. LT

| Fixed effects | Estimate | Lower 95% CI | Upper 95% CI | t | p |
| --- | --- | --- | --- | --- | --- |
| RA:LT | -0.007 | -0.025 | 0.011 | -0.753 | 0.903 |
| ES:LT | -0.025 | -0.043 | -0.007 | -2.764 | 0.012 |
| Pec:LT | 0.019 | 0.002 | 0.037 | 2.128 | 0.067 |
| Lat:LT | 0.044 | 0.026 | 0.062 | 4.838 | <0.001 |
| Tra:LT | 0.014 | -0.003 | 0.032 | 1.580 | 0.229 |
| Del:LT | 0.037 | 0.019 | 0.055 | 4.076 | <0.001 |
| Bic:LT | 0.015 | -0.002 | 0.033 | 1.698 | 0.179 |
| Tri:LT | 0.059 | 0.041 | 0.077 | 6.507 | <0.001 |
| GM:LT | -0.018 | -0.036 | -0.001 | -2.028 | 0.085 |
| TF:LT | 0.007 | -0.011 | 0.025 | 0.797 | 0.851 |
| RF:LT | 0.010 | -0.008 | 0.028 | 1.127 | 0.520 |
| VM:LT | 0.013 | -0.005 | 0.030 | 1.388 | 0.331 |
| BF:LT | -0.052 | -0.070 | -0.034 | -5.713 | <0.001 |
| TA:LT | -0.056 | -0.074 | -0.038 | -6.186 | <0.001 |
| PL:LT | -0.043 | -0.061 | -0.025 | -4.731 | <0.001 |

| Random effects | Estimate | Lower 95% CI | Upper 95% CI |
| --- | --- | --- | --- |
| (Intercept) | 0.008 | 0.005 | 0.011 |
| Error | 0.031 | 0.030 | 0.032 |

Post-hoc: UA vs. FS

| Fixed effects | Estimate | Lower 95% CI | Upper 95% CI | t | p |
| --- | --- | --- | --- | --- | --- |
| RA:FS | -0.006 | -0.024 | 0.011 | -0.731 | 0.930 |
| ES:FS | -0.035 | -0.052 | -0.018 | -3.990 | <0.001 |
| Pec:FS | 0.033 | 0.016 | 0.051 | 3.826 | <0.001 |
| Lat:FS | 0.035 | 0.018 | 0.052 | 4.021 | <0.001 |
| Tra:FS | 0.022 | 0.005 | 0.040 | 2.572 | 0.020 |
| Del:FS | 0.020 | 0.003 | 0.038 | 2.336 | 0.039 |
| Bic:FS | 0.020 | 0.002 | 0.037 | 2.233 | 0.051 |
| Tri:FS | 0.072 | 0.054 | 0.089 | 8.197 | <0.001 |
| GM:FS | -0.029 | -0.046 | -0.011 | -3.274 | 0.002 |
| TF:FS | 0.008 | -0.009 | 0.025 | 0.899 | 0.738 |
| RF:FS | 0.013 | -0.004 | 0.031 | 1.541 | 0.247 |
| VM:FS | 0.014 | -0.003 | 0.031 | 1.624 | 0.209 |
| BF:FS | -0.049 | -0.066 | -0.032 | -5.642 | <0.001 |
| TA:FS | -0.066 | -0.083 | -0.049 | -7.568 | <0.001 |
| PL:FS | -0.042 | -0.059 | -0.025 | -4.795 | <0.001 |

| Random effects | Estimate | Lower 95% CI | Upper 95% CI |
| --- | --- | --- | --- |
| (Intercept) | 0.008 | 0.005 | 0.011 |
| Error | 0.031 | 0.030 | 0.032 |

**Detailed statistics: sit-to-stand, challenging condition, forward leaning strategy, synergy 11**

Reference group: UA

| Model information | Values |
| --- | --- |
| Number of observations | 1648 |
| Number of participants | 19 |
| ICC | 0.308 |

| Fixed effects | Estimate | Lower 95% CI | Upper 95% CI | t | p |
| --- | --- | --- | --- | --- | --- |
| (Intercept) | 0.069 | 0.062 | 0.076 | 18.872 | <0.001 |
| LT | -0.026 | -0.034 | -0.019 | -6.779 | <0.001 |
| FS | -0.028 | -0.035 | -0.021 | -7.591 | <0.001 |

| Random effects | Estimate | Lower 95% CI | Upper 95% CI |
| --- | --- | --- | --- |
| (Intercept) | 0.007 | 0.004 | 0.012 |
| Error | 0.047 | 0.045 | 0.048 |

Reference group: LT

| Fixed effects | Estimate | Lower 95% CI | Upper 95% CI | t | p |
| --- | --- | --- | --- | --- | --- |
| (Intercept) | 0.042 | 0.037 | 0.048 | 16.357 | <0.001 |
| UA | 0.026 | 0.019 | 0.034 | 6.779 | <0.001 |
| FS | -0.002 | -0.007 | 0.003 | -0.653 | 1.000 |

| Random effects | Estimate | Lower 95% CI | Upper 95% CI |
| --- | --- | --- | --- |
| (Intercept) | 0.007 | 0.004 | 0.012 |
| Error | 0.047 | 0.045 | 0.048 |

Post-hoc: UA vs. LT

| Fixed effects | Estimate | Lower 95% CI | Upper 95% CI | t | p |
| --- | --- | --- | --- | --- | --- |
| RA:LT | 0.003 | -0.017 | 0.023 | 0.264 | 1.000 |
| ES:LT | -0.013 | -0.033 | 0.007 | -1.238 | 0.432 |
| Pec:LT | 0.026 | 0.006 | 0.046 | 2.548 | 0.022 |
| Lat:LT | 0.028 | 0.008 | 0.048 | 2.718 | 0.013 |
| Tra:LT | 0.019 | -0.001 | 0.039 | 1.838 | 0.132 |
| Del:LT | 0.015 | -0.005 | 0.035 | 1.442 | 0.299 |
| Bic:LT | 0.007 | -0.013 | 0.027 | 0.712 | 0.953 |
| Tri:LT | 0.043 | 0.023 | 0.063 | 4.220 | <0.001 |
| GM:LT | -0.012 | -0.032 | 0.008 | -1.191 | 0.467 |
| TF:LT | 0.028 | 0.008 | 0.048 | 2.735 | 0.013 |
| RF:LT | 0.028 | 0.008 | 0.048 | 2.727 | 0.013 |
| VM:LT | 0.025 | 0.005 | 0.045 | 2.483 | 0.026 |
| BF:LT | 0.020 | 0.000 | 0.040 | 1.970 | 0.098 |
| TA:LT | -0.075 | -0.095 | -0.055 | -7.376 | <0.001 |
| PL:LT | -0.080 | -0.100 | -0.060 | -7.834 | <0.001 |

| Random effects | Estimate | Lower 95% CI | Upper 95% CI |
| --- | --- | --- | --- |
| (Intercept) | 0.009 | 0.006 | 0.013 |
| Error | 0.034 | 0.033 | 0.036 |

Post-hoc: UA vs. FS

| Fixed effects | Estimate | Lower 95% CI | Upper 95% CI | t | p |
| --- | --- | --- | --- | --- | --- |
| RA:FS | 0.008 | -0.011 | 0.027 | 0.791 | 0.858 |
| ES:FS | -0.011 | -0.030 | 0.008 | -1.093 | 0.549 |
| Pec:FS | 0.035 | 0.016 | 0.054 | 3.554 | 0.001 |
| Lat:FS | 0.029 | 0.010 | 0.048 | 2.943 | 0.007 |
| Tra:FS | 0.023 | 0.004 | 0.042 | 2.353 | 0.038 |
| Del:FS | 0.017 | -0.002 | 0.037 | 1.784 | 0.149 |
| Bic:FS | 0.017 | -0.003 | 0.036 | 1.688 | 0.183 |
| Tri:FS | 0.085 | 0.066 | 0.104 | 8.687 | <0.001 |
| GM:FS | -0.026 | -0.046 | -0.007 | -2.707 | 0.014 |
| TF:FS | 0.021 | 0.001 | 0.040 | 2.102 | 0.071 |
| RF:FS | 0.014 | -0.005 | 0.033 | 1.418 | 0.313 |
| VM:FS | 0.017 | -0.002 | 0.036 | 1.709 | 0.175 |
| BF:FS | -0.013 | -0.032 | 0.006 | -1.317 | 0.376 |
| TA:FS | -0.073 | -0.092 | -0.053 | -7.434 | <0.001 |
| PL:FS | -0.081 | -0.100 | -0.061 | -8.240 | <0.001 |

| Random effects | Estimate | Lower 95% CI | Upper 95% CI |
| --- | --- | --- | --- |
| (Intercept) | 0.009 | 0.006 | 0.013 |
| Error | 0.034 | 0.033 | 0.036 |

## Stand-to-sit, non-challenging condition

**Detailed statistics: stand-to-sit, non-challenging condition, backward lowering strategy, synergy 1**

Reference group: UA

| Model information | Values |
| --- | --- |
| Number of observations | 672 |
| Number of participants | 14 |
| ICC | 0.542 |

| Fixed effects | Estimate | Lower 95% CI | Upper 95% CI | t | p |
| --- | --- | --- | --- | --- | --- |
| (Intercept) | 0.024 | 0.018 | 0.030 | 8.040 | <0.001 |
| LT | -0.004 | -0.011 | 0.002 | -1.421 | 1.000 |
| FS | -0.005 | -0.011 | 0.000 | -1.903 | 0.919 |

| Random effects | Estimate | Lower 95% CI | Upper 95% CI |
| --- | --- | --- | --- |
| (Intercept) | 0.005 | 0.003 | 0.009 |
| Error | 0.020 | 0.019 | 0.021 |

Reference group: LT

| Fixed effects | Estimate | Lower 95% CI | Upper 95% CI | t | p |
| --- | --- | --- | --- | --- | --- |
| (Intercept) | 0.020 | 0.015 | 0.024 | 8.254 | <0.001 |
| UA | 0.004 | -0.002 | 0.011 | 1.421 | 1.000 |
| FS | -0.001 | -0.005 | 0.003 | -0.405 | 1.000 |

| Random effects | Estimate | Lower 95% CI | Upper 95% CI |
| --- | --- | --- | --- |
| (Intercept) | 0.005 | 0.003 | 0.009 |
| Error | 0.020 | 0.019 | 0.021 |

**Detailed statistics: stand-to-sit, non-challenging condition, backward lowering strategy, synergy 2**

Reference group: UA

| Model information | Values |
| --- | --- |
| Number of observations | 672 |
| Number of participants | 14 |
| ICC | 0.632 |

| Fixed effects | Estimate | Lower 95% CI | Upper 95% CI | t | p |
| --- | --- | --- | --- | --- | --- |
| (Intercept) | 0.027 | 0.020 | 0.034 | 7.185 | <0.001 |
| LT | 0.005 | -0.002 | 0.013 | 1.382 | 1.000 |
| FS | 0.002 | -0.004 | 0.009 | 0.687 | 1.000 |

| Random effects | Estimate | Lower 95% CI | Upper 95% CI |
| --- | --- | --- | --- |
| (Intercept) | 0.008 | 0.005 | 0.012 |
| Error | 0.024 | 0.023 | 0.025 |

Reference group: LT

| Fixed effects | Estimate | Lower 95% CI | Upper 95% CI | t | p |
| --- | --- | --- | --- | --- | --- |
| (Intercept) | 0.032 | 0.026 | 0.038 | 10.525 | <0.001 |
| UA | -0.005 | -0.013 | 0.002 | -1.382 | 1.000 |
| FS | -0.003 | -0.008 | 0.002 | -1.100 | 1.000 |

| Random effects | Estimate | Lower 95% CI | Upper 95% CI |
| --- | --- | --- | --- |
| (Intercept) | 0.008 | 0.005 | 0.012 |
| Error | 0.024 | 0.023 | 0.025 |

**Detailed statistics: stand-to-sit, non-challenging condition, backward lowering strategy, synergy 3**

Reference group: UA

| Model information | Values |
| --- | --- |
| Number of observations | 672 |
| Number of participants | 14 |
| ICC | 0.708 |

| Fixed effects | Estimate | Lower 95% CI | Upper 95% CI | t | p |
| --- | --- | --- | --- | --- | --- |
| (Intercept) | 0.024 | 0.016 | 0.032 | 6.075 | <0.001 |
| LT | 0.007 | -0.000 | 0.015 | 1.903 | 0.920 |
| FS | 0.002 | -0.004 | 0.009 | 0.692 | 1.000 |

| Random effects | Estimate | Lower 95% CI | Upper 95% CI |
| --- | --- | --- | --- |
| (Intercept) | 0.009 | 0.005 | 0.014 |
| Error | 0.024 | 0.023 | 0.025 |

Reference group: LT

| Fixed effects | Estimate | Lower 95% CI | Upper 95% CI | t | p |
| --- | --- | --- | --- | --- | --- |
| (Intercept) | 0.031 | 0.025 | 0.038 | 9.557 | <0.001 |
| UA | -0.007 | -0.015 | 0.000 | -1.903 | 0.920 |
| FS | -0.005 | -0.010 | 0.000 | -1.835 | 1.000 |

| Random effects | Estimate | Lower 95% CI | Upper 95% CI |
| --- | --- | --- | --- |
| (Intercept) | 0.009 | 0.005 | 0.014 |
| Error | 0.024 | 0.023 | 0.025 |

**Detailed statistics: stand-to-sit, non-challenging condition, backward lowering strategy, synergy 5**

Reference group: UA

| Model information | Values |
| --- | --- |
| Number of observations | 672 |
| Number of participants | 14 |
| ICC | 0.593 |

| Fixed effects | Estimate | Lower 95% CI | Upper 95% CI | t | p |
| --- | --- | --- | --- | --- | --- |
| (Intercept) | 0.025 | 0.019 | 0.032 | 7.354 | <0.001 |
| LT | -0.002 | -0.009 | 0.005 | -0.619 | 1.000 |
| FS | -0.005 | -0.011 | 0.001 | -1.502 | 1.000 |

| Random effects | Estimate | Lower 95% CI | Upper 95% CI |
| --- | --- | --- | --- |
| (Intercept) | 0.007 | 0.004 | 0.011 |
| Error | 0.022 | 0.021 | 0.024 |

Reference group: LT

| Fixed effects | Estimate | Lower 95% CI | Upper 95% CI | t | p |
| --- | --- | --- | --- | --- | --- |
| (Intercept) | 0.023 | 0.018 | 0.029 | 8.349 | <0.001 |
| UA | 0.002 | -0.005 | 0.009 | 0.619 | 1.000 |
| FS | -0.003 | -0.007 | 0.002 | -1.040 | 1.000 |

| Random effects | Estimate | Lower 95% CI | Upper 95% CI |
| --- | --- | --- | --- |
| (Intercept) | 0.007 | 0.004 | 0.011 |
| Error | 0.022 | 0.021 | 0.024 |

**Detailed statistics: stand-to-sit, non-challenging condition, backward lowering strategy, synergy 6**

Reference group: UA

| Model information | Values |
| --- | --- |
| Number of observations | 672 |
| Number of participants | 14 |
| ICC | 0.484 |

| Fixed effects | Estimate | Lower 95% CI | Upper 95% CI | t | p |
| --- | --- | --- | --- | --- | --- |
| (Intercept) | 0.025 | 0.017 | 0.033 | 6.271 | <0.001 |
| LT | -0.003 | -0.011 | 0.005 | -0.759 | 1.000 |
| FS | 0.000 | -0.007 | 0.008 | 0.052 | 1.000 |

| Random effects | Estimate | Lower 95% CI | Upper 95% CI |
| --- | --- | --- | --- |
| (Intercept) | 0.007 | 0.004 | 0.011 |
| Error | 0.027 | 0.026 | 0.029 |

Reference group: LT

| Fixed effects | Estimate | Lower 95% CI | Upper 95% CI | t | p |
| --- | --- | --- | --- | --- | --- |
| (Intercept) | 0.022 | 0.016 | 0.028 | 6.965 | <0.001 |
| UA | 0.003 | -0.005 | 0.011 | 0.759 | 1.000 |
| FS | 0.003 | -0.002 | 0.009 | 1.161 | 1.000 |

| Random effects | Estimate | Lower 95% CI | Upper 95% CI |
| --- | --- | --- | --- |
| (Intercept) | 0.007 | 0.004 | 0.011 |
| Error | 0.027 | 0.026 | 0.029 |

**Detailed statistics: stand-to-sit, non-challenging condition, backward lowering strategy, synergy 8**

Reference group: UA

| Model information | Values |
| --- | --- |
| Number of observations | 672 |
| Number of participants | 14 |
| ICC | 0.817 |

| Fixed effects | Estimate | Lower 95% CI | Upper 95% CI | t | p |
| --- | --- | --- | --- | --- | --- |
| (Intercept) | 0.027 | 0.019 | 0.036 | 6.465 | <0.001 |
| LT | -0.005 | -0.012 | 0.003 | -1.176 | 1.000 |
| FS | -0.006 | -0.013 | 0.001 | -1.688 | 1.000 |

| Random effects | Estimate | Lower 95% CI | Upper 95% CI |
| --- | --- | --- | --- |
| (Intercept) | 0.010 | 0.006 | 0.016 |
| Error | 0.024 | 0.023 | 0.026 |

Reference group: LT

| Fixed effects | Estimate | Lower 95% CI | Upper 95% CI | t | p |
| --- | --- | --- | --- | --- | --- |
| (Intercept) | 0.023 | 0.016 | 0.030 | 6.376 | <0.001 |
| UA | 0.005 | -0.003 | 0.012 | 1.176 | 1.000 |
| FS | -0.001 | -0.007 | 0.004 | -0.476 | 1.000 |

| Random effects | Estimate | Lower 95% CI | Upper 95% CI |
| --- | --- | --- | --- |
| (Intercept) | 0.010 | 0.006 | 0.016 |
| Error | 0.024 | 0.023 | 0.026 |

**Detailed statistics: stand-to-sit, non-challenging condition, backward lowering strategy, synergy 9**

Reference group: UA

| Model information | Values |
| --- | --- |
| Number of observations | 672 |
| Number of participants | 14 |
| ICC | 0.711 |

| Fixed effects | Estimate | Lower 95% CI | Upper 95% CI | t | p |
| --- | --- | --- | --- | --- | --- |
| (Intercept) | 0.015 | 0.008 | 0.022 | 4.170 | 0.001 |
| LT | 0.007 | 0.000 | 0.014 | 2.036 | 0.675 |
| FS | 0.006 | -0.000 | 0.012 | 1.956 | 0.814 |

| Random effects | Estimate | Lower 95% CI | Upper 95% CI |
| --- | --- | --- | --- |
| (Intercept) | 0.008 | 0.005 | 0.012 |
| Error | 0.022 | 0.021 | 0.023 |

Reference group: LT

| Fixed effects | Estimate | Lower 95% CI | Upper 95% CI | t | p |
| --- | --- | --- | --- | --- | --- |
| (Intercept) | 0.022 | 0.016 | 0.028 | 7.400 | <0.001 |
| UA | -0.007 | -0.014 | -0.000 | -2.036 | 0.675 |
| FS | -0.001 | -0.006 | 0.004 | -0.409 | 1.000 |

| Random effects | Estimate | Lower 95% CI | Upper 95% CI |
| --- | --- | --- | --- |
| (Intercept) | 0.008 | 0.005 | 0.012 |
| Error | 0.022 | 0.021 | 0.023 |

**Detailed statistics: stand-to-sit, non-challenging condition, backward lowering strategy, synergy 10**

Reference group: UA

| Model information | Values |
| --- | --- |
| Number of observations | 672 |
| Number of participants | 14 |
| ICC | 0.373 |

| Fixed effects | Estimate | Lower 95% CI | Upper 95% CI | t | p |
| --- | --- | --- | --- | --- | --- |
| (Intercept) | 0.018 | 0.010 | 0.025 | 4.679 | <0.001 |
| LT | 0.002 | -0.006 | 0.010 | 0.430 | 1.000 |
| FS | 0.003 | -0.004 | 0.010 | 0.849 | 1.000 |

| Random effects | Estimate | Lower 95% CI | Upper 95% CI |
| --- | --- | --- | --- |
| (Intercept) | 0.005 | 0.003 | 0.009 |
| Error | 0.027 | 0.026 | 0.029 |

Reference group: LT

| Fixed effects | Estimate | Lower 95% CI | Upper 95% CI | t | p |
| --- | --- | --- | --- | --- | --- |
| (Intercept) | 0.019 | 0.014 | 0.025 | 6.745 | <0.001 |
| UA | -0.002 | -0.010 | 0.006 | -0.430 | 1.000 |
| FS | 0.001 | -0.004 | 0.007 | 0.476 | 1.000 |

| Random effects | Estimate | Lower 95% CI | Upper 95% CI |
| --- | --- | --- | --- |
| (Intercept) | 0.005 | 0.003 | 0.009 |
| Error | 0.027 | 0.026 | 0.029 |

**Detailed statistics: stand-to-sit, non-challenging condition, hybrid strategy, synergy 1**

Reference group: UA

| Model information | Values |
| --- | --- |
| Number of observations | 1152 |
| Number of participants | 14 |
| ICC | 0.723 |

| Fixed effects | Estimate | Lower 95% CI | Upper 95% CI | t | p |
| --- | --- | --- | --- | --- | --- |
| (Intercept) | 0.021 | 0.016 | 0.026 | 8.813 | <0.001 |
| LT | 0.002 | -0.002 | 0.005 | 0.995 | 1.000 |
| FS | 0.003 | -0.000 | 0.006 | 1.775 | 1.000 |

| Random effects | Estimate | Lower 95% CI | Upper 95% CI |
| --- | --- | --- | --- |
| (Intercept) | 0.007 | 0.005 | 0.011 |
| Error | 0.020 | 0.019 | 0.021 |

Reference group: LT

| Fixed effects | Estimate | Lower 95% CI | Upper 95% CI | t | p |
| --- | --- | --- | --- | --- | --- |
| (Intercept) | 0.023 | 0.018 | 0.027 | 10.327 | <0.001 |
| UA | -0.002 | -0.005 | 0.002 | -0.995 | 1.000 |
| FS | 0.001 | -0.002 | 0.004 | 0.948 | 1.000 |

| Random effects | Estimate | Lower 95% CI | Upper 95% CI |
| --- | --- | --- | --- |
| (Intercept) | 0.007 | 0.005 | 0.011 |
| Error | 0.020 | 0.019 | 0.021 |

**Detailed statistics: stand-to-sit, non-challenging condition, hybrid strategy, synergy 2**

Reference group: UA

| Model information | Values |
| --- | --- |
| Number of observations | 1152 |
| Number of participants | 14 |
| ICC | 0.367 |

| Fixed effects | Estimate | Lower 95% CI | Upper 95% CI | t | p |
| --- | --- | --- | --- | --- | --- |
| (Intercept) | 0.031 | 0.027 | 0.035 | 15.256 | <0.001 |
| LT | -0.002 | -0.006 | 0.001 | -1.259 | 1.000 |
| FS | -0.004 | -0.008 | 0.000 | -1.887 | 0.950 |

| Random effects | Estimate | Lower 95% CI | Upper 95% CI |
| --- | --- | --- | --- |
| (Intercept) | 0.005 | 0.003 | 0.008 |
| Error | 0.025 | 0.024 | 0.026 |

Reference group: LT

| Fixed effects | Estimate | Lower 95% CI | Upper 95% CI | t | p |
| --- | --- | --- | --- | --- | --- |
| (Intercept) | 0.029 | 0.025 | 0.032 | 16.716 | <0.001 |
| UA | 0.002 | -0.001 | 0.006 | 1.259 | 1.000 |
| FS | -0.001 | -0.005 | 0.002 | -0.798 | 1.000 |

| Random effects | Estimate | Lower 95% CI | Upper 95% CI |
| --- | --- | --- | --- |
| (Intercept) | 0.005 | 0.003 | 0.008 |
| Error | 0.025 | 0.024 | 0.026 |

**Detailed statistics: stand-to-sit, non-challenging condition, hybrid strategy, synergy 3**

Reference group: UA

| Model information | Values |
| --- | --- |
| Number of observations | 1152 |
| Number of participants | 14 |
| ICC | 0.090 |

| Fixed effects | Estimate | Lower 95% CI | Upper 95% CI | t | p |
| --- | --- | --- | --- | --- | --- |
| (Intercept) | 0.030 | 0.027 | 0.033 | 18.009 | <.001 |
| LT | -0.002 | -0.006 | 0.002 | -1.153 | 1.000 |
| FS | -0.003 | -0.007 | 0.001 | -1.445 | 1.000 |

| Random effects | Estimate | Lower 95% CI | Upper 95% CI |
| --- | --- | --- | --- |
| (Intercept) | 0.001 | 0.000 | 0.029 |
| Error | 0.028 | 0.026 | 0.029 |

Reference group: LT

| Fixed effects | Estimate | Lower 95% CI | Upper 95% CI | t | p |
| --- | --- | --- | --- | --- | --- |
| (Intercept) | 0.027 | 0.025 | 0.030 | 20.889 | <0.001 |
| UA | 0.002 | -0.002 | 0.006 | 1.153 | 1.000 |
| FS | -0.001 | -0.005 | 0.003 | -0.399 | 1.000 |

| Random effects | Estimate | Lower 95% CI | Upper 95% CI |
| --- | --- | --- | --- |
| (Intercept) | 0.001 | 0.000 | 0.029 |
| Error | 0.028 | 0.026 | 0.029 |

**Detailed statistics: stand-to-sit, non-challenging condition, hybrid strategy, synergy 5**

Reference group: UA

| Model information | Values |
| --- | --- |
| Number of observations | 1152 |
| Number of participants | 14 |
| ICC | 0.220 |

| Fixed effects | Estimate | Lower 95% CI | Upper 95% CI | t | p |
| --- | --- | --- | --- | --- | --- |
| (Intercept) | 0.027 | 0.023 | 0.030 | 15.080 | <0.001 |
| LT | -0.004 | -0.007 | 0.000 | -1.799 | 1.000 |
| FS | 0.002 | -0.002 | 0.006 | 0.861 | 1.000 |

| Random effects | Estimate | Lower 95% CI | Upper 95% CI |
| --- | --- | --- | --- |
| (Intercept) | 0.003 | 0.001 | 0.006 |
| Error | 0.026 | 0.025 | 0.027 |

Reference group: LT

| Fixed effects | Estimate | Lower 95% CI | Upper 95% CI | t | p |
| --- | --- | --- | --- | --- | --- |
| (Intercept) | 0.023 | 0.021 | 0.026 | 16.262 | <0.001 |
| UA | 0.004 | -0.000 | 0.007 | 1.799 | 1.000 |
| FS | 0.005 | 0.002 | 0.009 | 2.877 | 0.065 |

| Random effects | Estimate | Lower 95% CI | Upper 95% CI |
| --- | --- | --- | --- |
| (Intercept) | 0.003 | 0.001 | 0.006 |
| Error | 0.026 | 0.025 | 0.027 |

**Detailed statistics: stand-to-sit, non-challenging condition, hybrid strategy, synergy 6**

Reference group: UA

| Model information | Values |
| --- | --- |
| Number of observations | 1152 |
| Number of participants | 14 |
| ICC | 0.233 |

| Fixed effects | Estimate | Lower 95% CI | Upper 95% CI | t | p |
| --- | --- | --- | --- | --- | --- |
| (Intercept) | 0.032 | 0.028 | 0.036 | 15.827 | <0.001 |
| LT | -0.005 | -0.010 | -0.001 | -2.415 | 0.254 |
| FS | -0.006 | -0.011 | -0.002 | -2.640 | 0.134 |

| Random effects | Estimate | Lower 95% CI | Upper 95% CI |
| --- | --- | --- | --- |
| (Intercept) | 0.003 | 0.002 | 0.008 |
| Error | 0.029 | 0.028 | 0.031 |

Reference group: LT

| Fixed effects | Estimate | Lower 95% CI | Upper 95% CI | t | p |
| --- | --- | --- | --- | --- | --- |
| (Intercept) | 0.027 | 0.024 | 0.030 | 16.341 | <0.001 |
| UA | 0.005 | 0.001 | 0.010 | 2.415 | 0.254 |
| FS | -0.001 | -0.005 | 0.003 | -0.418 | 1.000 |

| Random effects | Estimate | Lower 95% CI | Upper 95% CI |
| --- | --- | --- | --- |
| (Intercept) | 0.003 | 0.002 | 0.008 |
| Error | 0.029 | 0.028 | 0.031 |

**Detailed statistics: stand-to-sit, non-challenging condition, hybrid strategy, synergy 7**

Reference group: UA

| Model information | Values |
| --- | --- |
| Number of observations | 1152 |
| Number of participants | 14 |
| ICC | 0.451 |

| Fixed effects | Estimate | Lower 95% CI | Upper 95% CI | t | p |
| --- | --- | --- | --- | --- | --- |
| (Intercept) | 0.022 | 0.017 | 0.026 | 9.963 | <0.001 |
| LT | 0.004 | 0.001 | 0.008 | 2.300 | 0.346 |
| FS | 0.003 | -0.001 | 0.007 | 1.380 | 1.000 |

| Random effects | Estimate | Lower 95% CI | Upper 95% CI |
| --- | --- | --- | --- |
| (Intercept) | 0.005 | 0.003 | 0.009 |
| Error | 0.024 | 0.023 | 0.025 |

Reference group: LT

| Fixed effects | Estimate | Lower 95% CI | Upper 95% CI | t | p |
| --- | --- | --- | --- | --- | --- |
| (Intercept) | 0.026 | 0.022 | 0.030 | 13.827 | <0.001 |
| UA | -0.004 | -0.008 | -0.001 | -2.300 | 0.346 |
| FS | -0.002 | -0.005 | 0.002 | -0.873 | 1.000 |

| Random effects | Estimate | Lower 95% CI | Upper 95% CI |
| --- | --- | --- | --- |
| (Intercept) | 0.005 | 0.003 | 0.009 |
| Error | 0.024 | 0.023 | 0.025 |

**Detailed statistics: stand-to-sit, non-challenging condition, hybrid strategy, synergy 8**

Reference group: UA

| Model information | Values |
| --- | --- |
| Number of observations | 1152 |
| Number of participants | 14 |
| ICC | 0.700 |

| Fixed effects | Estimate | Lower 95% CI | Upper 95% CI | t | p |
| --- | --- | --- | --- | --- | --- |
| (Intercept) | 0.022 | 0.016 | 0.029 | 6.893 | <0.001 |
| LT | 0.007 | 0.003 | 0.012 | 3.381 | 0.012 |
| FS | 0.002 | -0.002 | 0.007 | 0.899 | 1.000 |

| Random effects | Estimate | Lower 95% CI | Upper 95% CI |
| --- | --- | --- | --- |
| (Intercept) | 0.010 | 0.006 | 0.015 |
| Error | 0.028 | 0.027 | 0.029 |

Reference group: LT

| Fixed effects | Estimate | Lower 95% CI | Upper 95% CI | t | p |
| --- | --- | --- | --- | --- | --- |
| (Intercept) | 0.030 | 0.024 | 0.036 | 10.032 | <0.001 |
| UA | -0.007 | -0.012 | -0.003 | -3.381 | 0.012 |
| FS | -0.005 | -0.009 | -0.001 | -2.545 | 0.177 |

| Random effects | Estimate | Lower 95% CI | Upper 95% CI |
| --- | --- | --- | --- |
| (Intercept) | 0.010 | 0.006 | 0.015 |
| Error | 0.028 | 0.027 | 0.029 |

Post-hoc: UA vs. LT

| Fixed effects | Estimate | Lower 95% CI | Upper 95% CI | t | p |
| --- | --- | --- | --- | --- | --- |
| RA:LT | -0.002 | -0.015 | 0.011 | -0.247 | 0.805 |
| ES:LT | -0.005 | -0.018 | 0.008 | -0.762 | 0.446 |
| Pec:LT | -0.006 | -0.019 | 0.007 | -0.880 | 0.379 |
| Lat:LT | 0.003 | -0.010 | 0.017 | 0.516 | 0.606 |
| Tra:LT | 0.015 | 0.002 | 0.028 | 2.255 | 0.024 |
| Del:LT | 0.014 | 0.001 | 0.028 | 2.167 | 0.030 |
| Bic:LT | 0.001 | -0.012 | 0.014 | 0.182 | 0.856 |
| Tri:LT | 0.005 | -0.008 | 0.018 | 0.736 | 0.462 |
| GM:LT | -0.012 | -0.025 | 0.001 | -1.790 | 0.074 |
| TF:LT | 0.003 | -0.010 | 0.016 | 0.408 | 0.684 |
| RF:LT | 0.006 | -0.007 | 0.019 | 0.954 | 0.340 |
| VM:LT | 0.009 | -0.004 | 0.022 | 1.336 | 0.182 |
| BF:LT | -0.015 | -0.028 | -0.002 | -2.294 | 0.022 |
| TA:LT | 0.004 | -0.009 | 0.017 | 0.613 | 0.540 |
| PL:LT | -0.005 | -0.018 | 0.008 | -0.795 | 0.427 |

| Random effects | Estimate | Lower 95% CI | Upper 95% CI |
| --- | --- | --- | --- |
| (Intercept) | 0.010 | 0.007 | 0.015 |
| Error | 0.023 | 0.023 | 0.024 |

**Detailed statistics: stand-to-sit, non-challenging condition, hybrid strategy, synergy 10**

Reference group: UA

| Model information | Values |
| --- | --- |
| Number of observations | 1152 |
| Number of participants | 14 |
| ICC | 0.536 |

| Fixed effects | Estimate | Lower 95% CI | Upper 95% CI | t | p |
| --- | --- | --- | --- | --- | --- |
| (Intercept) | 0.022 | 0.016 | 0.027 | 8.094 | <0.001 |
| LT | 0.008 | 0.004 | 0.012 | 3.899 | 0.002 |
| FS | 0.004 | -0.001 | 0.008 | 1.592 | 1.000 |

| Random effects | Estimate | Lower 95% CI | Upper 95% CI |
| --- | --- | --- | --- |
| (Intercept) | 0.007 | 0.005 | 0.011 |
| Error | 0.027 | 0.026 | 0.028 |

Reference group: LT

| Fixed effects | Estimate | Lower 95% CI | Upper 95% CI | t | p |
| --- | --- | --- | --- | --- | --- |
| (Intercept) | 0.030 | 0.025 | 0.035 | 12.642 | <0.001 |
| UA | -0.008 | -0.012 | -0.004 | -3.899 | 0.002 |
| FS | -0.005 | -0.009 | -0.001 | -2.318 | 0.330 |

| Random effects | Estimate | Lower 95% CI | Upper 95% CI |
| --- | --- | --- | --- |
| (Intercept) | 0.007 | 0.005 | 0.011 |
| Error | 0.027 | 0.026 | 0.028 |

Post-hoc: UA vs. LT

| Fixed effects | Estimate | Lower 95% CI | Upper 95% CI | t | p |
| --- | --- | --- | --- | --- | --- |
| RA:LT | -0.002 | -0.014 | 0.010 | -0.293 | 0.770 |
| ES:LT | -0.010 | -0.022 | 0.002 | -1.709 | 0.088 |
| Pec:LT | 0.037 | 0.025 | 0.049 | 6.032 | <0.001 |
| Lat:LT | -0.005 | -0.017 | 0.007 | -0.815 | 0.415 |
| Tra:LT | 0.011 | -0.001 | 0.023 | 1.762 | 0.078 |
| Del:LT | 0.029 | 0.017 | 0.041 | 4.706 | <0.001 |
| Bic:LT | -0.004 | -0.016 | 0.008 | -0.715 | 0.475 |
| Tri:LT | 0.032 | 0.020 | 0.044 | 5.196 | <0.001 |
| GM:LT | -0.015 | -0.027 | -0.003 | -2.394 | 0.017 |
| TF:LT | -0.011 | -0.023 | 0.001 | -1.854 | 0.064 |
| RF:LT | -0.008 | -0.020 | 0.004 | -1.271 | 0.204 |
| VM:LT | -0.013 | -0.025 | -0.001 | -2.098 | 0.036 |
| BF:LT | -0.013 | -0.025 | -0.001 | -2.201 | 0.028 |
| TA:LT | -0.008 | -0.020 | 0.004 | -1.344 | 0.179 |
| PL:LT | -0.010 | -0.022 | 0.002 | -1.602 | 0.109 |

| Random effects | Estimate | Lower 95% CI | Upper 95% CI |
| --- | --- | --- | --- |
| (Intercept) | 0.008 | 0.005 | 0.012 |
| Error | 0.021 | 0.021 | 0.022 |

**Detailed statistics: stand-to-sit, non-challenging condition, vertical lowering strategy, synergy 1**

Reference group: UA

| Model information | Values |
| --- | --- |
| Number of observations | 864 |
| Number of participants | 12 |
| ICC | 0.674 |

| Fixed effects | Estimate | Lower 95% CI | Upper 95% CI | t | p |
| --- | --- | --- | --- | --- | --- |
| (Intercept) | 0.022 | 0.018 | 0.027 | 9.218 | <0.001 |
| LT | 0.001 | -0.003 | 0.005 | 0.444 | 1.000 |
| FS | 0.003 | -0.003 | 0.008 | 0.983 | 1.000 |

| Random effects | Estimate | Lower 95% CI | Upper 95% CI |
| --- | --- | --- | --- |
| (Intercept) | 0.008 | 0.005 | 0.012 |
| Error | 0.023 | 0.022 | 0.024 |

Reference group: LT

| Fixed effects | Estimate | Lower 95% CI | Upper 95% CI | t | p |
| --- | --- | --- | --- | --- | --- |
| (Intercept) | 0.023 | 0.018 | 0.029 | 8.453 | <0.001 |
| UA | -0.001 | -0.005 | 0.003 | -0.444 | 1.000 |
| FS | 0.002 | -0.004 | 0.008 | 0.665 | 1.000 |

| Random effects | Estimate | Lower 95% CI | Upper 95% CI |
| --- | --- | --- | --- |
| (Intercept) | 0.008 | 0.005 | 0.012 |
| Error | 0.023 | 0.022 | 0.024 |

**Detailed statistics: stand-to-sit, non-challenging condition, vertical lowering strategy, synergy 2**

Reference group: UA

| Model information | Values |
| --- | --- |
| Number of observations | 864 |
| Number of participants | 12 |
| ICC | 0.383 |

| Fixed effects | Estimate | Lower 95% CI | Upper 95% CI | t | p |
| --- | --- | --- | --- | --- | --- |
| (Intercept) | 0.025 | 0.021 | 0.028 | 13.619 | <0.001 |
| LT | 0.005 | 0.000 | 0.009 | 2.199 | 0.450 |
| FS | -0.004 | -0.010 | 0.003 | -1.154 | 1.000 |

| Random effects | Estimate | Lower 95% CI | Upper 95% CI |
| --- | --- | --- | --- |
| (Intercept) | 0.005 | 0.003 | 0.008 |
| Error | 0.025 | 0.024 | 0.026 |

Reference group: LT

| Fixed effects | Estimate | Lower 95% CI | Upper 95% CI | t | p |
| --- | --- | --- | --- | --- | --- |
| (Intercept) | 0.029 | 0.025 | 0.034 | 12.879 | <0.001 |
| UA | -0.005 | -0.009 | -0.000 | -2.199 | 0.450 |
| FS | -0.008 | -0.014 | -0.002 | -2.540 | 0.180 |

| Random effects | Estimate | Lower 95% CI | Upper 95% CI |
| --- | --- | --- | --- |
| (Intercept) | 0.005 | 0.003 | 0.008 |
| Error | 0.025 | 0.024 | 0.026 |

**Detailed statistics: stand-to-sit, non-challenging condition, vertical lowering strategy, synergy 3**

Reference group: UA

| Model information | Values |
| --- | --- |
| Number of observations | 864 |
| Number of participants | 12 |
| ICC | 0.321 |

| Fixed effects | Estimate | Lower 95% CI | Upper 95% CI | t | p |
| --- | --- | --- | --- | --- | --- |
| (Intercept) | 0.023 | 0.020 | 0.026 | 14.452 | <0.001 |
| LT | -0.001 | -0.005 | 0.003 | -0.369 | 1.000 |
| FS | 0.002 | -0.004 | 0.008 | 0.751 | 1.000 |

| Random effects | Estimate | Lower 95% CI | Upper 95% CI |
| --- | --- | --- | --- |
| (Intercept) | 0.004 | 0.002 | 0.008 |
| Error | 0.025 | 0.024 | 0.026 |

Reference group: LT

| Fixed effects | Estimate | Lower 95% CI | Upper 95% CI | t | p |
| --- | --- | --- | --- | --- | --- |
| (Intercept) | 0.022 | 0.018 | 0.026 | 10.817 | <0.001 |
| UA | 0.001 | -0.003 | 0.005 | 0.369 | 1.000 |
| FS | 0.003 | -0.003 | 0.009 | 0.960 | 1.000 |

| Random effects | Estimate | Lower 95% CI | Upper 95% CI |
| --- | --- | --- | --- |
| (Intercept) | 0.004 | 0.002 | 0.008 |
| Error | 0.025 | 0.024 | 0.026 |

**Detailed statistics: stand-to-sit, non-challenging condition, vertical lowering strategy, synergy 4**

Reference group: UA

| Model information | Values |
| --- | --- |
| Number of observations | 864 |
| Number of participants | 12 |
| ICC | 0.222 |

| Fixed effects | Estimate | Lower 95% CI | Upper 95% CI | t | p |
| --- | --- | --- | --- | --- | --- |
| (Intercept) | 0.023 | 0.021 | 0.025 | 18.385 | <0.001 |
| LT | -0.004 | -0.008 | -0.001 | -2.293 | 0.353 |
| FS | -0.006 | -0.011 | -0.000 | -2.058 | 0.639 |

| Random effects | Estimate | Lower 95% CI | Upper 95% CI |
| --- | --- | --- | --- |
| (Intercept) | 0.003 | 0.001 | 0.006 |
| Error | 0.023 | 0.022 | 0.024 |

Reference group: LT

| Fixed effects | Estimate | Lower 95% CI | Upper 95% CI | t | p |
| --- | --- | --- | --- | --- | --- |
| (Intercept) | 0.019 | 0.016 | 0.022 | 11.107 | <0.001 |
| UA | 0.004 | 0.001 | 0.008 | 2.293 | 0.353 |
| FS | -0.001 | -0.007 | 0.004 | -0.489 | 1.000 |

| Random effects | Estimate | Lower 95% CI | Upper 95% CI |
| --- | --- | --- | --- |
| (Intercept) | 0.003 | 0.001 | 0.006 |
| Error | 0.023 | 0.022 | 0.024 |

**Detailed statistics: stand-to-sit, non-challenging condition, vertical lowering strategy, synergy 5**

Reference group: UA

| Model information | Values |
| --- | --- |
| Number of observations | 864 |
| Number of participants | 12 |
| ICC | 0.261 |

| Fixed effects | Estimate | Lower 95% CI | Upper 95% CI | t | p |
| --- | --- | --- | --- | --- | --- |
| (Intercept) | 0.025 | 0.022 | 0.028 | 16.516 | <0.001 |
| LT | -0.000 | -0.004 | 0.004 | -0.180 | 1.000 |
| FS | -0.002 | -0.008 | 0.004 | -0.607 | 1.000 |

| Random effects | Estimate | Lower 95% CI | Upper 95% CI |
| --- | --- | --- | --- |
| (Intercept) | 0.003 | 0.001 | 0.007 |
| Error | 0.026 | 0.024 | 0.027 |

Reference group: LT

| Fixed effects | Estimate | Lower 95% CI | Upper 95% CI | t | p |
| --- | --- | --- | --- | --- | --- |
| (Intercept) | 0.024 | 0.020 | 0.028 | 12.222 | <0.001 |
| UA | 0.000 | -0.004 | 0.004 | 0.180 | 1.000 |
| FS | -0.001 | -0.008 | 0.005 | -0.464 | 1.000 |

| Random effects | Estimate | Lower 95% CI | Upper 95% CI |
| --- | --- | --- | --- |
| (Intercept) | 0.003 | 0.001 | 0.007 |
| Error | 0.026 | 0.024 | 0.027 |

**Detailed statistics: stand-to-sit, non-challenging condition, vertical lowering strategy, synergy 6**

Reference group: UA

| Model information | Values |
| --- | --- |
| Number of observations | 864 |
| Number of participants | 12 |
| ICC | 0.513 |

| Fixed effects | Estimate | Lower 95% CI | Upper 95% CI | t | p |
| --- | --- | --- | --- | --- | --- |
| (Intercept) | 0.022 | 0.018 | 0.026 | 10.413 | <0.001 |
| LT | 0.007 | 0.003 | 0.011 | 3.353 | 0.013 |
| FS | 0.002 | -0.004 | 0.008 | 0.639 | 1.000 |

| Random effects | Estimate | Lower 95% CI | Upper 95% CI |
| --- | --- | --- | --- |
| (Intercept) | 0.006 | 0.004 | 0.010 |
| Error | 0.024 | 0.023 | 0.025 |

Reference group: LT

| Fixed effects | Estimate | Lower 95% CI | Upper 95% CI | t | p |
| --- | --- | --- | --- | --- | --- |
| (Intercept) | 0.028 | 0.024 | 0.033 | 11.478 | <0.001 |
| UA | -0.007 | -0.011 | -0.003 | -3.353 | 0.013 |
| FS | -0.005 | -0.011 | 0.001 | -1.570 | 1.000 |

| Random effects | Estimate | Lower 95% CI | Upper 95% CI |
| --- | --- | --- | --- |
| (Intercept) | 0.006 | 0.004 | 0.010 |
| Error | 0.024 | 0.023 | 0.025 |

Post-hoc: UA vs. LT

| Fixed effects | Estimate | Lower 95% CI | Upper 95% CI | t | p |
| --- | --- | --- | --- | --- | --- |
| RA:LT | 0.003 | -0.008 | 0.014 | 0.587 | 0.557 |
| ES:LT | -0.018 | -0.029 | -0.007 | -3.162 | 0.002 |
| Pec:LT | -0.005 | -0.016 | 0.006 | -0.894 | 0.372 |
| Lat:LT | -0.002 | -0.013 | 0.009 | -0.397 | 0.692 |
| Tra:LT | 0.016 | 0.005 | 0.027 | 2.892 | 0.004 |
| Del:LT | 0.022 | 0.011 | 0.033 | 3.962 | <0.001 |
| Bic:LT | 0.000 | -0.011 | 0.011 | 0.058 | 0.954 |
| Tri:LT | 0.026 | 0.015 | 0.037 | 4.619 | <0.001 |
| GM:LT | -0.006 | -0.017 | 0.005 | -1.130 | 0.259 |
| TF:LT | -0.006 | -0.017 | 0.005 | -1.014 | 0.311 |
| RF:LT | -0.005 | -0.016 | 0.006 | -0.875 | 0.382 |
| VM:LT | -0.002 | -0.013 | 0.009 | -0.385 | 0.700 |
| BF:LT | -0.007 | -0.018 | 0.004 | -1.303 | 0.193 |
| TA:LT | -0.005 | -0.016 | 0.006 | -0.871 | 0.384 |
| PL:LT | -0.004 | -0.015 | 0.007 | -0.794 | 0.427 |

| Random effects | Estimate | Lower 95% CI | Upper 95% CI |
| --- | --- | --- | --- |
| (Intercept) | 0.007 | 0.004 | 0.011 |
| Error | 0.019 | 0.018 | 0.020 |

**Detailed statistics: stand-to-sit, non-challenging condition, vertical lowering strategy, synergy 8**

Reference group: UA

| Model information | Values |
| --- | --- |
| Number of observations | 864 |
| Number of participants | 12 |
| ICC | 0.672 |

| Fixed effects | Estimate | Lower 95% CI | Upper 95% CI | t | p |
| --- | --- | --- | --- | --- | --- |
| (Intercept) | 0.017 | 0.013 | 0.022 | 7.464 | <0.001 |
| LT | 0.011 | 0.007 | 0.015 | 6.036 | <0.001 |
| FS | 0.018 | 0.012 | 0.023 | 6.480 | <0.001 |

| Random effects | Estimate | Lower 95% CI | Upper 95% CI |
| --- | --- | --- | --- |
| (Intercept) | 0.007 | 0.005 | 0.012 |
| Error | 0.022 | 0.021 | 0.023 |

Reference group: LT

| Fixed effects | Estimate | Lower 95% CI | Upper 95% CI | t | p |
| --- | --- | --- | --- | --- | --- |
| (Intercept) | 0.028 | 0.023 | 0.033 | 10.791 | <0.001 |
| UA | -0.011 | -0.015 | -0.007 | -6.036 | <0.001 |
| FS | 0.007 | 0.001 | 0.012 | 2.343 | 0.309 |

| Random effects | Estimate | Lower 95% CI | Upper 95% CI |
| --- | --- | --- | --- |
| (Intercept) | 0.007 | 0.005 | 0.012 |
| Error | 0.022 | 0.021 | 0.023 |

Post-hoc: UA vs. LT

| Fixed effects | Estimate | Lower 95% CI | Upper 95% CI | t | p |
| --- | --- | --- | --- | --- | --- |
| RA:LT | 0.008 | -0.004 | 0.019 | 1.330 | 0.368 |
| ES:LT | -0.011 | -0.022 | 0.001 | -1.852 | 0.129 |
| Pec:LT | -0.011 | -0.022 | 0.000 | -1.937 | 0.106 |
| Lat:LT | -0.001 | -0.012 | 0.010 | -0.216 | 1.000 |
| Tra:LT | 0.020 | 0.009 | 0.031 | 3.539 | 0.001 |
| Del:LT | 0.021 | 0.010 | 0.032 | 3.642 | 0.001 |
| Bic:LT | -0.004 | -0.015 | 0.007 | -0.691 | 0.979 |
| Tri:LT | 0.024 | 0.013 | 0.036 | 4.248 | <0.001 |
| GM:LT | -0.006 | -0.017 | 0.005 | -1.042 | 0.595 |
| TF:LT | -0.004 | -0.016 | 0.007 | -0.778 | 0.873 |
| RF:LT | -0.000 | -0.012 | 0.011 | -0.056 | 1.000 |
| VM:LT | -0.003 | -0.014 | 0.009 | -0.454 | 1.000 |
| BF:LT | -0.004 | -0.015 | 0.007 | -0.685 | 0.987 |
| TA:LT | -0.013 | -0.024 | -0.002 | -2.317 | 0.042 |
| PL:LT | -0.010 | -0.021 | 0.001 | -1.721 | 0.171 |

| Random effects | Estimate | Lower 95% CI | Upper 95% CI |
| --- | --- | --- | --- |
| (Intercept) | 0.007 | 0.005 | 0.012 |
| Error | 0.019 | 0.018 | 0.020 |

Post-hoc: UA vs. FS

| Fixed effects | Estimate | Lower 95% CI | Upper 95% CI | t | p |
| --- | --- | --- | --- | --- | --- |
| RA:FS | 0.007 | -0.010 | 0.023 | 0.802 | 0.846 |
| ES:FS | -0.026 | -0.042 | -0.010 | -3.124 | 0.004 |
| Pec:FS | 0.030 | 0.014 | 0.046 | 3.599 | 0.001 |
| Lat:FS | -0.008 | -0.024 | 0.008 | -0.959 | 0.675 |
| Tra:FS | 0.049 | 0.033 | 0.065 | 5.878 | <0.001 |
| Del:FS | -0.002 | -0.018 | 0.014 | -0.239 | 1.000 |
| Bic:FS | 0.004 | -0.013 | 0.020 | 0.422 | 1.000 |
| Tri:FS | 0.010 | -0.006 | 0.027 | 1.227 | 0.440 |
| GM:FS | -0.007 | -0.023 | 0.009 | -0.829 | 0.815 |
| TF:FS | -0.004 | -0.020 | 0.012 | -0.491 | 1.000 |
| RF:FS | 0.012 | -0.005 | 0.028 | 1.389 | 0.330 |
| VM:FS | -0.011 | -0.028 | 0.005 | -1.359 | 0.349 |
| BF:FS | -0.001 | -0.018 | 0.015 | -0.154 | 1.000 |
| TA:FS | -0.013 | -0.030 | 0.003 | -1.590 | 0.224 |
| PL:FS | -0.016 | -0.033 | -0.000 | -1.976 | 0.097 |

| Random effects | Estimate | Lower 95% CI | Upper 95% CI |
| --- | --- | --- | --- |
| (Intercept) | 0.007 | 0.005 | 0.012 |
| Error | 0.019 | 0.018 | 0.020 |

**Detailed statistics: stand-to-sit, non-challenging condition, vertical lowering strategy, synergy 10**

Reference group: UA

| Model information | Values |
| --- | --- |
| Number of observations | 864 |
| Number of participants | 12 |
| ICC | 0.377 |

| Fixed effects | Estimate | Lower 95% CI | Upper 95% CI | t | p |
| --- | --- | --- | --- | --- | --- |
| (Intercept) | 0.018 | 0.014 | 0.022 | 8.754 | <0.001 |
| LT | 0.009 | 0.004 | 0.013 | 3.617 | 0.005 |
| FS | 0.011 | 0.004 | 0.018 | 3.053 | 0.037 |

| Random effects | Estimate | Lower 95% CI | Upper 95% CI |
| --- | --- | --- | --- |
| (Intercept) | 0.005 | 0.003 | 0.009 |
| Error | 0.029 | 0.028 | 0.030 |

Reference group: LT

| Fixed effects | Estimate | Lower 95% CI | Upper 95% CI | t | p |
| --- | --- | --- | --- | --- | --- |
| (Intercept) | 0.027 | 0.022 | 0.032 | 10.309 | <0.001 |
| UA | -0.009 | -0.013 | -0.004 | -3.617 | 0.005 |
| FS | 0.002 | -0.005 | 0.009 | 0.598 | 1.000 |

| Random effects | Estimate | Lower 95% CI | Upper 95% CI |
| --- | --- | --- | --- |
| (Intercept) | 0.005 | 0.003 | 0.009 |
| Error | 0.029 | 0.028 | 0.030 |

Post-hoc: UA vs. LT

| Fixed effects | Estimate | Lower 95% CI | Upper 95% CI | t | p |
| --- | --- | --- | --- | --- | --- |
| RA:LT | -0.003 | -0.015 | 0.009 | -0.548 | 1.000 |
| ES:LT | -0.009 | -0.021 | 0.003 | -1.483 | 0.277 |
| Pec:LT | 0.025 | 0.013 | 0.037 | 4.116 | <0.001 |
| Lat:LT | -0.009 | -0.021 | 0.003 | -1.485 | 0.276 |
| Tra:LT | 0.009 | -0.003 | 0.021 | 1.496 | 0.270 |
| Del:LT | 0.030 | 0.018 | 0.042 | 4.991 | <0.001 |
| Bic:LT | -0.011 | -0.023 | 0.000 | -1.883 | 0.120 |
| Tri:LT | 0.053 | 0.041 | 0.064 | 8.735 | <0.001 |
| GM:LT | -0.013 | -0.025 | -0.001 | -2.202 | 0.056 |
| TF:LT | -0.017 | -0.029 | -0.005 | -2.827 | 0.010 |
| RF:LT | -0.003 | -0.015 | 0.009 | -0.537 | 1.000 |
| VM:LT | -0.008 | -0.020 | 0.004 | -1.342 | 0.360 |
| BF:LT | -0.008 | -0.020 | 0.004 | -1.300 | 0.388 |
| TA:LT | -0.016 | -0.027 | -0.004 | -2.589 | 0.020 |
| PL:LT | -0.011 | -0.023 | 0.000 | -1.898 | 0.116 |

| Random effects | Estimate | Lower 95% CI | Upper 95% CI |
| --- | --- | --- | --- |
| (Intercept) | 0.006 | 0.004 | 0.009 |
| Error | 0.020 | 0.019 | 0.021 |

Post-hoc: UA vs. FS

| Fixed effects | Estimate | Lower 95% CI | Upper 95% CI | t | p |
| --- | --- | --- | --- | --- | --- |
| RA:FS | 0.025 | 0.008 | 0.042 | 2.879 | 0.008 |
| ES:FS | -0.010 | -0.028 | 0.007 | -1.199 | 0.462 |
| Pec:FS | 0.078 | 0.060 | 0.095 | 8.864 | <0.001 |
| Lat:FS | -0.010 | -0.027 | 0.007 | -1.110 | 0.534 |
| Tra:FS | 0.033 | 0.016 | 0.050 | 3.778 | <0.001 |
| Del:FS | 0.007 | -0.010 | 0.025 | 0.853 | 0.787 |
| Bic:FS | -0.018 | -0.035 | -0.001 | -2.081 | 0.076 |
| Tri:FS | 0.016 | -0.001 | 0.033 | 1.799 | 0.145 |
| GM:FS | -0.020 | -0.037 | -0.003 | -2.289 | 0.045 |
| TF:FS | -0.017 | -0.035 | -0.000 | -1.987 | 0.095 |
| RF:FS | -0.002 | -0.020 | 0.015 | -0.279 | 1.000 |
| VM:FS | -0.011 | -0.028 | 0.006 | -1.286 | 0.398 |
| BF:FS | -0.013 | -0.031 | 0.004 | -1.525 | 0.256 |
| TA:FS | -0.024 | -0.041 | -0.007 | -2.775 | 0.011 |
| PL:FS | -0.017 | -0.034 | 0.000 | -1.914 | 0.112 |

| Random effects | Estimate | Lower 95% CI | Upper 95% CI |
| --- | --- | --- | --- |
| (Intercept) | 0.006 | 0.004 | 0.009 |
| Error | 0.020 | 0.019 | 0.021 |

## Stand-to-sit, challenging condition

**Detailed statistics: stand-to-sit, challenging condition, exaggerated forward leaning strategy, synergy 1**

Reference group: UA

| Model information | Values |
| --- | --- |
| Number of observations | 1200 |
| Number of participants | 17 |
| ICC | 0.591 |

| Fixed effects | Estimate | Lower 95% CI | Upper 95% CI | t | p |
| --- | --- | --- | --- | --- | --- |
| (Intercept) | 0.034 | 0.029 | 0.038 | 14.297 | <0.001 |
| LT | -0.007 | -0.011 | -0.004 | -4.019 | 0.001 |
| FS | -0.007 | -0.012 | -0.002 | -2.607 | 0.129 |

| Random effects | Estimate | Lower 95% CI | Upper 95% CI |
| --- | --- | --- | --- |
| (Intercept) | 0.008 | 0.005 | 0.012 |
| Error | 0.028 | 0.027 | 0.029 |

Reference group: LT

| Fixed effects | Estimate | Lower 95% CI | Upper 95% CI | t | p |
| --- | --- | --- | --- | --- | --- |
| (Intercept) | 0.026 | 0.022 | 0.031 | 10.759 | <0.001 |
| UA | 0.007 | 0.004 | 0.011 | 4.019 | 0.001 |
| FS | 0.001 | -0.004 | 0.006 | 0.265 | 1.000 |

| Random effects | Estimate | Lower 95% CI | Upper 95% CI |
| --- | --- | --- | --- |
| (Intercept) | 0.008 | 0.005 | 0.012 |
| Error | 0.028 | 0.027 | 0.029 |

Post-hoc: UA vs. LT

| Fixed effects | Estimate | Lower 95% CI | Upper 95% CI | t | p |
| --- | --- | --- | --- | --- | --- |
| RA:LT | 0.013 | 0.002 | 0.024 | 2.329 | 0.020 |
| ES:LT | -0.000 | -0.011 | 0.011 | -0.044 | 0.965 |
| Pec:LT | -0.001 | -0.012 | 0.010 | -0.212 | 0.832 |
| Lat:LT | 0.016 | 0.005 | 0.027 | 2.927 | 0.003 |
| Tra:LT | 0.004 | -0.007 | 0.015 | 0.769 | 0.442 |
| Del:LT | 0.013 | 0.002 | 0.024 | 2.264 | 0.024 |
| Bic:LT | 0.010 | -0.001 | 0.021 | 1.790 | 0.074 |
| Tri:LT | 0.017 | 0.006 | 0.028 | 3.037 | 0.002 |
| GM:LT | -0.009 | -0.020 | 0.002 | -1.601 | 0.110 |
| TF:LT | 0.006 | -0.005 | 0.017 | 1.012 | 0.312 |
| RF:LT | -0.003 | -0.014 | 0.008 | -0.529 | 0.597 |
| VM:LT | -0.001 | -0.012 | 0.010 | -0.192 | 0.848 |
| BF:LT | -0.009 | -0.020 | 0.002 | -1.546 | 0.122 |
| TA:LT | -0.012 | -0.023 | -0.001 | -2.056 | 0.040 |
| PL:LT | -0.010 | -0.021 | 0.001 | -1.853 | 0.064 |

| Random effects | Estimate | Lower 95% CI | Upper 95% CI |
| --- | --- | --- | --- |
| (Intercept) | 0.009 | 0.006 | 0.013 |
| Error | 0.023 | 0.022 | 0.024 |

**Detailed statistics: stand-to-sit, challenging condition, exaggerated forward leaning strategy, synergy 2**

Reference group: UA

| Model information | Values |
| --- | --- |
| Number of observations | 1200 |
| Number of participants | 17 |
| ICC | 0.426 |

| Fixed effects | Estimate | Lower 95% CI | Upper 95% CI | t | p |
| --- | --- | --- | --- | --- | --- |
| (Intercept) | 0.034 | 0.029 | 0.038 | 15.929 | <0.001 |
| LT | -0.005 | -0.009 | -0.001 | -2.285 | 0.315 |
| FS | -0.003 | -0.008 | 0.002 | -1.081 | 1.000 |

| Random effects | Estimate | Lower 95% CI | Upper 95% CI |
| --- | --- | --- | --- |
| (Intercept) | 0.007 | 0.004 | 0.010 |
| Error | 0.031 | 0.030 | 0.032 |

Reference group: LT

| Fixed effects | Estimate | Lower 95% CI | Upper 95% CI | t | p |
| --- | --- | --- | --- | --- | --- |
| (Intercept) | 0.029 | 0.025 | 0.033 | 13.014 | <0.001 |
| UA | 0.005 | 0.001 | 0.009 | 2.285 | 0.315 |
| FS | 0.002 | -0.004 | 0.007 | 0.560 | 1.000 |

| Random effects | Estimate | Lower 95% CI | Upper 95% CI |
| --- | --- | --- | --- |
| (Intercept) | 0.007 | 0.004 | 0.010 |
| Error | 0.031 | 0.030 | 0.032 |

**Detailed statistics: stand-to-sit, challenging condition, exaggerated forward leaning strategy, synergy 3**

Reference group: UA

| Model information | Values |
| --- | --- |
| Number of observations | 1200 |
| Number of participants | 17 |
| ICC | 0.554 |

| Fixed effects | Estimate | Lower 95% CI | Upper 95% CI | t | p |
| --- | --- | --- | --- | --- | --- |
| (Intercept) | 0.035 | 0.030 | 0.040 | 15.109 | <0.001 |
| LT | -0.003 | -0.007 | 0.001 | -1.651 | 1.000 |
| FS | 0.002 | -0.003 | 0.007 | 0.794 | 1.000 |

| Random effects | Estimate | Lower 95% CI | Upper 95% CI |
| --- | --- | --- | --- |
| (Intercept) | 0.008 | 0.005 | 0.012 |
| Error | 0.029 | 0.028 | 0.030 |

Reference group: LT

| Fixed effects | Estimate | Lower 95% CI | Upper 95% CI | t | p |
| --- | --- | --- | --- | --- | --- |
| (Intercept) | 0.032 | 0.027 | 0.037 | 13.210 | <0.001 |
| UA | 0.003 | -0.001 | 0.007 | 1.651 | 1.000 |
| FS | 0.005 | -0.000 | 0.010 | 1.933 | 0.749 |

| Random effects | Estimate | Lower 95% CI | Upper 95% CI |
| --- | --- | --- | --- |
| (Intercept) | 0.008 | 0.005 | 0.012 |
| Error | 0.029 | 0.028 | 0.030 |

**Detailed statistics: stand-to-sit, challenging condition, exaggerated forward leaning strategy, synergy 4**

Reference group: UA

| Model information | Values |
| --- | --- |
| Number of observations | 1200 |
| Number of participants | 17 |
| ICC | 0.694 |

| Fixed effects | Estimate | Lower 95% CI | Upper 95% CI | t | p |
| --- | --- | --- | --- | --- | --- |
| (Intercept) | 0.025 | 0.021 | 0.030 | 11.068 | <0.001 |
| LT | 0.000 | -0.003 | 0.003 | 0.212 | 1.000 |
| FS | 0.002 | -0.002 | 0.007 | 1.088 | 1.000 |

| Random effects | Estimate | Lower 95% CI | Upper 95% CI |
| --- | --- | --- | --- |
| (Intercept) | 0.008 | 0.006 | 0.012 |
| Error | 0.024 | 0.023 | 0.025 |

Reference group: LT

| Fixed effects | Estimate | Lower 95% CI | Upper 95% CI | t | p |
| --- | --- | --- | --- | --- | --- |
| (Intercept) | 0.026 | 0.021 | 0.030 | 10.869 | <0.001 |
| UA | -0.000 | -0.003 | 0.003 | -0.212 | 1.000 |
| FS | 0.002 | -0.002 | 0.006 | 0.916 | 1.000 |

| Random effects | Estimate | Lower 95% CI | Upper 95% CI |
| --- | --- | --- | --- |
| (Intercept) | 0.008 | 0.006 | 0.012 |
| Error | 0.024 | 0.023 | 0.025 |

**Detailed statistics: stand-to-sit, challenging condition, exaggerated forward leaning strategy, synergy 5**

Reference group: UA

| Model information | Values |
| --- | --- |
| Number of observations | 1200 |
| Number of participants | 17 |
| ICC | 0.672 |

| Fixed effects | Estimate | Lower 95% CI | Upper 95% CI | t | p |
| --- | --- | --- | --- | --- | --- |
| (Intercept) | 0.026 | 0.021 | 0.031 | 10.766 | <0.001 |
| LT | 0.003 | -0.000 | 0.007 | 1.948 | 0.723 |
| FS | 0.002 | -0.002 | 0.007 | 1.005 | 1.000 |

| Random effects | Estimate | Lower 95% CI | Upper 95% CI |
| --- | --- | --- | --- |
| (Intercept) | 0.009 | 0.006 | 0.013 |
| Error | 0.026 | 0.025 | 0.027 |

Reference group: LT

| Fixed effects | Estimate | Lower 95% CI | Upper 95% CI | t | p |
| --- | --- | --- | --- | --- | --- |
| (Intercept) | 0.029 | 0.024 | 0.034 | 11.749 | <0.001 |
| UA | -0.003 | -0.007 | 0.000 | -1.948 | 0.723 |
| FS | -0.001 | -0.006 | 0.004 | -0.378 | 1.000 |

| Random effects | Estimate | Lower 95% CI | Upper 95% CI |
| --- | --- | --- | --- |
| (Intercept) | 0.009 | 0.006 | 0.013 |
| Error | 0.026 | 0.025 | 0.027 |

**Detailed statistics: stand-to-sit, challenging condition, exaggerated forward leaning strategy, synergy 6**

Reference group: UA

| Model information | Values |
| --- | --- |
| Number of observations | 1200 |
| Number of participants | 17 |
| ICC | 0.680 |

| Fixed effects | Estimate | Lower 95% CI | Upper 95% CI | t | p |
| --- | --- | --- | --- | --- | --- |
| (Intercept) | 0.023 | 0.018 | 0.028 | 9.766 | <0.001 |
| LT | 0.005 | 0.002 | 0.009 | 3.322 | 0.013 |
| FS | 0.008 | 0.004 | 0.013 | 3.565 | 0.005 |

| Random effects | Estimate | Lower 95% CI | Upper 95% CI |
| --- | --- | --- | --- |
| (Intercept) | 0.009 | 0.006 | 0.013 |
| Error | 0.025 | 0.024 | 0.026 |

Reference group: LT

| Fixed effects | Estimate | Lower 95% CI | Upper 95% CI | t | p |
| --- | --- | --- | --- | --- | --- |
| (Intercept) | 0.028 | 0.024 | 0.033 | 11.705 | <0.001 |
| UA | -0.005 | -0.009 | -0.002 | -3.322 | 0.013 |
| FS | 0.003 | -0.002 | 0.007 | 1.166 | 1.000 |

| Random effects | Estimate | Lower 95% CI | Upper 95% CI |
| --- | --- | --- | --- |
| (Intercept) | 0.009 | 0.006 | 0.013 |
| Error | 0.025 | 0.024 | 0.026 |

Post-hoc: UA vs. LT

| Fixed effects | Estimate | Lower 95% CI | Upper 95% CI | t | p |
| --- | --- | --- | --- | --- | --- |
| RA:LT | -0.012 | -0.023 | -0.001 | -2.176 | 0.059 |
| ES:LT | -0.012 | -0.023 | -0.001 | -2.228 | 0.052 |
| Pec:LT | -0.007 | -0.018 | 0.003 | -1.359 | 0.349 |
| Lat:LT | -0.001 | -0.011 | 0.010 | -0.122 | 1.000 |
| Tra:LT | 0.020 | 0.010 | 0.031 | 3.737 | <0.001 |
| Del:LT | 0.024 | 0.014 | 0.035 | 4.465 | <0.001 |
| Bic:LT | -0.008 | -0.019 | 0.003 | -1.475 | 0.281 |
| Tri:LT | 0.017 | 0.007 | 0.028 | 3.200 | 0.003 |
| GM:LT | -0.016 | -0.027 | -0.005 | -2.955 | 0.006 |
| TF:LT | 0.005 | -0.005 | 0.016 | 0.964 | 0.671 |
| RF:LT | 0.002 | -0.009 | 0.013 | 0.327 | 1.000 |
| VM:LT | 0.000 | -0.011 | 0.011 | 0.034 | 1.000 |
| BF:LT | -0.009 | -0.020 | 0.002 | -1.671 | 0.190 |
| TA:LT | 0.004 | -0.007 | 0.014 | 0.663 | 1.000 |
| PL:LT | -0.002 | -0.013 | 0.008 | -0.416 | 1.000 |

| Random effects | Estimate | Lower 95% CI | Upper 95% CI |
| --- | --- | --- | --- |
| (Intercept) | 0.009 | 0.006 | 0.013 |
| Error | 0.022 | 0.021 | 0.023 |

Post-hoc: UA vs. FS

| Fixed effects | Estimate | Lower 95% CI | Upper 95% CI | t | p |
| --- | --- | --- | --- | --- | --- |
| RA:FS | -0.026 | -0.040 | -0.013 | -3.765 | <0.001 |
| ES:FS | -0.019 | -0.033 | -0.005 | -2.717 | 0.013 |
| Pec:FS | 0.021 | 0.007 | 0.035 | 3.028 | 0.005 |
| Lat:FS | -0.003 | -0.017 | 0.010 | -0.469 | 1.000 |
| Tra:FS | 0.038 | 0.025 | 0.052 | 5.485 | <0.001 |
| Del:FS | 0.024 | 0.010 | 0.038 | 3.438 | 0.001 |
| Bic:FS | -0.006 | -0.020 | 0.007 | -0.925 | 0.711 |
| Tri:FS | 0.022 | 0.008 | 0.035 | 3.098 | 0.004 |
| GM:FS | -0.016 | -0.030 | -0.003 | -2.337 | 0.039 |
| TF:FS | -0.007 | -0.020 | 0.007 | -0.937 | 0.698 |
| RF:FS | -0.006 | -0.020 | 0.007 | -0.912 | 0.724 |
| VM:FS | -0.000 | -0.014 | 0.014 | -0.015 | 1.000 |
| BF:FS | -0.010 | -0.024 | 0.004 | -1.412 | 0.317 |
| TA:FS | 0.006 | -0.008 | 0.020 | 0.874 | 0.764 |
| PL:FS | -0.005 | -0.018 | 0.009 | -0.684 | 0.988 |

| Random effects | Estimate | Lower 95% CI | Upper 95% CI |
| --- | --- | --- | --- |
| (Intercept) | 0.009 | 0.006 | 0.013 |
| Error | 0.022 | 0.021 | 0.023 |

**Detailed statistics: stand-to-sit, challenging condition, exaggerated forward leaning strategy, synergy 8**

Reference group: UA

| Model information | Values |
| --- | --- |
| Number of observations | 1200 |
| Number of participants | 17 |
| ICC | 0.383 |

| Fixed effects | Estimate | Lower 95% CI | Upper 95% CI | t | p |
| --- | --- | --- | --- | --- | --- |
| (Intercept) | 0.021 | 0.017 | 0.025 | 10.160 | <0.001 |
| LT | 0.009 | 0.005 | 0.013 | 4.217 | <0.001 |
| FS | 0.004 | -0.001 | 0.010 | 1.518 | 1.000 |

| Random effects | Estimate | Lower 95% CI | Upper 95% CI |
| --- | --- | --- | --- |
| (Intercept) | 0.006 | 0.004 | 0.010 |
| Error | 0.032 | 0.030 | 0.033 |

Reference group: LT

| Fixed effects | Estimate | Lower 95% CI | Upper 95% CI | t | p |
| --- | --- | --- | --- | --- | --- |
| (Intercept) | 0.029 | 0.025 | 0.034 | 13.597 | <0.001 |
| UA | -0.009 | -0.013 | -0.005 | -4.217 | <0.001 |
| FS | -0.004 | -0.010 | 0.001 | -1.510 | 1.000 |

| Random effects | Estimate | Lower 95% CI | Upper 95% CI |
| --- | --- | --- | --- |
| (Intercept) | 0.006 | 0.004 | 0.010 |
| Error | 0.032 | 0.030 | 0.033 |

Post-hoc: UA vs. LT

| Fixed effects | Estimate | Lower 95% CI | Upper 95% CI | t | p |
| --- | --- | --- | --- | --- | --- |
| RA:LT | -0.018 | -0.030 | -0.006 | -2.924 | 0.004 |
| ES:LT | -0.006 | -0.018 | 0.006 | -0.940 | 0.347 |
| Pec:LT | 0.035 | 0.023 | 0.047 | 5.735 | <0.001 |
| Lat:LT | -0.004 | -0.016 | 0.009 | -0.581 | 0.562 |
| Tra:LT | 0.008 | -0.004 | 0.020 | 1.338 | 0.181 |
| Del:LT | 0.048 | 0.036 | 0.060 | 7.771 | <0.001 |
| Bic:LT | -0.014 | -0.026 | -0.002 | -2.320 | 0.021 |
| Tri:LT | 0.030 | 0.018 | 0.042 | 4.886 | <0.001 |
| GM:LT | -0.009 | -0.021 | 0.003 | -1.457 | 0.145 |
| TF:LT | -0.014 | -0.026 | -0.002 | -2.309 | 0.021 |
| RF:LT | -0.007 | -0.019 | 0.005 | -1.142 | 0.254 |
| VM:LT | -0.006 | -0.018 | 0.006 | -0.957 | 0.339 |
| BF:LT | -0.008 | -0.020 | 0.004 | -1.357 | 0.175 |
| TA:LT | -0.016 | -0.028 | -0.004 | -2.624 | 0.009 |
| PL:LT | -0.009 | -0.022 | 0.003 | -1.542 | 0.123 |

| Random effects | Estimate | Lower 95% CI | Upper 95% CI |
| --- | --- | --- | --- |
| (Intercept) | 0.007 | 0.004 | 0.010 |
| Error | 0.025 | 0.024 | 0.026 |

**Detailed statistics: stand-to-sit, challenging condition, forward leaning strategy, synergy 1**

Reference group: UA

| Model information | Values |
| --- | --- |
| Number of observations | 1616 |
| Number of participants | 18 |
| ICC | 0.510 |

| Fixed effects | Estimate | Lower 95% CI | Upper 95% CI | t | p |
| --- | --- | --- | --- | --- | --- |
| (Intercept) | 0.042 | 0.037 | 0.048 | 15.997 | <0.001 |
| LT | -0.010 | -0.014 | -0.005 | -4.201 | <0.001 |
| FS | -0.014 | -0.018 | -0.010 | -6.400 | <0.001 |

| Random effects | Estimate | Lower 95% CI | Upper 95% CI |
| --- | --- | --- | --- |
| (Intercept) | 0.008 | 0.005 | 0.012 |
| Error | 0.032 | 0.031 | 0.033 |

Reference group: LT

| Fixed effects | Estimate | Lower 95% CI | Upper 95% CI | t | p |
| --- | --- | --- | --- | --- | --- |
| (Intercept) | 0.033 | 0.028 | 0.038 | 13.316 | <0.001 |
| UA | 0.010 | 0.005 | 0.014 | 4.201 | <0.001 |
| FS | -0.004 | -0.008 | -0.000 | -2.153 | 0.440 |

| Random effects | Estimate | Lower 95% CI | Upper 95% CI |
| --- | --- | --- | --- |
| (Intercept) | 0.008 | 0.005 | 0.012 |
| Error | 0.032 | 0.031 | 0.033 |

Post-hoc: UA vs. LT

| Fixed effects | Estimate | Lower 95% CI | Upper 95% CI | t | p |
| --- | --- | --- | --- | --- | --- |
| RA:LT | 0.007 | -0.008 | 0.022 | 0.893 | 0.744 |
| ES:LT | 0.007 | -0.008 | 0.023 | 0.958 | 0.676 |
| Pec:LT | -0.003 | -0.018 | 0.013 | -0.327 | 1.000 |
| Lat:LT | 0.017 | 0.001 | 0.032 | 2.141 | 0.065 |
| Tra:LT | 0.002 | -0.013 | 0.017 | 0.239 | 1.000 |
| Del:LT | 0.014 | -0.001 | 0.030 | 1.860 | 0.126 |
| Bic:LT | 0.006 | -0.009 | 0.021 | 0.746 | 0.911 |
| Tri:LT | 0.021 | 0.006 | 0.036 | 2.736 | 0.013 |
| GM:LT | -0.005 | -0.020 | 0.011 | -0.603 | 1.000 |
| TF:LT | -0.004 | -0.019 | 0.011 | -0.480 | 1.000 |
| RF:LT | 0.001 | -0.014 | 0.017 | 0.182 | 1.000 |
| VM:LT | 0.008 | -0.008 | 0.023 | 0.980 | 0.655 |
| BF:LT | 0.000 | -0.015 | 0.015 | 0.029 | 1.000 |
| TA:LT | -0.012 | -0.027 | 0.003 | -1.586 | 0.226 |
| PL:LT | -0.026 | -0.041 | -0.011 | -3.358 | 0.002 |

| Random effects | Estimate | Lower 95% CI | Upper 95% CI |
| --- | --- | --- | --- |
| (Intercept) | 0.008 | 0.006 | 0.013 |
| Error | 0.029 | 0.028 | 0.030 |

Post-hoc: UA vs. FS

| Fixed effects | Estimate | Lower 95% CI | Upper 95% CI | t | p |
| --- | --- | --- | --- | --- | --- |
| RA:FS | 0.017 | 0.003 | 0.031 | 2.397 | 0.033 |
| ES:FS | 0.003 | -0.011 | 0.017 | 0.458 | 1.000 |
| Pec:FS | 0.006 | -0.008 | 0.020 | 0.806 | 0.840 |
| Lat:FS | 0.019 | 0.005 | 0.033 | 2.622 | 0.018 |
| Tra:FS | 0.003 | -0.011 | 0.017 | 0.483 | 1.000 |
| Del:FS | -0.007 | -0.021 | 0.007 | -1.009 | 0.626 |
| Bic:FS | 0.019 | 0.005 | 0.033 | 2.634 | 0.017 |
| Tri:FS | 0.034 | 0.020 | 0.048 | 4.723 | <0.001 |
| GM:FS | 0.002 | -0.012 | 0.016 | 0.299 | 1.000 |
| TF:FS | -0.008 | -0.022 | 0.006 | -1.068 | 0.572 |
| RF:FS | -0.006 | -0.020 | 0.009 | -0.771 | 0.881 |
| VM:FS | -0.001 | -0.016 | 0.013 | -0.207 | 1.000 |
| BF:FS | -0.002 | -0.017 | 0.012 | -0.348 | 1.000 |
| TA:FS | -0.035 | -0.049 | -0.021 | -4.935 | <0.001 |
| PL:FS | -0.026 | -0.040 | -0.012 | -3.651 | 0.001 |

| Random effects | Estimate | Lower 95% CI | Upper 95% CI |
| --- | --- | --- | --- |
| (Intercept) | 0.008 | 0.006 | 0.013 |
| Error | 0.029 | 0.028 | 0.030 |

**Detailed statistics: stand-to-sit, challenging condition, forward leaning strategy, synergy 2**

Reference group: UA

| Model information | Values |
| --- | --- |
| Number of observations | 1616 |
| Number of participants | 18 |
| ICC | 0.488 |

| Fixed effects | Estimate | Lower 95% CI | Upper 95% CI | t | p |
| --- | --- | --- | --- | --- | --- |
| (Intercept) | 0.034 | 0.029 | 0.039 | 13.269 | <0.001 |
| LT | -0.001 | -0.006 | 0.003 | -0.581 | 1.000 |
| FS | -0.003 | -0.007 | 0.001 | -1.313 | 1.000 |

| Random effects | Estimate | Lower 95% CI | Upper 95% CI |
| --- | --- | --- | --- |
| (Intercept) | 0.008 | 0.005 | 0.012 |
| Error | 0.032 | 0.030 | 0.033 |

Reference group: LT

| Fixed effects | Estimate | Lower 95% CI | Upper 95% CI | t | p |
| --- | --- | --- | --- | --- | --- |
| (Intercept) | 0.032 | 0.028 | 0.037 | 13.755 | <0.001 |
| UA | 0.001 | -0.003 | 0.006 | 0.581 | 1.000 |
| FS | -0.001 | -0.005 | 0.002 | -0.771 | 1.000 |

| Random effects | Estimate | Lower 95% CI | Upper 95% CI |
| --- | --- | --- | --- |
| (Intercept) | 0.008 | 0.005 | 0.012 |
| Error | 0.032 | 0.030 | 0.033 |

**Detailed statistics: stand-to-sit, challenging condition, forward leaning strategy, synergy 3**

Reference group: UA

| Model information | Values |
| --- | --- |
| Number of observations | 1616 |
| Number of participants | 18 |
| ICC | 0.557 |

| Fixed effects | Estimate | Lower 95% CI | Upper 95% CI | t | p |
| --- | --- | --- | --- | --- | --- |
| (Intercept) | 0.032 | 0.027 | 0.038 | 11.290 | <0.001 |
| LT | 0.006 | 0.001 | 0.011 | 2.561 | 0.147 |
| FS | 0.005 | 0.000 | 0.009 | 2.051 | 0.567 |

| Random effects | Estimate | Lower 95% CI | Upper 95% CI |
| --- | --- | --- | --- |
| (Intercept) | 0.009 | 0.006 | 0.014 |
| Error | 0.033 | 0.032 | 0.034 |

Reference group: LT

| Fixed effects | Estimate | Lower 95% CI | Upper 95% CI | t | p |
| --- | --- | --- | --- | --- | --- |
| (Intercept) | 0.038 | 0.033 | 0.044 | 14.282 | <0.001 |
| UA | -0.006 | -0.011 | -0.001 | -2.561 | 0.147 |
| FS | -0.001 | -0.005 | 0.002 | -0.735 | 1.000 |

| Random effects | Estimate | Lower 95% CI | Upper 95% CI |
| --- | --- | --- | --- |
| (Intercept) | 0.009 | 0.006 | 0.014 |
| Error | 0.033 | 0.032 | 0.034 |

**Detailed statistics: stand-to-sit, challenging condition, forward leaning strategy, synergy 4**

Reference group: UA

| Model information | Values |
| --- | --- |
| Number of observations | 1616 |
| Number of participants | 18 |
| ICC | 0.500 |

| Fixed effects | Estimate | Lower 95% CI | Upper 95% CI | t | p |
| --- | --- | --- | --- | --- | --- |
| (Intercept) | 0.035 | 0.031 | 0.040 | 14.533 | <0.001 |
| LT | 0.001 | -0.003 | 0.005 | 0.375 | 1.000 |
| FS | -0.000 | -0.004 | 0.003 | -0.239 | 1.000 |

| Random effects | Estimate | Lower 95% CI | Upper 95% CI |
| --- | --- | --- | --- |
| (Intercept) | 0.007 | 0.005 | 0.011 |
| Error | 0.030 | 0.029 | 0.031 |

Reference group: LT

| Fixed effects | Estimate | Lower 95% CI | Upper 95% CI | t | p |
| --- | --- | --- | --- | --- | --- |
| (Intercept) | 0.036 | 0.032 | 0.041 | 15.988 | <0.001 |
| UA | -0.001 | -0.005 | 0.003 | -0.375 | 1.000 |
| FS | -0.001 | -0.005 | 0.002 | -0.704 | 1.000 |

| Random effects | Estimate | Lower 95% CI | Upper 95% CI |
| --- | --- | --- | --- |
| (Intercept) | 0.007 | 0.005 | 0.011 |
| Error | 0.030 | 0.029 | 0.031 |

**Detailed statistics: stand-to-sit, challenging condition, forward leaning strategy, synergy 6**

Reference group: UA

| Model information | Values |
| --- | --- |
| Number of observations | 1616 |
| Number of participants | 18 |
| ICC | 0.460 |

| Fixed effects | Estimate | Lower 95% CI | Upper 95% CI | t | p |
| --- | --- | --- | --- | --- | --- |
| (Intercept) | 0.034 | 0.030 | 0.038 | 15.283 | <0.001 |
| LT | -0.005 | -0.009 | -0.001 | -2.347 | 0.267 |
| FS | -0.004 | -0.008 | -0.000 | -2.182 | 0.409 |

| Random effects | Estimate | Lower 95% CI | Upper 95% CI |
| --- | --- | --- | --- |
| (Intercept) | 0.007 | 0.004 | 0.010 |
| Error | 0.029 | 0.028 | 0.030 |

Reference group: LT

| Fixed effects | Estimate | Lower 95% CI | Upper 95% CI | t | p |
| --- | --- | --- | --- | --- | --- |
| (Intercept) | 0.029 | 0.025 | 0.033 | 14.253 | <0.001 |
| UA | 0.005 | 0.001 | 0.009 | 2.347 | 0.267 |
| FS | 0.001 | -0.003 | 0.004 | 0.341 | 1.000 |

| Random effects | Estimate | Lower 95% CI | Upper 95% CI |
| --- | --- | --- | --- |
| (Intercept) | 0.007 | 0.004 | 0.010 |
| Error | 0.029 | 0.028 | 0.030 |

**Detailed statistics: stand-to-sit, challenging condition, forward leaning strategy, synergy 7**

Reference group: UA

| Model information | Values |
| --- | --- |
| Number of observations | 1616 |
| Number of participants | 18 |
| ICC | 0.674 |

| Fixed effects | Estimate | Lower 95% CI | Upper 95% CI | t | p |
| --- | --- | --- | --- | --- | --- |
| (Intercept) | 0.030 | 0.025 | 0.035 | 11.029 | <0.001 |
| LT | -0.004 | -0.008 | 0.000 | -1.940 | 0.737 |
| FS | -0.005 | -0.009 | -0.001 | -2.674 | 0.106 |

| Random effects | Estimate | Lower 95% CI | Upper 95% CI |
| --- | --- | --- | --- |
| (Intercept) | 0.009 | 0.006 | 0.014 |
| Error | 0.028 | 0.027 | 0.029 |

Reference group: LT

| Fixed effects | Estimate | Lower 95% CI | Upper 95% CI | t | p |
| --- | --- | --- | --- | --- | --- |
| (Intercept) | 0.026 | 0.021 | 0.031 | 10.130 | <0.001 |
| UA | 0.004 | -0.000 | 0.008 | 1.940 | 0.737 |
| FS | -0.001 | -0.004 | 0.002 | -0.686 | 1.000 |

| Random effects | Estimate | Lower 95% CI | Upper 95% CI |
| --- | --- | --- | --- |
| (Intercept) | 0.009 | 0.006 | 0.014 |
| Error | 0.028 | 0.027 | 0.029 |

**Detailed statistics: stand-to-sit, challenging condition, forward leaning strategy, synergy 8**

Reference group: UA

| Model information | Values |
| --- | --- |
| Number of observations | 1616 |
| Number of participants | 18 |
| ICC | 0.628 |

| Fixed effects | Estimate | Lower 95% CI | Upper 95% CI | t | p |
| --- | --- | --- | --- | --- | --- |
| (Intercept) | 0.027 | 0.020 | 0.033 | 8.006 | <0.001 |
| LT | 0.005 | -0.000 | 0.010 | 1.924 | 0.764 |
| FS | 0.002 | -0.003 | 0.007 | 0.899 | 1.000 |

| Random effects | Estimate | Lower 95% CI | Upper 95% CI |
| --- | --- | --- | --- |
| (Intercept) | 0.011 | 0.008 | 0.016 |
| Error | 0.036 | 0.035 | 0.037 |

Reference group: LT

| Fixed effects | Estimate | Lower 95% CI | Upper 95% CI | t | p |
| --- | --- | --- | --- | --- | --- |
| (Intercept) | 0.032 | 0.026 | 0.038 | 10.016 | <0.001 |
| UA | -0.005 | -0.010 | 0.000 | -1.924 | 0.764 |
| FS | -0.003 | -0.007 | 0.002 | -1.261 | 1.000 |

| Random effects | Estimate | Lower 95% CI | Upper 95% CI |
| --- | --- | --- | --- |
| (Intercept) | 0.011 | 0.008 | 0.016 |
